# Identifying the assembly intermediate in which Gag first associates with unspliced HIV-1 RNA suggests a novel model for HIV-1 RNA packaging

**DOI:** 10.1371/journal.ppat.1006977

**Published:** 2018-04-17

**Authors:** Brook C. Barajas, Motoko Tanaka, Bridget A. Robinson, Daryl J. Phuong, Kasana Chutiraka, Jonathan C. Reed, Jaisri R. Lingappa

**Affiliations:** Department of Global Health, University of Washington, Seattle, WA, United States of America; University of Illinois at Chicago College of Medicine, UNITED STATES

## Abstract

During immature capsid assembly, HIV-1 genome packaging is initiated when Gag first associates with unspliced HIV-1 RNA by a poorly understood process. Previously, we defined a pathway of sequential intracellular HIV-1 capsid assembly intermediates; here we sought to identify the intermediate in which HIV-1 Gag first associates with unspliced HIV-1 RNA. In provirus-expressing cells, unspliced HIV-1 RNA was not found in the soluble fraction of the cytosol, but instead was largely in complexes ≥30S. We did not detect unspliced HIV-1 RNA associated with Gag in the first assembly intermediate, which consists of soluble Gag. Instead, the earliest assembly intermediate in which we detected Gag associated with unspliced HIV-1 RNA was the second assembly intermediate (~80S intermediate), which is derived from a host RNA granule containing two cellular facilitators of assembly, ABCE1 and the RNA granule protein DDX6. At steady-state, this RNA-granule-derived ~80S complex was the smallest assembly intermediate that contained Gag associated with unspliced viral RNA, regardless of whether lysates contained intact or disrupted ribosomes, or expressed WT or assembly-defective Gag. A similar complex was identified in HIV-1-infected T cells. RNA-granule-derived assembly intermediates were detected *in situ* as sites of Gag colocalization with ABCE1 and DDX6; moreover these granules were far more numerous and smaller than well-studied RNA granules termed P bodies. Finally, we identified two steps that lead to association of assembling Gag with unspliced HIV-1 RNA. Independent of viral-RNA-binding, Gag associates with a broad class of RNA granules that largely lacks unspliced viral RNA (step 1). If a viral-RNA-binding domain is present, Gag further localizes to a subset of these granules that contains unspliced viral RNA (step 2). Thus, our data raise the possibility that HIV-1 packaging is initiated not by soluble Gag, but by Gag targeted to a subset of host RNA granules containing unspliced HIV-1 RNA.

## Author summary

During HIV-1 immature capsid assembly, packaging of the viral genome is initiated when the HIV-1 capsid protein, Gag, first associates with unspliced HIV-1 RNA. Although the complex in which this association initially occurs is critical for formation of infectious virus, the identity, composition, and the mechanism by which this complex forms remain unknown. To address this question, we utilized a previously described temporal pathway of intermediates in HIV-1 immature capsid assembly. The late intermediates in this pathway are derived from host RNA granules, which are diverse complexes utilized for cellular RNA storage and degradation. Here we sought to identify the intracellular capsid assembly intermediate in which HIV-1 Gag initially associates with unspliced HIV-1 RNA. We failed to detect an association between the first assembly intermediate, which contains soluble Gag, and unspliced HIV-1 RNA. Instead, the association between Gag and unspliced HIV-1 RNA was observed only in complexes corresponding to the RNA-granule-derived assembly intermediates. We also showed that Gag uses two determinants to form RNA-granule-derived intermediates that contain unspliced HIV-1 RNA. Together, these studies support a novel model for HIV-1 genome packaging, in which the first association between HIV-1 Gag and unspliced HIV-1 RNA occurs within a host RNA granule.

## Introduction

For released HIV-1 particles to be infectious, they must contain two copies of unspliced (full-length) HIV-1 RNA that are packaged during assembly of the immature HIV-1 capsid. Each immature capsid is composed of ~3000 copies of the HIV-1 structural protein Gag, which initially oligomerize in the cytoplasm and subsequently target to the plasma membrane (PM), where Gag multimerization is completed. Packaging of the viral genome is initiated when Gag first associates with unspliced viral RNA during assembly, and requires the nucleocapsid domain (NC) of Gag as well as specific encapsidation signals in unspliced HIV-1 RNA (reviewed in [1]). Immature capsids subsequently undergo budding, resulting in release of immature virus particles that contain the encapsidated genome and undergo maturation (reviewed in [2]). In the absence of unspliced HIV-1 RNA, Gag proteins assemble and release properly but the resulting virus-like particles are non-infectious [3].

In addition to being packaged, unspliced HIV-1 RNA is used for translation of Gag and GagPol (reviewed in [1]). It is generally agreed that translation and packaging are unlikely to occur concurrently, given that translation requires melting of secondary structures that are utilized during packaging; therefore translation and packaging are likely to be mutually exclusive (reviewed in [4,5]). However, the determinants that govern whether an unspliced HIV-1 RNA is utilized for translation or for packaging remain unclear. Mechanisms that have been proposed to explain how an unspliced HIV-1 RNA is directed towards packaging instead of translation include alternate RNA conformations that mask the translation start site and expose elements that favor packaging (reviewed in [5,6]); alternate 5' mRNA cap sequences that promote conformations favorable for packaging [7]; and inhibition of translation by accumulated Gag [8,9]. Additionally, it has been proposed that prior translation could lead to preferential packaging of an unspliced HIV-1 RNA [10]. However, other studies argue that *trans* packaging is the dominant packaging mechanism [11], and it is very clear that prior Gag translation is not necessary for packaging given that RNA generated from a lentiviral vector provided *in trans* can be packaged by Gag proteins synthesized from a different transcript (reviewed in [1,4]). Thus, in aggregate, the data argue that non-translating unspliced HIV-1 RNA undergoes packaging, and that one or more regulatory mechanisms play a role in determining whether a particular unspliced HIV-1 RNA is translated or packaged.

The process of packaging likely involves multiple steps that are closely coordinated with immature capsid assembly, culminating in complete encapsidation of the unspliced HIV-1 RNA within the fully assembled capsid. One would expect the first step of this process to involve association of Gag with unspliced HIV-1 RNA. Indeed, biochemical and imaging studies have supported the idea that association of Gag with unspliced viral RNA initiates the packaging process [12,13]. These studies demonstrated that Gag is likely a dimer or small oligomer when it first associates with unspliced HIV-1 RNA to initiate packaging; additionally, studies of an assembly-defective Gag mutant (Gag G2A) indicated that this initial association likely occurs in the cytoplasm [13]. The complex in which Gag first associates with unspliced HIV-1 genomic RNA to initiate packaging, here termed the packaging initiation complex, has not been identified, nor is it known how this complex is formed; additionally it is not known whether this complex contains only Gag or also contains cellular proteins. Answering these questions could lead to novel strategies for inhibiting genome encapsidation in infected cells. Despite this, to date, no study has even identified a candidate packaging initiation complex (let alone a definitive packaging initiation complex), leaving a large gap in our understanding of early events in the packaging process.

Because packaging occurs simultaneously with immature capsid assembly, insights into early events in packaging could be gained by studying the association of HIV-1 Gag with unspliced HIV-1 RNA during early events in immature capsid assembly. Here we leveraged our understanding of the sequence of events in HIV-1 immature capsid assembly to identify the earliest assembly itermediate in which HIV-1 Gag is associated with unspliced HIV-1 RNA. Previously, we showed that Gag progresses through a pathway of intracellular assembly intermediates consisting of sequential complexes of increasing size (referred to as the ~10S, ~80S, ~150S, and ~500S assembly intermediates) and culminating in formation of the ~750S completed immature capsid (reviewed in [14]). The sequential nature of these complexes was initially demonstrated by pulse-chase experiments, in cells and in cell extracts, which showed that over time newly synthesized Gag moves from an ~10S complex to an ~80S/150S complex, and then to an ~500S complex, before forming a completed ~750S immature capsid [15,16]. Two additional approaches confirmed the sequential order of these intracellular assembly intermediates that was predicted by pulse-chase studies. The first utilized an analysis commonly employed in the study of signaling pathways, in which the temporal order of events in a signaling pathway is determined by blockade of specific steps in that pathway. In an analogous manner, use of mutational blockade revealed that every assembly-defective Gag mutant studied to date is arrested at a specific point along the proposed capsid assembly pathway and forms only the intermediate at which it is arrested and the smaller intermediates that precede the point of arrest [15,17–20]. The second approach that confirmed the predicted temporal order of intermediates in this pathway involved defining the subcellular localization of these intermediates using biochemical as well as imaging approaches. These studies showed that, at steady state, the earliest intermediate (~10S) is largely cytosolic, the next intermediate (~80S/150S) is both in the cytosol and at the PM, and the final intermediate (~500S) is exclusively at the PM [19], thereby demonstrating the expected cytosol to PM progression of assembling Gag. Further support for the assembly intermediates being precursors to immature capsids came from the finding that the ~80S/150S and ~500S intermediates contain two other viral proteins found in the completed immature capsid: HIV-1 GagPol, which is present in a 1:20 ratio relative to Gag in assembly intermediates as is the case in released virus, and HIV-1 Vif [15,21]. Thus, together the pulse-chase, mutational, compositional, and subcellular localization analyses strongly support a temporal progression of assembling Gag from ~10S to ~80S/150S to 500S assembly intermediates, before formation of the ~750S completed immature capsids which subsequently undergo budding and release (reviewed in [14]).

Notably, studies of the assembly pathway had also revealed that the ~80S/150S, and ~500S intermediates contain host RNA granule proteins, such as DEAD-box RNA helicase 6 (DDX6) [22]. RNA granules are host ribonucleoprotein complexes that contain non-translating mRNA, in contrast to ribosomes, which contain translating mRNA (reviewed in [23]). Different classes of RNA granules exist, distinguished by their sizes and marker proteins, and functioning in silencing, storage, degradation, stress, and other events in RNA metabolism (reviewed in [23,24]). Some RNA granules, such as P bodies and stress granules, form relatively large foci that are easily visible by light microscopy, while others are smaller and poorly understood. Given that RNA granule proteins are not associated with HIV-1 Gag in the ~10S early assembly intermediate [19,20,22], these studies suggested that the ~80S HIV-1 assembly intermediate is formed when assembling Gag co-opts a poorly understood, small host RNA granule that contains canonical RNA granule proteins, such as DDX6. These Gag-containing granules also contain the host ATP-binding cassette protein E1 (ABCE1) [15,17–21], which has not been reported to be present in larger RNA granules. ABCE1 and DDX6 remain with Gag until immature capsid assembly is completed, at which point these host proteins dissociate [15,21,22] and the completed immature capsid undergoes budding and release.

Previous studies had also shown that, like the completed capsid, the ~80S/150S and ~500S assembly intermediates also contain unspliced HIV-1 RNA, as would be expected if they are precursors to the completed immature capsid. In these studies, an antibody directed against ABCE1, a cellular marker of assembly intermediates, was found to coimmunoprecipitate Gag as well as unspliced HIV-1 RNA from fractions containing the ~80S and ~500S assembly intermediates [19]. These findings are consistent with the assembly intermediates containing ABCE1, HIV-1 Gag, and unspliced HIV-1 RNA. However, they do not answer the key question of which assembly intermediate is the first to contain Gag associated with unspliced HIV-1 RNA, since the ~10S assembly intermediate does not contain ABCE1 and was therefore not immunoprecipitated in that study. Determining whether Gag associates with unspliced HIV-1 RNA in the earliest assembly intermediate (~10S intermediate) would require asking whether antibodies directed against Gag in the ~10S complex coimmunoprecipitate unspliced HIV-1 RNA. Importantly, this approach would also test between two fundamentally different models for the initial association of Gag with unspliced HIV-1 RNA during assembly. If Gag in the ~10S assembly intermediate is associated with unspliced HIV-1 RNA, that would suggest that unspliced HIV-1 RNA first associates with soluble Gag, given the small size of the ~10S complex (Model 1). Alternatively, if the ~80S and ~500S intermediates are associated with unspliced HIV-1 RNA but ~10S Gag is not, that would suggest that the first association of Gag with unspliced HIV-1 RNA occurs not in the soluble fraction, but within an assembly intermediate that is derived from a host RNA granule [22] (Model 2).

To date, studies have not tested between these two models for when and where assembling Gag first associates with unspliced HIV-1 RNA, each of which has compelling features. The soluble Gag model (Model 1) is appealing in its simplicity, and is consistent with longstanding studies showing that Gag associates specifically with unspliced HIV-1 RNA (reviewed in [1,25]). The RNA granule model (Model 2) is also consistent with these prior studies, but builds on the concept that in cells unspliced HIV-1 RNA likely behaves like cellular mRNA, which is found almost exclusively in ribonucleoprotein complexes. Cellular ribonucleoprotein complexes are generated during transcription, and undergo successive rounds of remodeling (reviewed in [26]), with their changing protein components dictating their changing fates (reviewed in [27]). In the RNA granule model, some of the ribonucleoprotein complexes that contain unspliced HIV-1 RNA would become translating complexes upon entering the cytoplasm, while others would form cytoplasmic non-translating complexes, including RNA granules. This model also suggests that Gag would have to localize to such non-translating host RNA granules in order to associate with the pool of unspliced HIV-1 RNA that is not occupied by translation machinery and to initiate interactions with that RNA. Having the first association between assembling Gag and unspliced HIV-1 RNA occur within RNA granules would provide numerous advantages to the nascent virus—for example, it could sequester unspliced HIV-1 RNA away from the host innate immune system, concentrate assembling Gag at a site rich in unspliced HIV-1 RNA, and place the Gag-RNA association and assembly in proximity with host enzymes that could facilitate those events. In keeping with the latter possibility, ABCE1 and DDX6, two of the host proteins present in both the assembly intermediates and host RNA granules, have been shown to facilitate immature HIV-1 capsid assembly [21,22] by mechanisms that remain to be determined.

To determine whether Gag first associates with unspliced HIV-1 RNA in the earliest assembly intermediate (~10S Gag-containing complex) or in an RNA-granule-derived intermediate (~80S or 500S Gag-containing complex), here we sought to identify all non-nuclear cellular complexes that contain unspliced HIV-1 RNA, as well as the subset of these complexes that contains Gag in association with unspliced HIV-1 RNA. First we found that, in the presence or absence of assembling Gag, essentially all non-nuclear unspliced HIV-1 RNA, whether translating or non-translating, is in complexes ≥30S. Next we showed that in lysates of cells expressing proviral Gag or Gag expressed with a genomic construct *in trans*, the ~80S assembly intermediate was the smallest previously described assembly intermediate in which Gag was associated with unspliced viral RNA at steady state. Unspliced HIV-1 RNA was found in the ~80S assembly intermediate regardless of whether the experiment was performed in the absence or presence of PuroHS, which was used to disrupt translating ribosomes. Additionally, in cells expressing Gag mutants that associate with unspliced viral RNA but are arrested at early stages of assembly, the ~80S, but not the ~10S assembly intermediate, contained unspliced HIV-1 RNA. Notably, we found no evidence for unspliced HIV-1 RNA in association with ~10S Gag at steady state under any experimental condition, despite the presence of abundant ~10S Gag in all our experiments. Thus, our data favor Model 2, which proposes that the RNA granule derived ~80S assembly intermediate is the complex in which Gag first associates with unspliced HIV-1 RNA. Consistent with Model 2, we also found that DDX6 and ABCE1, which facilitate assembly and are present in RNA-granule-derived assembly intermediates, are associated with unspliced HIV-1 RNA in fractions containing the ~80S assembly intermediate, as would be expected. Also consistent with Model 2, in chronically infected human T cells, unspliced HIV-1 RNA was found associated with ABCE1 in fractions containing the ~80S assembly intermediate. *In situ* studies confirmed the association of RNA granule proteins with unspliced HIV-1 RNA at PM sites of budding, and the colocalization of assembly-competent Gag with RNA granule proteins. Our *in situ* experiments also revealed that complexes containing the RNA granule protein DDX6 colocalized with Gag are far more numerous than P bodies, and likely correspond to small RNA granules that are visible as fluorescent foci. Finally, we demonstrated that assembling Gag uses a two-step process to localize to a subset of RNA granules that contains unspliced HIV-1 RNA, leading to formation of the ~80S assembly intermediate that contains Gag associated with unspliced HIV-1 RNA. One of these steps is dependent on binding to HIV-1 RNA, as would be expected. However, the other step that Gag uses to target to RNA granules is independent of HIV-1 RNA binding, suggesting a novel and poorly understood mechanism for localizing assembling Gag to the subset of RNA granules that contains unspliced HIV-1 RNA. Together, our data support a model in which Gag associates with unspliced HIV-1 RNA within a poorly understood subclass of host RNA granules. These findings advance our understanding of RNA packaging by identifying a candidate complex in which packaging may be initiated and by raising the possibility that packaging is initiated within host RNA granules.

## Results

### Confirmation of phenotypes for proviruses expressing WT Gag and Gag mutants

**Fig 1:**
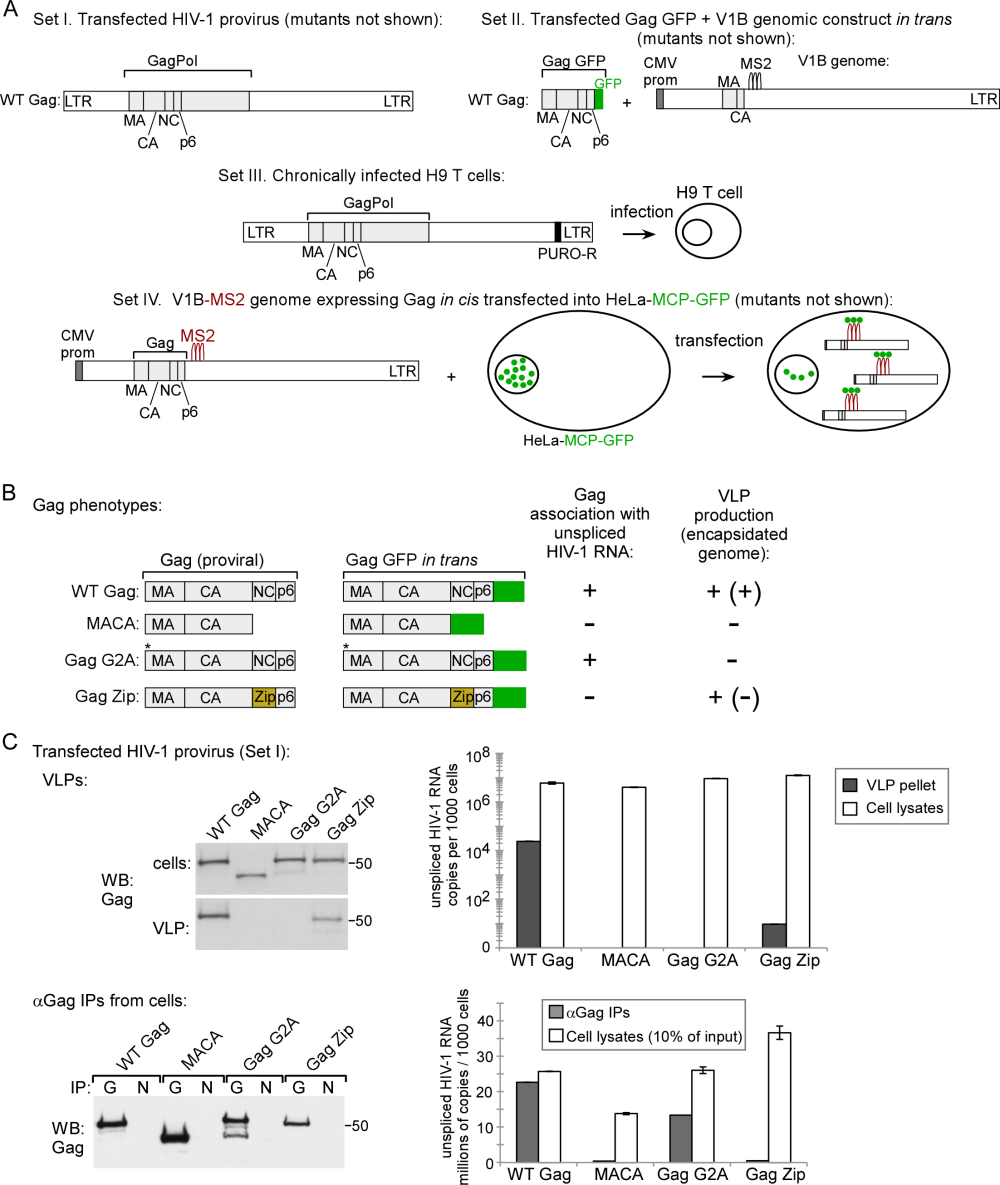
Gag constructs: Diagrams and phenotyopes. **(A)** Diagram of the different *cis* and *trans* expression systems used (Sets I–IV). Only WT constructs are shown here, with mutant constructs diagrammed in later figures. **Set I** consists of WT (and mutant) HIV-1 proviruses (*pro-*, delta *env*). **Set II** consists of codon-optimized WT (and mutant) Gag constructs tagged with GFP (Gag GFP) that are co-transfected with a modified genomic construct (V1B). V1B provides the genome for packaging *in trans* and expresses an assembly-defective truncated Gag. **Set III** consists of a WT provirus from Set I in which the *nef* gene was replaced with a puromycin resistance gene (PURO-R). H9 T cells were infected with this construct and maintained under puromycin selection to generate a chronically infected H9 T cell line expressing WT Gag from a provirus. **Set IV** consists of V1B constructs (see Set II) which were engineered to express WT or mutant Gag *in cis* (rather than the assembly-defective truncated Gag expressed by the Set II V1B construct). Set IV constructs were transfected into HeLa-MCP-GFP cells, which express a GFP-tagged MS2 capsid protein (MCP) that contains a nuclear localization signal. Since V1B genomic constructs also contain MS2 binding sites, MCP-GFP binds to V1B, resulting in GFP tagging of V1B viral RNA [12]. **(B) Summary of expected Gag phenotypes.** Diagram indicates whether WT Gag or each Gag mutant is expected to associate with intracellular unspliced HIV-1 RNA, leading to packaging initiation, and whether VLPs are known to be produced. Released VLPs that contain or lack viral RNA are indicated by +(+) and +(-), respectively. **(C) Confirmation of expected Gag phenotypes.** COS-1 cells were transfected with Set I constructs from A. Cell lysates and VLPs were harvested for analysis. Top row: Equivalent aliquots of cell lysates were analyzed by WB for Gag, as were VLPs harvested from the corresponding cell supernatants. VLPs and cell lysates were also analyzed by RT-qPCR for copies of unsplied HIV-1 RNA (graph). Similar results were obtained in 293T cells but are not presented. Bottom row: Cell lysates expressing proviruses encoding WT or mutant Gag (Set I constructs in panel A) were subjected to IP with αGag (G) or nonimmune (N) antibody followed by Gag WB (left). IP eluates were also analyzed by RT-qPCR for copies of unspliced HIV-1 RNA, with NI values subtracted (graph). Error bars show SEM from duplicate samples. Positions of kD markers are shown to the right of blots. Data are representative of three independent replicate experiments.

To study the association of HIV-1 Gag with unspliced HIV-1 RNA in HIV-1 capsid assembly intermediates, we used a variety of previously published HIV-1 expression systems (Fig 1A, sets I—IV); these produce WT Gag or Gag mutants with known phenotypes for production of virus-like particles (VLPs) that either do or do not contain the genome (Fig 1B). Given these known VLP phenotypes and an understanding of the specific Gag mutant defect, one can also predict phenotypes for intracellular association of Gag with unspliced HIV-1 RNA. Thus, because WT Gag expression results in release of VLPs that contain unspliced HIV-1 RNA, one would also expect to find intracellular complexes containing Gag associated with unspliced HIV-1 RNA (Fig 1B). In contrast, assembly-defective Gag mutants do not produce VLPs, but whether these Gag mutants would be expected to associate with intracellular unspliced HIV-1 RNA depends on their exact defect (Fig 1B). Specifically, the assembly-incompetent truncated Gag MACA mutant, which is arrested as a soluble ~10S assembly intermediate, would not be expected to associate with unspliced HIV-1 RNA because it lacks the RNA-binding NC domain (reviewed in [14,25,28]). In contrast, Gag G2A, which is arrested in the cytoplasm due to a point mutation that prevents the myristoylation required for PM targeting and VLP production [29–31], would be expected to associate with intracellular unspliced HIV-1 RNA (Fig 1B), as shown previously [13]. We also examined the HIV-1 Gag Zip chimera, in which the RNA-binding NC domain of Gag is replaced with a dimerizing leucine zipper (LZ) that allows for capsid assembly but not RNA association. Gag Zip produces VLPs that lack unspliced HIV-1 RNA [18,32–34] and would not be expected to associate with unspliced HIV-1 RNA (Fig 1B). Gag Zip is of interest because it forms assembly intermediates even though it fails to package unspliced viral RNA [18]; thus, Gag Zip could provide insights into how association with unspliced viral RNA can fail to occur during Gag assembly.

To confirm the VLP production phenotypes of these Gag proteins and define their association with intracellular unspliced HIV-1 RNA, COS-1 cells were transfected with proviral constructs expressing WT Gag or these Gag mutants (Set I constructs in Fig 1A). VLP phenotypes were determined by analyzing transfected cell lysates and VLPs for Gag levels by Western blot (WB), and for copies of unspliced HIV-1 RNA by reverse transcription followed by quantitative PCR (RT-qPCR; Fig 1C, top row). Association of WT Gag and Gag mutants with intracellular unspliced HIV-1 RNA was assessed by immunoprecipitation (IP) with antibody directed against Gag (αGag) followed by RT-qPCR to quantify the number of unspliced HIV-1 RNA copies associated with Gag in cell lysates (Fig 1C, bottom row). Results of these assays confirmed the known phenotypes for VLP production and the expected phenotypes for association with intracellular unspliced HIV-1 RNA. Thus, these four constructs display a range of phenotypes, with assembly-competent WT Gag and assembly-defective Gag G2A associating with intracellular unspliced HIV-1 RNA, but assembly-incompetent MACA and assembly-competent Gag Zip not associating with intracellular unspliced HIV-1 RNA, as expected (summarized in Fig 1B).

### Non-translating unspliced HIV-1 RNA is in large complexes even in the absence of assembling Gag

**Fig 2:**
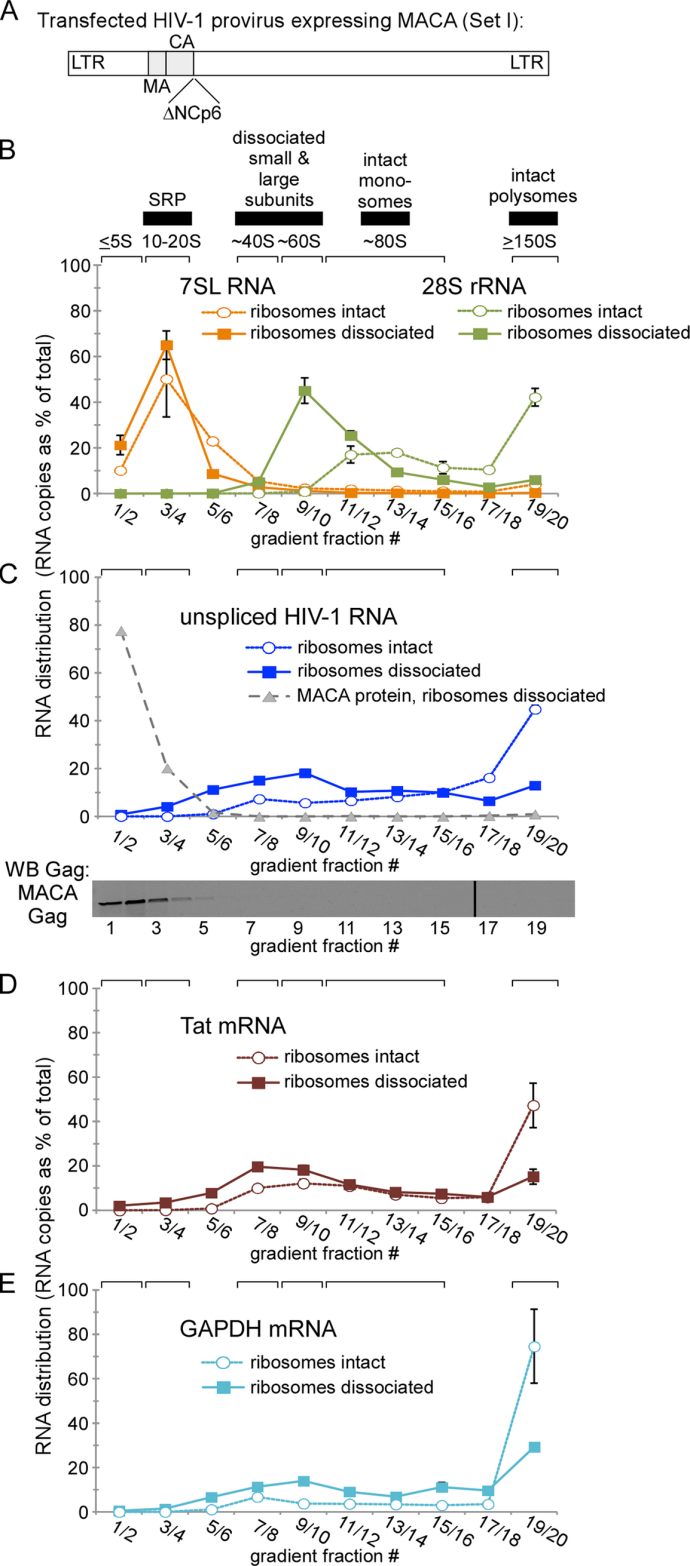
Non-translating unspliced HIV-1 RNA is primarily in diverse complexes ≥30S in the absence of assembling Gag. **(A-E)** Lysate from COS-1 cells transfected with the assembly-incompetent MACA provirus (Set I constructs in Fig 1A) was divided into two pools that were either untreated (ribosomes intact) or treated with PuroHS (ribosomes dissociated). Both pools were analyzed in parallel by velocity sedimentation followed by RT-qPCR of paired gradient fractions using the appropriate qPCR primer sets to determine copy number of the indicated RNA (28S rRNA, 7SL RNA, unspliced HIV-1 RNA, HIV-1 Tat mRNA, or GAPDH mRNA). Quantity of the indicated RNA in gradient fractions is expressed as a distribution (% of RNA in all fractions) to allow comparison between different species of RNA. Blot in panel C shows migration of MACA protein, which is also graphed as a gray dotted line in C. Position of kD marker is shown to the right of the blot. Brackets at top show expected S value migrations, and horizontal bars show expected migrations of various ribonucleoprotein complexes. Error bars show SEM from duplicate samples. Data are from a single experiment that is representative of three independent replicate experiments.

Next we defined the spectrum of cytoplasmic complexes that contain unspliced HIV-1 RNA in the absence of assembling Gag. For this purpose, we transfected cells with MACA provirus (Fig 2A; Set I constructs in Fig 1A), which expresses an otherwise full-length viral RNA encoding a truncated MACA Gag protein that is assembly-incompetent [17–19,35–38], is arrested at the first assembly intermediate (the ~10S complex; [19]), and does not associate with unspliced viral RNA (Fig 1C, bottom row). We chose not to use a provirus that contains a premature stop codon in Gag because stop codons early in Gag typically result in synthesis of a poorly-studied N-terminally truncated Gag protein that starts from a downstream internal AUG codon [10,39]. Instead, we used a provirus that produces the well-studied assembly-defective MACA Gag mutant that does not associate with unspliced HIV-1 RNA (Fig 1C). Note that in all our RNA quantification experiments, we removed nuclei by centrifugation, allowing us to focus on non-nuclear RNA, including cytoplasmic RNA as well as membrane-associated RNA that was solubilized with non-ionic detergent during the harvest. In our initial experiments, these lysates contained both translating and non-translating complexes, and were analyzed using velocity sedimentation on gradients that resolved complexes in the ~5S to ~150S range, followed by RT-qPCR to determine the approximate S values of complexes containing specific types of RNA. Previously, we have used a combination of mathematical approaches (McEwen method; [40]) and analysis of complexes with known S values to define migrations in these gradients [41]. In all our gradients, we name a complex by the S value of the fraction in which it peaks; thus a complex that spans the ~40-80S region but peaks in the ~80S region is called an ~80S complex. Wide peaks can result when particular methods are used (e.g. when fewer fractions are taken or when harvest conditions partially disrupt the integrity of protein complexes, as discussed below).

Here, we confirmed these expected migrations by analyzing gradient fractions for types of cellular RNA with known S values (Fig 2B, open symbols). To identify small ribonucleoprotein complexes, we analyzed 7SL RNA, a component of signal recognition particle, which contains six proteins and one RNA and migrates at ~11S [42]. To identify the expected position of larger complexes, we quantified 28S ribosomal RNA (rRNA), which marks the position of the large ribosomal subunit (60S), monosomes (80S), and polysomes (160S and larger). When lysates expressing the MACA provirus were analyzed under conditions that leave ribosomes intact, 7SL RNA was found almost entirely in the 10-20S region (fractions 3/4; Fig 2B, open orange circles), while 28S rRNA was found in two peaks, one centered around the mathematically predicted ~80S region (fractions 11–14), representing monosomes, and one in the predicted ≥ 150S region (fractions 19/20), representing polysomes (Fig 2B, open green circles). Thus, under standard harvest conditions, most ribosomes were in the form of intact monosomes and polysomes.

Having confirmed the migration of particles of diverse sizes, we then examined the same fractions for unspliced HIV-1 RNA (Fig 2C, open blue circles) and found that it was broadly distributed in fractions ≥40S (fractions 7 through 20), with a large peak in the region corresponding to polysomes (≥150S, fractions 19/20). Notably, unspliced HIV-1 RNA was not observed in fractions 1–6, which contain particles of <40S and soluble complexes. As an additional marker for the soluble fraction, we also examined these gradient fractions for MACA Gag by WB (Fig 2C, grey triangles); as expected, the truncated, assembly-incompetent MACA Gag protein was found entirely in fractions 1–4 (with its peak in fractions 1/2), further confirming that this represents the soluble fraction. Thus, it appears that all detectable unspliced HIV-1 RNA is in complexes >40S (fractions 7–20) that include both translating complexes (in ~80S monosomes and a prominent ≥150S polysome peak) as well as non-translating complexes of diverse sizes. To assess whether unspliced HIV-1 RNA is in the same fractions as mRNA, we also examined the migration of one subgenomic viral mRNA (Tat mRNA) and the cellular mRNA for GAPDH (Fig 2D and 2E, respectively, open symbols). We found that, like unspliced HIV-1 RNA, viral and cellular mRNA migrated in the ≥ 40S region (fractions 7–20), with a prominent peak in the position of polysomes (fractions 19/20), and no significant signal in the <40S region (fractions 1–6). Thus, these findings indicated that cellular and viral mRNA (e.g. GAPDH and Tat mRNA, respectively) are found largely in translating and non-translating complexes of diverse sizes and are not found in the soluble fraction of the cytoplasm. Moreover, we concluded that complexes containing unspliced HIV-1 RNA have the same size distribution as complexes containing cellular mRNA (or subgenomic viral mRNA) when assembling Gag is absent and ribosomes are intact; additionally, to the level of our detection, unspliced HIV-1 RNA is either in translating complexes (which are mainly polysomes) or in non-translating ribonucleoprotein complexes ≥ 40S, but is largely absent from the soluble fraction. Our findings are consistent with a previous study that found no HIV-1 RNA in the soluble fraction of cell lysates [43]. Our data also demonstrated that following standard cell lysis and velocity sedimentation, ribonucleoprotein complexes of different sizes (monosomes, polysomes, and SRP) remain intact and retain their expected S values.

Our next goal was to extend this analysis to lysates of cells transfected with proviruses expressing WT Gag or Gag mutants that associate with unspliced HIV-1 RNA, so that we could identify the smallest HIV-1 capsid assembly intermediate containing Gag that is associated with unspliced HIV-1 RNA by αGag IP. However, first we needed to address the problem that αGag would be expected to immunoprecipitate both non-translating complexes containing unspliced HIV-1 RNA (e.g. assembly intermediates) and actively translating complexes in which Gag epitopes are exposed while Gag is still being synthesized from the unspliced mRNA template. These translating and non-translating complexes would be indistinguishable in our IP-RT-qPCR analyses. Thus, to identify all non-translating complexes containing Gag associated with unspliced HIV-1 RNA in provirus expressing cells, we needed a way to eliminate complexes involved in Gag translation from our analyses since these complexes are unlikely to be involved in assembly or packaging for reasons described above.

We reasoned that the best way to remove translating Gag associated with unspliced HIV-1 RNA would be to efficiently disrupt ribosomes and leave intact the complexes that contain non-translating unspliced HIV-1 RNA. Treatment of cell lysates with *puro*mycin and *h*igh *s*alt (PuroHS) has long been used to disrupt translating ribosomes, resulting in release of translating polypeptides (e.g. nascent Gag) from ribosomes, dissociation of functional ribosomal subunits, and release of free mRNA [44,45], which then likely shifts into non-translating ribonucleoprotein complexes [46]. Thus, complexes that contain unspliced HIV-1 RNA and remain intact after PuroHS treatment would be expected to be mainly non-translating complexes. Moreover, a subset of those non-translating complexes likely corresponds to assembly intermediates containing Gag associated with unspliced HIV-1 RNA.

Before utilizing PuroHS to identify all assembly intermediates containing Gag associated with unspliced HIV-1 RNA, we first determined the efficacy of this treatment for ribosome disruption by analyzing the effect of PuroHS on the HIV provirus in absence of assembling Gag. Gradient fractions of cell lysates expressing the MACA provirus (diagrammed in Fig 2A) and harvested after PuroHS treatment were analyzed for each RNA of interest (Fig 2B–2E, solid symbols; note that PuroHS treatment was performed and analyzed in parallel with lysates harvested in the absence of PuroHS treatment, which are shown in Fig 2 with open symbols and described above.) As described above, in the absence of PuroHS treatment, 28S rRNA (a marker for the 60S large ribosomal subunit; Fig 2B, open green circles) migrated almost entirely in the position of monosomes (~80S) and polysomes (>150S). In contrast, following PuroHS treatment, 28S rRNA migrated almost entirely in a ~60S peak representing the dissociated large ribosomal subunit (Fig 2B, solid green squares), with almost no 28S rRNA remaining in the polysome region (>150S). The near complete absence of 28S rRNA (i.e. the large ribosomal subunit rRNA) in the polysome region after PuroHS treatment indicated highly effective ribosome disassembly. Thus, it appears that PuroHS disassembles most monosomes and polysomes into ribosomal subunits. PuroHS treatment did not affect the migration of 7SL RNA (Fig 2B, compare open orange circles vs. solid orange squares), as expected given that 7SL RNA is a component of the ribosome-independent ~11S signal recognition particle [42]. From these data, we conclude that treatment of cell lysates with PuroHS disrupts translating ribonucleoprotein complexes very effectively but has little effect on non-translating, ribosome-independent complexes, such as signal recognition particle.

We also examined unspliced HIV-1 RNA in these same MACA-containing gradient fractions following PuroHS treatment, and found that > 95% of this RNA was in diverse ribonucleoprotein complexes of ≥ 30S (fractions 5–20) that formed a broad peak centered at ~60S (Fig 2C, solid blue squares). Overall, more unspliced HIV-1 RNA was found in the ~40S to ~60S region following PuroHS treatment, consistent with PuroHS treatment causing disassembly of ribosomes into ribosomal subunits. Comparison of the distribution of RNA obtained with and without PuroHS treatment suggested that at least 30% of total unspliced HIV-1 RNA was initially in translating polysomes, defined as complexes ≥150S that are lost upon PuroHS treatment. Given that PuroHS treatment almost completely dissociated ribosomes (as measured by 28S rRNA) in the ≥ 150S polysome fraction (Fig 2B, compare solid green squares to open green circles), these data indicate that, following PuroHS treatment, unspliced HIV-1 RNA shifts from polysomes (≥150S) into non-translating ribonucleoprotein complexes of ~30-150S, some of which are present even in the absence of PuroHS treatment and overlap in size with translating complexes (Fig 2C, compare solid blue squares to open blue circles). A similar shift out of polysomes and into the ~30-150S size range of non-translating ribonucleoprotein complexes was observed for subgenomic HIV-1 Tat mRNA and cellular GAPDH mRNA (Fig 2D and 2E, compare solid squares to open circles). Note that because PuroHS treatment was performed after cells were lysed and lysates were clarified to remove nuclei and large organelles, it is unlikely that PuroHS resulted in formation of stress granules or P bodies. Moreover, our finding that unspliced and spliced HIV-1 RNA as well as a cellular GAPDH mRNA shifted to smaller complexes after PuroHS treatment rather than larger complexes (Fig 2) is consistent with this conclusion, given that stress granules and P bodies are very large. Together, these studies reveal that non-translating unspliced HIV-1 RNA, like cellular mRNA and subgenomic viral mRNA, are found in complexes ≥ 30S under standard harvest conditions, and that these non-translating complexes become more abundant following disruption of ribosomes in lysates with PuroHS.

### The only assembly intermediate that contains Gag G2A associated with unspliced HIV-1 RNA is the ~80S assembly intermediate

Our finding that, in the absence of assembling Gag, translating and non-translating unspliced HIV-1 RNA are found almost entirely in ≥ 30S complexes indicates that like cellular mRNA, HIV-1 RNA is sequestered within either translating or non-translating host ribonucleoprotein complexes. Next, we assessed the association of unspliced HIV-1 RNA with WT Gag or assembly-defective Gag in each assembly intermediate. When proviruses encoding WT Gag are expressed, much of the steady state Gag is found in the ~10S assembly intermediate, which likely contains a monomer or dimer of Gag, while the remainder is found in the RNA-granule-derived ~80S/150S and ~500S assembly intermediates. We had previously showed that because assembly-defective Gag mutants are arrested at key steps in the assembly pathway, Gag mutants can effectively trap Gag in a subset of assembly intermediates [15,18–20,47].

**Fig 3:**
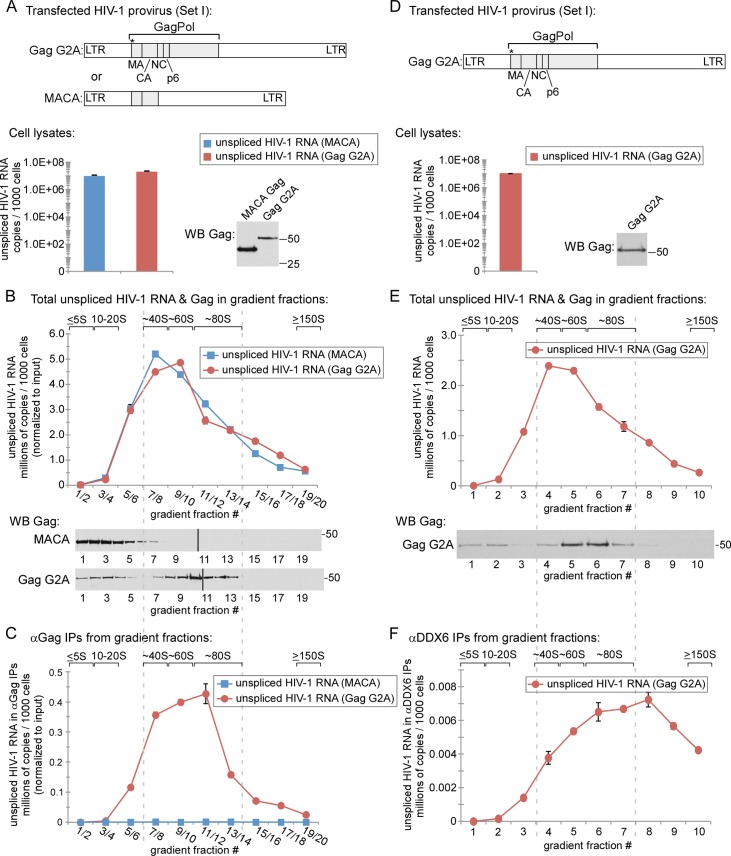
The ~80S assembly intermediate contains Gag G2A associated with unspliced HIV-1 RNA while the ~10S Gag G2A assembly intermediate does not. **(A)** COS-1 cells transfected with indicated MACA and G2A proviruses (Set I constructs in Fig 1A) were harvested following PuroHS treatment, and the number of unspliced HIV-1 RNA copies was determined for cell lysates. (**B)** Lysates from A were analyzed by velocity sedimentation, and the number of unspliced HIV-1 RNA copies per 1000 cells was determined for each fraction and normalized to the inputs shown in A. (**C)** Gradient fractions from B were subjected to IP with human polyclonal HIV immune globulin (αGag), and the number of unspliced HIV-1 RNA copies per 1000 cells was determined for IP eluates from each gradient fraction and normalized to the inputs shown in A. Similar results were obtained upon IP with monoclonal antibody to p24 but are not presented. (**D)** COS-1 cells transfected with the indicated Gag G2A provirus (Set 1 constructs in Fig 1A) were harvested following PuroHS treatment, and the number of unspliced HIV-1 RNA copies per 1000 cells was determined for cell lysate. (**E)** Lysate from D was also analyzed by velocity sedimentation, and viral RNA copy number per 1000 cells was determined for each gradient fraction. (**F)** Gradient fractions from E were subjected to IP with αDDX6, and the number of unspliced HIV-1 RNA copies per 1000 cells was determined for IP eluates from each gradient fraction. Positions of kD markers are shown to the right of blots. Brackets at top show S value markers, and dotted lines demarcate assembly intermediates based on their migrations in the corresponding Gag WB. Error bars show SEM from duplicate samples. Data in each column are from a single experiment that is representative of three independent replicate experiments.

We began these analyses with the well-studied, targeting-defective Gag G2A mutant, described above. Others have used Gag G2A to demonstrate that the initial association of Gag with unspliced HIV-1 RNA occurs in the cytoplasm [13]. Given those previous data and our confirmation that Gag G2A associates with unspliced HIV-1 RNA in the cytoplasm by αGag IP (Fig 1C), we expected Gag G2A to form at least one assembly intermediate that contains Gag associated with unspliced HIV-1 RNA. We had previously observed that Gag G2A forms the first two intermediates in the pathway, soluble ~10S Gag and the RNA-granule-derived ~80S intermediate; thus, we reasoned that either the ~10S or ~80S assembly intermediate is likely to be the intermediate in which Gag first associates with unspliced HIV-1 RNA. While the arrested ~80S Gag G2A assembly intermediate is clearly defective, it nevertheless closely resembles the WT ~80S assembly intermediate, both in its size and composition [19,20,22] and would therefore be useful for identifying the earliest assembly intermediate in which Gag first associates with unspliced HIV-1 RNA. To allow us to focus only on assembly intermediates (which are non-translating complexes), we treated lysates with PuroHS during harvest to eliminate translating complexes that could contain Gag associated with translating unspliced HIV-1 RNA (as shown in Fig 2). We first confirmed the known distributions of MACA and Gag G2A protein by analyzing PurosHS-treated lysates of cells transfected with proviruses to express MACA or Gag G2A (Set I constructs in Fig 1A) to similar steady state levels (Fig 3A). As expected, when these lysates were analyzed by velocity sedimentation followed by WB of gradient fractions, the distribution of the MACA vs. Gag G2A protein across the gradient differed dramatically, with MACA protein forming only the ~10S intermediate (Fig 3B, MACA WB, fractions 1–4, with a trail in 5–7), while Gag G2A formed both the soluble ~10S intermediate and a complex that spans the ~60-80S region and corresponds to the ~80S assembly intermediate (Fig 3B, G2A WB, fractions 7–13, peak in fraction 10). In contrast, both MACA and Gag G2A lysates displayed the same distribution of non-translating unspliced HIV-1 RNA across the gradient (Fig 3B, graph), with unspliced HIV-1 RNA from both lysates mainly in the ~40-80S fractions (and peaking at ~60S) and almost no unspliced HIV-1 RNA in the soluble fractions (fractions 1–4). Thus, the only population of Gag protein that co-migrated with the non-translating unspliced viral RNA peak was Gag G2A in the ~60-80S fractions, with neither soluble Gag G2A nor soluble MACA co-migrating with non-translating unspliced HIV-1 RNA.

Having demonstrated that Gag G2A in the ~60-80S region of the gradient co-migrates with a population of unspliced viral RNA, we next asked whether Gag G2A in this region is actually associated with unspliced HIV-1 RNA, or simply co-migrates in a separate complex. For this purpose, gradient fractions from Fig 3B were subjected to αGag IP, followed by quantitation of viral RNA in IP eluates (Fig 3C). Note that all IP analyses in this study were performed under native conditions, and are thus expected to pull down the protein targeted by the antibody as well as any other components that are stably associated with the target protein through direct or indirect interactions. Even though αGag coimmunoprecipitated MACA effectively (Fig 1C), no unspliced HIV-1 RNA was associated with MACA by αGag IP in any fraction (Fig 3C), as expected given that the MACA peak does not co-migrate with unspliced HIV-1 RNA (compare Fig 3B graph to Fig 3B blot). Similarly, soluble Gag G2A protein (in the ≤ 20S region) was associated with little or no unspliced HIV-1 RNA by coimmunoprecipitation (coIP) (Fig 3C). Indeed, αGag IP from fractions containing ≤ 20S Gag G2A (fractions 1–4 in Fig 3C) contained 2.2 copies of unspliced HIV-1 RNA per fraction per cell. Given that the limit of detection in gradient IP samples is 1 copy per cell (as indicated by standard curves run with every assay) and given that our samples are loaded at the top of the gradient and would be expected to leave behind some contamination in the top fractions (fractions 1 and 2), these data indicate that the substantial pool of soluble Gag G2A proteins in fractions 1–4 is not associated with unspliced HIV-1 RNA. In contrast, the αGag IP of Gag G2A from ~80S fractions (fractions 11–14 in Fig 3C) contained an average of 285 copies of unspliced HIV-1 RNA per fraction per cell. Thus, to the limit of detection of our very sensitive assay, we were unable to identify a significant amount of unspliced HIV-1 RNA associated with soluble Gag G2A; instead, we found that unspliced HIV-1 RNA was strongly associated with a pool of Gag G2A that peaks at ~80S and likely corresponds to the ~80S intermediate (Fig 3C, graph), which is the second intermediate in the assembly pathway. These findings suggest that the ~80S assembly intermediate is the first intermediate in the previously described assembly pathway that contains Gag associated with unspliced HIV-1 RNA.

Previously, we had demonstrated that the ~80S assembly intermediate formed by Gag G2A and WT Gag contains host proteins, including the RNA granule protein DDX6 and the cellular ATPase ABCE1, as shown by coIP of both Gag proteins with antibodies to DDX6 (αDDX6; [22]) and ABCE1 (αABCE1; [21]). DDX6, a cellular RNA helicase found in P bodies, is involved in mRNA silencing and mRNA storage, but is not typically associated with actively translating mRNA [48]. The association of HIV-1 Gag with DDX6 is RNase-sensitive [22], thus DDX6 does not bind directly to Gag. Importantly, the presence of DDX6 in the ~80S assembly intermediate suggests that Gag co-opts a host RNA granule to generate the ~80S intermediate [22]. Thus, if the ~80S complex containing Gag G2A associated with unspliced HIV-1 RNA in Fig 3C corresponds to the ~80S assembly intermediate, then unspliced viral RNA in the ~80S fractions should be associated with DDX6. In PuroHS-treated lysates of cells expressing G2A provirus (Fig 3D), unspliced HIV-1 RNA was again observed almost exclusively in the ~40-80S region of gradients (Fig 3E, compare to Fig 3B). Additionally, IP with αDDX6 revealed that unspliced HIV-1 RNA is associated with DDX6 in a broad complex that peaks at a point slightly larger than ~80S, confirming that unspliced HIV-1 RNA is associated with DDX6 in fractions containing the ~80S assembly intermediate (Fig 3F). While we would not expect DDX6 to be associated with ribosomes, our use of PuroHS-treated lysates allows us to confirm that the ~80S complex containing DDX6 associated with unspliced HIV-1 RNA in Fig 3F is not a translating complex. Taken together with our previous coIP analyses showing that Gag G2A is associated with DDX6 [22], these data suggest that the Gag G2A ~80S assembly intermediate likely contains non-translating unspliced HIV-1 RNA in association with Gag G2A and the RNA granule protein DDX6. These data are consistent with the ~80S assembly intermediate, not the ~10S intermediate, being the first assembly intermediate that contains unspliced HIV-1 RNA in association with Gag G2A.

### The WT ~80S and ~500S assembly intermediates contain Gag associated with unspliced HIV-1 RNA while the WT ~10S assembly intermediate does not

Although the Gag G2A mutant has been used by others to study the initial association of Gag G2A with unspliced HIV-1 RNA [13], it is formally possible that Gag G2A, being a mutant, forms an ~10S complex containing Gag G2A associated with unspliced HIV-1 RNA that is unstable and was therefore not detected at steady state in our experiments; alternatively, the ~80S intermediate that contained Gag G2A associated with unspliced HIV-1 RNA could be an abnormal complex unique to the G2A mutant. For these reasons, we next asked which assembly intermediates formed by WT Gag contain unspliced HIV-1 RNA associated with Gag. Like Gag G2A, WT Gag forms an ~80S assembly intermediate; unlike Gag G2A, WT Gag also forms an ~500S assembly intermediate after membrane targeting [19,20,22]. However, to ask whether Gag is associated with unspliced HIV-1 RNA in all of these assembly intermediates, we needed a method for immunoprecipitating multimerized ~500S Gag because diverse Gag antibodies fail to immunoprecipitate multimerized Gag due to epitope masking, even though they successfully immunoprecipitate soluble Gag and oligomerized Gag [49]. For this reason, we utilized antibodies to GFP (αGFP) to immunoprecipitate Gag GFP from lysates of cells co-transfected with GFP-tagged, codon-optimized Gag (Gag GFP) and VIB, a modified proviral construct that expresses a viral RNA that contains all the signals needed for packaging [12,13] and has been successfully coimmunoprecipitated with αGFP [13]. Notably, the V1B viral genome encodes a truncated assembly-incompetent Gag that does not interfere with assembly of Gag GFP expressed *in trans*. Co-transfection of Gag GFP and the V1B genome (Set II constructs in Fig 1A) has been well vetted in live imaging and biochemical studies [12,13]. Moreover, use of this *in trans* system would also allow us to express a single well-studied V1B genomic construct with different Gag constructs (using qPCR oligos that detect cDNA made from unspliced VIB RNA but not cDNA made from Gag GFP mRNA). As expected, we found that co-transfection of Gag GFP and V1B plasmids resulted in the same phenotypes for VLP production and Gag association with intracellular unspliced viral RNA that were observed for proviral constructs in Fig 1B (S1A–S1C Fig).

**Fig 4:**
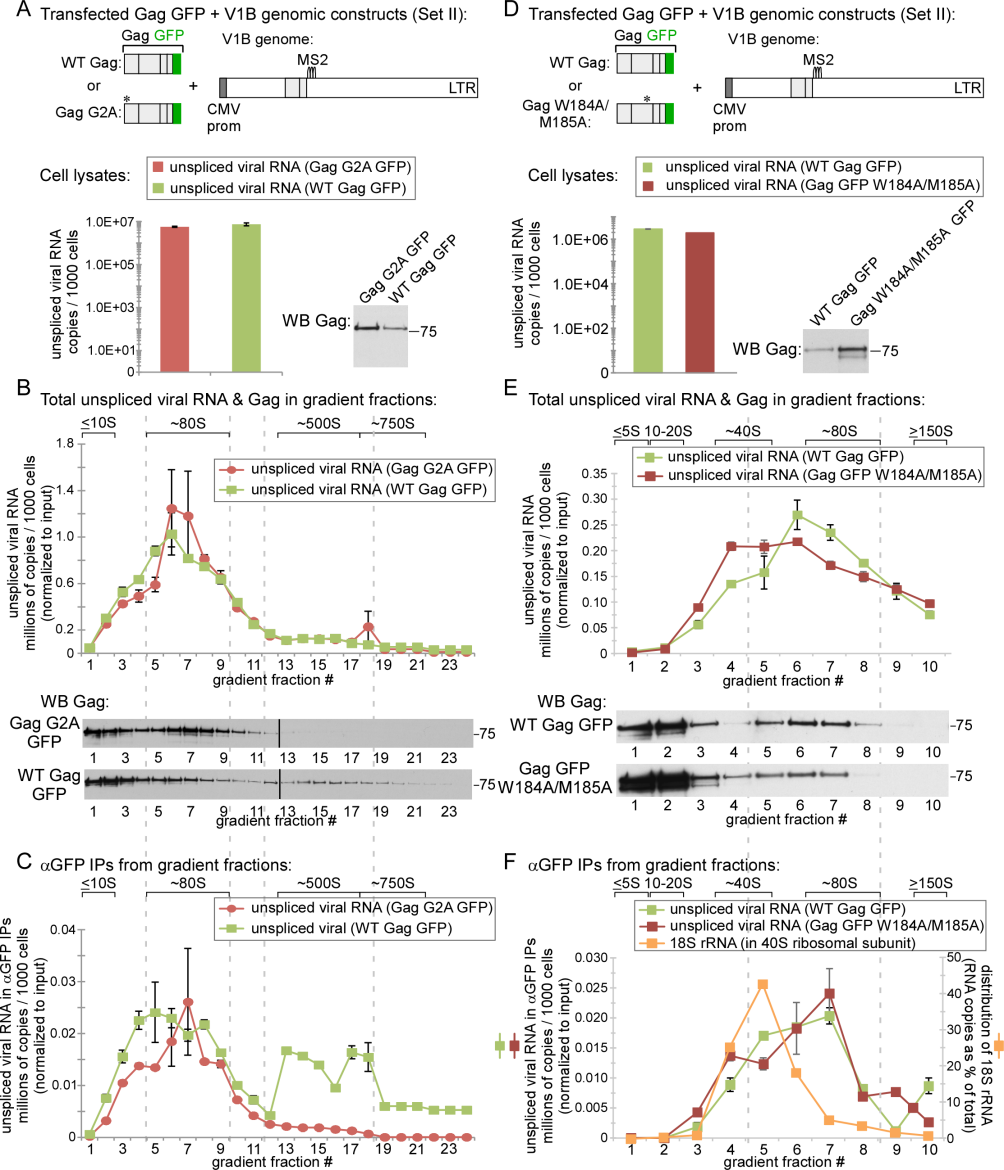
The ~80S assembly intermediate formed by WT Gag and two Gag mutants is the first intermediate to contain unspliced viral RNA. **(A)** COS-1 cells transfected with the indicated WT and Gag G2A constructs (Set II constructs in Fig 1A) were harvested following PuroHS treatment, and the number of unspliced viral RNA copies per 1000 cells was determined for cell lysates. (**B)** Lysates from A were analyzed by velocity sedimentation, and the number of unspliced viral RNA copies per 1000 cells was determined for each gradient fraction and normalized to inputs in A. (**C)** Gradient fractions from B were subjected to IP with αGFP, and the number of unspliced HIV-1 RNA copies per 1000 cells was determined for IP eluates from each gradient fraction and normalized to inputs in A. (**D)** COS-1 cells transfected with the indicated Gag W184A/M185A construct (Set II construct in Fig 1A) were harvested following PuroHS treatment, and the number of unspliced viral RNA copies per 1000 cells was determined for cell lysate. (**E)** Lysate from D was also analyzed by velocity sedimentation, and the number of unspliced viral RNA copies per 1000 cells was determined for each gradient fraction and normalized to inputs in A. (**F)** Gradient fractions from E were subjected to IP with αGFP and the number of unspliced viral RNA copies per 1000 cells was determined for IP eluates from each gradient fraction and normalized to inputs in A, with units indicated on left side Y-axis. Gradient fractions were also directly analyzed for 18S rRNA, to mark the position of the 40S small ribosomal subunit. Quantity of 18S rRNA in gradient fractions is expressed as a distribution (% of RNA in all fractions), with units indicated on the right side Y-axis. Positions of kD markers are shown to the right of blots. Brackets at top show S value markers, and dotted lines demarcate assembly intermediates based on their migrations in the corresponding Gag WB. Error bars show SEM from duplicate samples. Data in each column are from a single experiment that is representative of three independent replicate experiments.

To identify complexes that contain WT Gag in association with unspliced viral RNA, we analyzed cell lysates co-expressing either GFP-tagged WT Gag or Gag G2A along with the V1B genomic construct. To allow a direct comparison with experiments in Fig 3, we treated lysates with PuroHS to disrupt monosomes and polysomes. In this experiment, unlike in Fig 3 experiments, we used a velocity sedimentation gradient that separately resolves complexes of ~80S, ~500S, and ~750S, which are formed by WT Gag (reviewed in [14]**)**. Unlike the gradients shown in Fig 3, the gradients used here do not resolve soluble proteins and small complexes well, since complexes ranging from ~10S to ~80S are distributed across only a few fractions. Levels of intracellular Gag protein were similar at steady state for both WT Gag and Gag G2A, as were levels of unspliced viral RNA (Fig 4A). Gag G2A protein formed only the ~10S and ~80S intermediates (as observed in Fig 3B); in contrast, at steady state, WT Gag protein formed both of these intermediates as well as the expected ~500S late assembly intermediate and ~750S completed immature capsid (Fig 4B, WB), as shown previously [15,19,20]. Notably, in the WT Gag and Gag G2A lysates, nearly all the unspliced viral RNA was in the ~80S assembly intermediate (Fig 4B, graph). Additionally, IP with αGFP revealed that both WT and Gag G2A were associated with unspliced viral RNA in the ~80S assembly intermediate (Fig 4C, graph). Moreover, IP with αGFP showed that WT Gag was also associated with unspliced viral RNA in ~500S and ~750S complexes (Fig 4C, graph), both of which are formed by WT Gag but not targeting-defective Gag G2A, and likely represent the ~500S assembly intermediate and the ~750S completed capsid, respectively. Most importantly, although WT Gag forms a prominent ~10S assembly intermediate, αGFP IP of this complex revealed no unspliced viral RNA associated with this intermediate (Fig 4C; 0.4 copies of unspliced viral RNA per cell in the fraction 1 IP), as was the case for Gag G2A (Figs 3C and 4C). Note that since our lysates are loaded with sample at the top of the gradients, this very low value includes potential contamination from the top-loaded lysate. We also observed that the amount of WT Gag in the ~80S region is smaller following PuroHS treatment than in previous studies in which we did not treat with PuroHS [19,20,22]. This suggested that PuroHS, while having the advantage of disrupting translating complexes, may also cause some disruption of the ~80S assembly intermediate, leading to Gag-containing complexes that peak in the ~80S region but are broader, and less uniform in size and composition, than in our previous studies. Thus, for technical reasons the size of the ~80S assembly intermediate appears to be more approximate here than in our previous studies. Given this caveat (which is addressed by harvest in the absence of PuroHS below), our data are consistent with the ~80S assembly intermediate being the smallest assembly intermediate that is detected at steady state and contains WT Gag associated with unspliced HIV-1 RNA. Additionally, we found that WT Gag is also associated with unspliced HIV-1 RNA in a ~500S late assembly intermediate and the fully assembled ~750S completed immature capsid (Fig 4C), as would be expected.

Thus far in this study, we had shown that 1) both Gag G2A and WT Gag associate with unspliced viral RNA in an ~80S complex (Figs 3C and 4C); 2) that this ~80S complex likely contains the RNA granule protein and assembly facilitator DDX6 (Fig 3F) and therefore likely corresponds to the previously described RNA-granule-derived ~80S capsid assembly intermediate; and 3) as expected, WT Gag is associated with unspliced viral RNA in the 80S assembly intermediate as well as the ~500S assembly intermediate and the fully assembled ~750S completed capsid (Fig 4C). If the ~80S and ~500S complexes that contain WT Gag associated with unspliced viral RNA correspond to assembly intermediates, we would also expect unspliced viral RNA in these complexes to be associated with another host protein marker of assembly intermediates, the cellular facilitator of assembly ABCE1 [19,20,22], even after PuroHS treatment. Our previous data suggested that ABCE1 is also found in a subclass of small host RNA granules that are co-opted by HIV-1 [22] and in ~80S and ~500S assembly intermediates that are derived from co-opted host RNA granules and contain Gag in association with ABCE1 by coIP [15,19,21,22]. Additionally, we had shown that unspliced viral RNA is associated with ABCE1 in ~80S/150S and ~500S assembly intermediates by αABCE1 IP of lysates that were not treated with PuroHS [19]. However, because ABCE1 is known to be critical for translation termination (reviewed in [50]), here we asked whether ABCE1 is associated with unspliced viral RNA following treatment of lysates with PuroHS, which fully disassembles polysomes and monosomes (Fig 2). Indeed, analysis of PuroHS-treated lysates from cells expressing WT Gag GFP and the V1B genomic construct *in trans* revealed that αABCE1 immunoprecipitated unspliced viral RNA from the ~80S/150S and ~500S regions of the gradient (S2A–S2C Fig), which are known to contain the ~80S and ~500S assembly intermediates. Thus, PuroHS treatment allows us to conclude that the ~80S and ~500S complexes containing ABCE1 associated with unspliced HIV-1 RNA are not monosomes and polysomes, and instead likely represent ABCE1-containing assembly intermediates. However, a disadvantage of using PuroHS is that the high salt concentration appears to cause broader peaks due to partial disruption of assembly intermediates. Such partial disruption of the ~80S Gag-containing complex could explain why only a small amount of Gag is observed in the ~80S region following PuroHS treatment, compared to the large and distinct ~80S Gag peak observed in earlier studies where PuroHS was not used [19,20,22]. We also observed that the peak of unspliced HIV-1 RNA immunoprecipitated by αABCE1 lies slightly to the right of the main ~80S Gag peak (compare S2C Fig, graph to S2B Fig, WB). This could be due to heterogeneity of complexes in this region or variability in the accessibility of epitopes in this region. While these migration issues will need to be explored further in the future, the data suggest that unspliced viral RNA is associated with both WT Gag (Fig 4C) and ABCE1 (S2C Fig) in the ~80S/150S and the ~500S fractions.

### Additional approaches aimed at finding a soluble complex that contains Gag associated with unspliced HIV-1 RNA

So far, our data supported a model in which HIV-1 Gag first associates with HIV-1 RNA within RNA granule-derived complexes found in the ~80S and ~500S regions of the gradient (Model 2). Moreover, while we repeatedly identified a large pool of soluble Gag, we did not find it associated with unspliced HIV-1 RNA, arguing against a model in which soluble ~10S Gag is associated with unspliced HIV-1 RNA (Model 1). However, it is possible that our approaches prevented us from detecting a soluble complex containing Gag associated with unspliced HIV-1 RNA. Approaches that could have been problematic include 1) our use of only the Gag G2A mutant, rather than other mutants, to trap a small Gag-containing complex associated with unspliced viral RNA, 2) transfection of non-human primate COS-1 cells, which we used to minimize problems resulting from endocytosis of released virus as previously described [19], and 3) treatment with PuroHS to eliminate translating complexes from our analyses. We addressed the first of these concerns by examining a second assembly-defective Gag construct that associates with intracellular unspliced viral RNA. For this purpose, we analyzed the W184A/M185A Gag mutant, which fails to complete multimerization, but targets to the PM [18,19,49,51–54] unlike the Gag G2A mutant. The W184/M185 residues are known to be important for interhexameric CA-CA dimer interface contacts, which are necessary for subsequent steps in assembly [19,52–54]. We had previously shown that when expressed from an HIV-1 provirus, Gag W184A/M185A is arrested in the form of an ~80S intermediate that is associated with the PM [19]. Here we analyzed PuroHS treated lysates of COS-1 cells transfected with Gag GFP W184A/M185A and the V1B genomic constructs *in trans* (Set II constructs in Fig 1A; also in S3A Fig). As expected, under these conditions Gag W184A/M185A is assembly-defective but associates with intracellular unspliced viral RNA (S3B and S3C Fig). We also confirmed that Gag W184A/M185A is arrested as an ~80S assembly intermediate (S3D Fig). We then used RT-qPCR to identify all complexes in lysates that contain unspliced viral RNA, and the subset of those complexes containing Gag W184A/M185A in association with unspliced viral RNA by αGFP coIP (Fig 4D–4F). To maximize our ability to detect small complexes, we used a velocity sedimentation gradient that provided high resolution in the ~10S - 150S range (similar to Fig 2B–2E, Fig 3B and 3C and Fig 3E and 3F; and unlike Fig 4B and 4C). We observed essentially no unspliced viral RNA in fractions <30S; instead all unspliced viral RNA was found in fractions ≥30S (Fig 4E). Moreover, αGFP IP of gradient fractions revealed that Gag GFP W184A/M185A was associated with unspliced viral RNA in a complex that peaks at ~80S and likely corresponds to the ~80S assembly intermediate, as observed for WT Gag GFP analyzed in parallel (Fig 4F; also compare to Fig 4C). Note that both the complex containing WT Gag associated with unpsliced viral RNA and the complex containing Gag WM184A/M185A associated with unspliced viral RNA fit the definition of ~80S complexes given that both clearly peak in the 80S region in Fig 4F. However, since these peaks are relatively wide and could therefore include smaller complexes within them, we also quantified 18S rRNA, which is found in the 40S small ribosomal subunit, to define the smallest complex that could be hidden within this peak. We found that the 40S marker is first found in fraction 4 and peaks in fraction 5 (Fig 4F); thus, the smallest complex that could be associated with unspliced viral RNA but hidden within the broad ~80S peak is ≥~30-40S. Given that the 40S ribosomal subunit contains one RNA and 33 cellular proteins, complexes in this size range are likely to be ribonucleoprotein complexes that contain numerous host proteins rather than a solely a dimer of Gag associated with unspliced viral RNA. From this analysis, which maximizes resolution of soluble complexes present at steady state, we conclude that most likely the smallest complex containing unspliced viral RNA in association with WT Gag or assembly-defective viral-RNA-binding Gag mutants (Gag G2A and Gag W184A/M185A) is either the ~80S assembly intermediate or a complex of ~30-40S, at the smallest, if a buried peak is present. Although we cannot exclude the possibility that a buried complex of ~30-40S is present, we favor the hypothesis that the broad ~80S peak represents an ~80S complex containing Gag associated with unspliced viral RNA that has been partially disrupted due to exposure to high salt during PuroHS treatment; this possibility is further supported by experiments described below. Notably, once again we did not detect a discrete ~10-20S peak of Gag associated with unspliced HIV-1 RNA, arguing against soluble Gag being associated with unspliced HIV-1 RNA to the limit of our detection.

**Fig 5:**
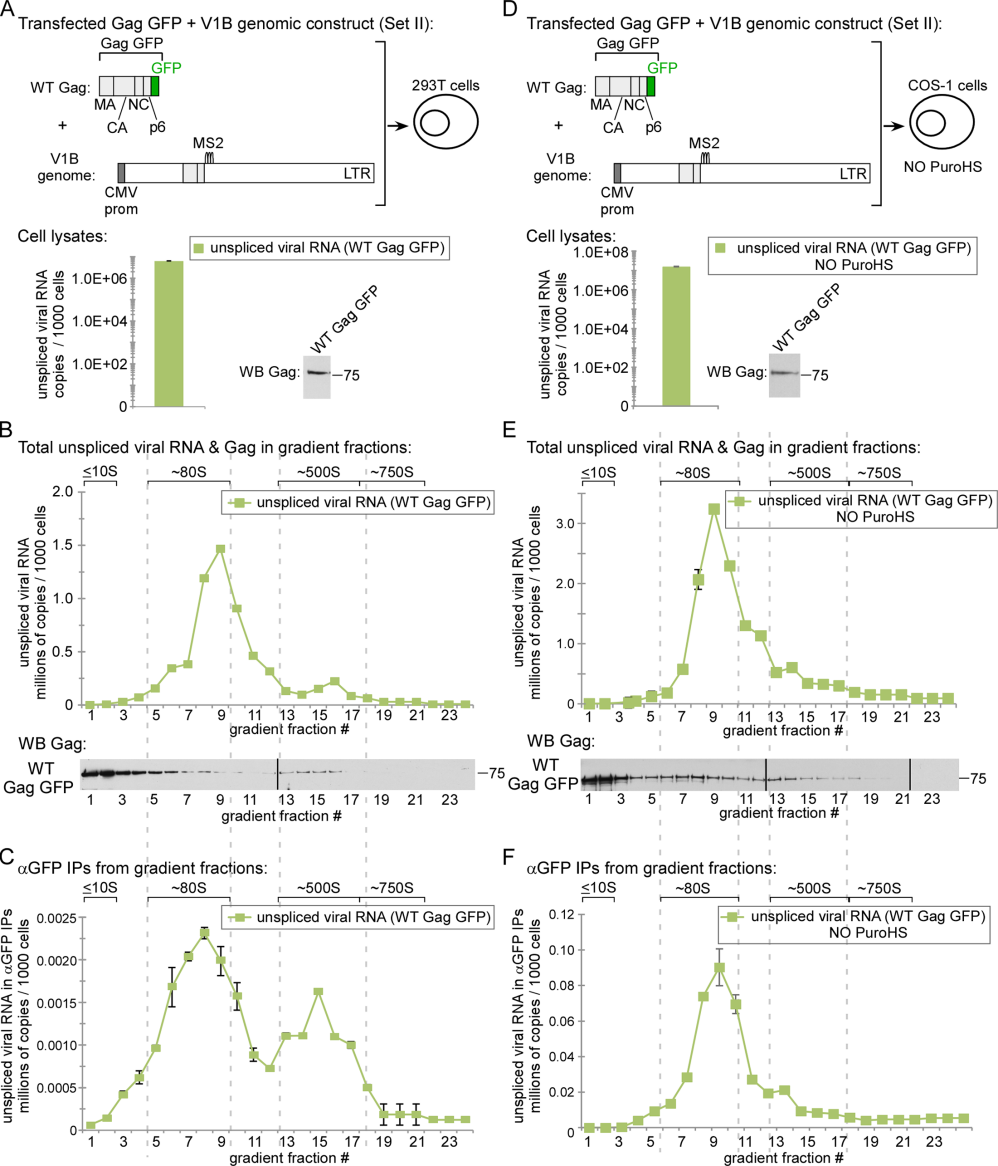
The ~80S assembly intermediate formed by WT Gag is the first assembly intermediate to contain unspliced viral RNA in human 293T cells and in the absence of PuroHS treatment. **(A)** 293T cells transfected with the indicated construct (Set II construct in Fig 1A) were harvested following PuroHS treatment, and the number of unspliced viral RNA copies per 1000 cells was determined for cell lysate. (**B)** Lysate from A was analyzed by velocity sedimentation, and the number of unspliced viral RNA copies per 1000 cells was determined for each gradient fraction. (**C)** Gradient fractions from B were subjected to IP with αGFP, and the number of unspliced viral RNA copies per 1000 cells was determined for IP eluates from each gradient fraction. **(D)** COS-1 cells transfected to express WT Gag GFP and the V1B genome (Set II constructs in Fig 1A) were harvested under standard conditions without PuroHS treatment, and the number of unspliced viral RNA copies per 1000 cells was determined for cell lysates. (**E)** Lysate from D was analyzed by velocity sedimentation, and the number of unspliced HIV-1 RNA copies in the equivalent of 1000 cells was determined in each gradient fraction. **(F)** Gradient fractions from E were subjected to IP with αGFP, and the number of unspliced HIV-1 RNA copies in the equivalent of 1000 cells was determined for IP eluates from each fraction. Positions of kD markers are shown to the right of blots. Brackets at top show S value markers, and dotted lines demarcate assembly intermediates based on their migrations in the corresponding Gag WB. Error bars show SEM from duplicate samples. Data in each column are from a single experiment that is representative of two independent replicate experiments.

To address the possibility that use of non-human primate COS-1 cells prevented us from identifying smaller complexes containing Gag-associated with unspliced viral RNA, we repeated our analyses in human 293T cells that were transfected to express WT Gag GFP and the V1B genomic construct (Fig 5A) and were harvested, using PuroHS treatment of lysates, at an early time point to avoid virus endocytosis. We observed that the amount of Gag in the ~80S peak by WB following harvest with PuroHS treatment was smaller than in a previous analysis of 293T cells that were harvested without PuroHS treatment (compare Fig 5B WB in the current study to Fig 11B WB in [20]). We hypothesize that this is because high salt partially disrupts the ~80S assembly intermediate, as described above. Despite the possible partial disruption of the ~80S intermediate, αGFP IP revealed that WT Gag is associated with unspliced viral RNA in an ~80S complex that likely corresponds to the ~80S assembly intermediate in 293T cells (Fig 5C); additionally, Gag in the ~500S assembly intermediate from these cells is also associated with unspliced viral RNA (Fig 5C). Notably, the large amount of soluble Gag observed in ~10S fractions of 293T lysates (Fig 5B, WB) was not associated with unspliced viral RNA by αGFP IP (Fig 5C). Two other observations are worthy of comment. First, once again the ~80S complex that contains Gag associated with unspliced HIV-1 RNA is a relatively wide complex (Fig 5C); this is consistent with the disruptive effect of PuroHS, especially given results described below. However, further studies will be needed to show this definitively. Second, a ~750S completed capsid was not observed in 293T lysates by WB or by αGFP IP of unspliced viral RNA unlike in COS-1 cells (compare Fig 5C to Fig 4C). This was not surprising since completed immature capsids undergo rapid budding and release from 293T cells and therefore are typically not observed in cell lysates, especially when harvested at early time points (see Fig 10 in [19]). Overall, the results we obtained in 293T cells were similar to those obtained in COS-1 cells (compare Fig 5B and 5C to WT Gag in Fig 4B and 4C).

Additionally, we addressed the possibility that PuroHS treatment of cell lysates could lead to mis-identification of the earliest assembly intermediate containing Gag associated with unspliced viral RNA, for example by causing broadening of the ~80S peak with masking of a complex smaller than ~80S that contains Gag associated with unspliced viral RNA (as alluded to in the description of Fig 4F above). Cells transfected to express WT Gag GFP and the V1B genomic construct *in trans* (Fig 5D) were harvested without PuroHS treatment, leaving monosomes and polysomes intact as shown above (Fig 2B–2E, open symbols). Analysis by velocity sedimentation once again demonstrated that almost no unspliced viral RNA was present in the <40S fractions (Fig 5E), and WT Gag was only associated with unspliced viral RNA by αGFP IP in a large peak that corresponds to the ~80S assembly intermediate and in a small peak that likely represents the ~500S late assembly intermediate (fractions 12–14, Fig 5F). Note that when Gag GFP is expressed with the V1B genomic construct *in trans*, αGFP would be expected to immunoprecipitate both translating Gag GFP associated with Gag GFP mRNA and non-translating Gag GFP associated with unspliced V1B RNA; however RT-qPCR analysis of αGFP IP samples should not detect the translating complexes since our qPCR oligos only detect V1B genomic RNA and not the mRNA from Gag GFP supplied *in trans*. Therefore, the ~80S complex observed in Fig 5F does not represent monosomes, and should instead represent a non-translating complex containing assembling WT Gag GFP associated with unspliced HIV-1 RNA. Importantly, in this experiment, the ~80S assembly intermediate that was observed in the absence of PuroHS, and contains Gag associated with nontranslating unspliced viral RNA Gag, is defined by a much narrower and more homogenous peak than the corresponding complex detected after PuroHS treatment of comparable lysates (compare ~80S complexes in Fig 5F vs. Fig 4C). This is consistent with the hypothesis that PuroHS treatment causes the ~80S assembly intermediate to partially dissociate. Thus, our studies of WT Gag expressed with the V1B genome *in trans* harvested with ribosomes intact once again identify the ~80S assembly intermediate as the smallest complex containing Gag and unspliced viral RNA; moreover, these data support a model in which the smallest complex that contains Gag associated with unspliced viral RNA is an assembly intermediate of relatively uniform ~80S size when cells are harvested in a more physiological buffer without high salt treatment.

### Unspliced HIV-1 RNA is associated with ABCE1 in RNA granule-derived ~80S and ~500S assembly intermediates from chronically infected human T cells

**Fig 6:**
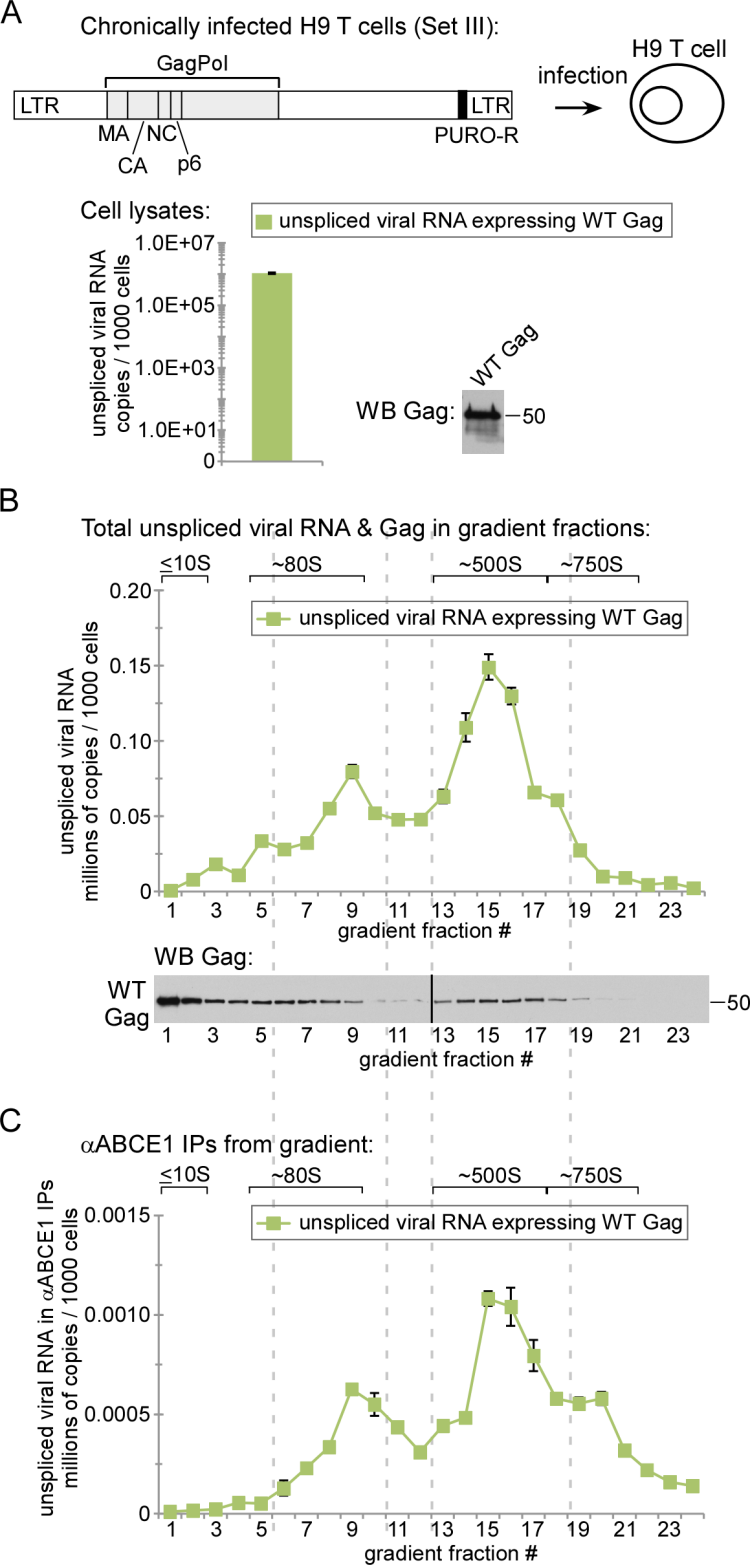
Analysis of unspliced viral RNA in the ~80S and ~500S assembly intermediates from chronically infected H9 T cells. **(A)** Human H9 T cells chronically infected with the indicated provirus (Set III construct in Fig 1A) were harvested following PuroHS treatment, and the number of unspliced HIV-1 RNA copies per 1000 cells was determined for cell lysates. **(B)** Lysate from A was also analyzed by velocity sedimentation, and the number of unspliced HIV-1 RNA copies per 1000 cells was determined for each gradient fraction. (**C)** Gradient fractions from B were subjected to IP with αABCE1 and the number of unspliced HIV-1 RNA copies per 1000 cells was determined for IP eluates from each gradient fraction. Positions of kD markers are shown to the right of blots. Brackets at the top show S value markers, and dotted lines demarcate assembly intermediates based on their migrations in the corresponding Gag WB. Error bars show SEM from duplicate samples. Data in each column are from a single experiment that is representative of three independent replicate experiments.

Previously, we showed by coIP that, in human H9 T cells chronically infected with HIV, Gag associates with ABCE1 and DDX6 in complexes that correspond to the ~80S and ~500S assembly intermediates [22]. Here we analyzed lysates of these cells to determine whether the ~80S intermediate is the smallest assembly intermediate that contains HIV-1 Gag associated with unspliced HIV-1 RNA in HIV-1-infected human T cells. PuroHS treatment was used in these analyses because these cells express proviruses (with Gag produced *in cis*); thus, in these cells, unspliced HIV-1 RNA would be found in both non-translating and translating complexes that contain Gag, and PuroHS would allow us to examine only the non-translating complexes. Because these chronically infected cells express native Gag, not Gag GFP, we did not immunoprecipitate with αGFP; nor did we immunoprecipitate with αGag since Gag epitopes are masked during multimerization, as described above. Instead, these IP analyses were performed with αABCE1, which we had shown to be effective in immunoprecipitating unspliced viral RNA in the ~80S and ~500S assembly intermediates (S2C Fig). Our analyses revealed that, in chronically infected H9 cells, the vast majority of the non-nuclear non-translating unspliced HIV-1 RNA is in ~80S and ~500S complexes, and unspliced HIV-1 RNA in those complexes is associated with ABCE1 by IP (Fig 6A–6C). Gag was also present in the ~80S and ~500S regions of the gradient by WB (Fig 6B), as shown previously (Fig 3 in [22]); moreover those previous data had demonstrated that αABCE1 coimmunoprecipitated Gag in ~80S and ~500S assembly intermediates from chronically infected H9 T cells (Fig 3 in [22]). Thus, these data suggest that, in chronically infected human T cells, ABCE1 in ~80S and ~500S assembly intermediates is associated with unspliced HIV-1 RNA.

Two additional observations are of interest in the analysis of chronically infected human T cells. First, some unspliced HIV-1 RNA was observed in fractions 2–5 of this gradient (Fig 6B); however, additional experiments suggested that the unspliced HIV-1 RNA in these fractions was not associated with Gag (S4 Fig). In those experiments, lysates from chronically infected H9 T cells were subjected to IP with αGag, which detects Gag multimers poorly as noted above, but detects soluble Gag and small Gag oligomers effectively. In gradients of infected T cells, αGag immunoprecipitated fewer than 1 copy of unspliced HIV-1 RNA per cell from the ~10-40S region and immunoprecipitated 100-fold more copies of unspliced HIV-1 RNA from both the ~80S region and the ~500S region (S4 Fig). Thus, these additional experiments suggest that unspliced HIV-1 RNA in the first few fractions of Fig 6B is unlikely to be in small Gag-associated complexes, and is more likely to have resulted from high-salt-mediated disruption of ~80S assembly intermediates. Separate from that issue, it was interesting that at steady state in chronically infected H9 T cells, the ratio of non-translating unspliced HIV-1 RNA in 80S vs. 500S complexes, as determined by RNA peak height, was shifted towards the ~500S peak, compared to transfected COS-1 or 293T cells (e.g. compare ~80S to ~500S RNA peak height in Fig 6B vs Figs 4B and 5B). Such variability in the ~500S to ~80S ratio could reflect kinetic differences in efficiency of assembly or budding in different cell types and/or between experiments. For example, human T cells could form the ~500S assembly intermediate more efficiently or could undergo virus budding less efficiently; alternatively there could be experiment-to-experiment differences in formation of these transient, highly dynamic assembly intermediates and in budding kinetics. While further studies of these cells will be needed, our initial studies of these chronically infected H9 T cells support a model in which the majority of HIV-1 RNA in HIV-1 infected human T cells is present in high molecular weight complexes.

### ΔΨ RNA is suboptimal for testing the RNA granule model because it displays only a modest packaging defect

To generate an RNA that is packaging defective, others have utilized a genomic construct in which stem loops 3 and 4 of the RNA packaging element psi (Ψ; which contains four stem loops), are deleted (ΔΨ; [12,13]). Previous studies in 293T cells revealed only a 3–4 fold reduction in the intracellular association of WT Gag GFP with unspliced ΔΨ V1B viral RNA, compared to WT unspliced viral RNA; the overlapping error bars in these data emphasize the modest nature of the defect (Fig 1 in [13]). If the ~80S complex is the first assembly intermediate in which Gag associates with unspliced HIV-1 RNA, we would predict that cells expressing WT Gag and an unspliced HIV-1 RNA that is profoundly packaging-defective would generate an ~80S assembly intermediate that contains WT Gag but little or no packaging-incompetent unspliced HIV-1 RNA. However, if the packaging defect of a co-transfected HIV-1 RNA is minimal, a less dramatic (and possibly insignificant) defect in association of ~80S Gag and unspliced HIV-1 RNA would be expected, making such a construct less useful for testing the RNA granule model of packaging. Therefore, we first sought to define the magnitude of the packaging defect of the ΔΨ V1B genomic construct. Cells were co-transfected to express WT Gag GFP and either the ΔΨ or WT V1B genome (Set II constructs in Fig 1A). Cell lysates and VLPs were analyzed by WB for Gag, and by RT-qPCR for unspliced V1B viral RNA (S5A Fig). Intracellular WT Gag GFP and intracellular unspliced viral RNA were expressed to similar steady state levels for both groups, but the cells expressing ΔΨ viral RNA produced somewhat fewer VLPs by Gag WB in two independent experiments. When the results of these two experiments were averaged and normalized to intracellular unspliced viral RNA levels, we observed a reduction in ΔΨ unspliced viral RNA in VLPs to 29% relative to WT RNA in VLPs analyzed in parallel (S5A Fig, Efficiency of ΔΨ packaging graph). When the results were normalized to both intracellular unspliced viral RNA levels and VLP Gag levels, a reduction in ΔΨ unspliced viral RNA in VLPs to 46% was observed. This two- to three-fold defect in packaging of ΔΨ observed in our VLP analyses is consistent with the similar modest defect observed by others for WT Gag association with intracellular ΔΨ unspliced viral RNA [13]. Since our analyses detected >20,000 copies of unspliced HIV-1 RNA in the ~80S peak per 1000 cells (Fig 4C), a two- to three-fold reduction in this peak is unlikely to display the significance needed to unambiguously detect a reduction in candidate packaging complex formation by the ΔΨ construct. For this reason, we concluded that the ΔΨ3/4 packaging defect is too modest to be useful in testing the RNA granule model of packaging; instead, we opted to analyze a Gag construct that displays more impressive packaging defects.

### Gag requires a viral-RNA-binding domain to associate with the subset of RNA granules containing unspliced HIV-1 RNA

In contrast to the modest VLP packaging defect displayed by ΔΨ viral RNA (S5A Fig), the assembly-competent Gag Zip construct releases VLPs that closely resemble WT VLPs morphologically [18,32,34,55] but display a profound defect in HIV-1 RNA packaging, as observed by others [34] and by us (Fig 1C). Therefore this GagZip construct, which contains LZ in place of NC, provides a statistically significant packaging defect that should allow us to test whether HIV-1 Gag has evolved one or more mechanisms for targeting to RNA granules that contain unspliced HIV-1 RNA. Given the longstanding observation that the NC domain is required for association of Gag with unspliced HIV-1 RNA, we hypothesized that NC would be critical for targeting Gag to the subset of RNA granules that contains unspliced HIV-1 RNA. Additionally, other domains could also be involved in localizing Gag to a broader class of RNA granules, as suggested by our earlier studies of Gag Zip [22]. Previously, Gag Zip was used to demonstrate that NC has two functions during immature capsid assembly—NC binds specifically to unspliced HIV-1 RNA and also promotes oligomerization of Gag via non-specific RNA association [33,34]. Because LZ promotes direct protein-protein interactions, it substitutes for the oligomerization function of NC; thus, Gag Zip is assembly-competent and produces VLPs [18,34,56]. However, because LZ does not bind to RNA, these Gag Zip VLPs lack viral RNA or other RNA [34,56]. Interestingly, we found previously that, despite its inability to interact with RNA (Fig 1C), Gag Zip forms the ~80S and ~500S ABCE1- and DDX6-containing assembly intermediates [18,22]. These data raised the possibility that Gag Zip contains a determinant that allows it to localize to a broad class of ABCE1- and DDX6-containing RNA granules, but not to the subset of these granules that contains unspliced viral RNA because it lacks the viral-RNA-binding NC domain.

**Fig 7:**
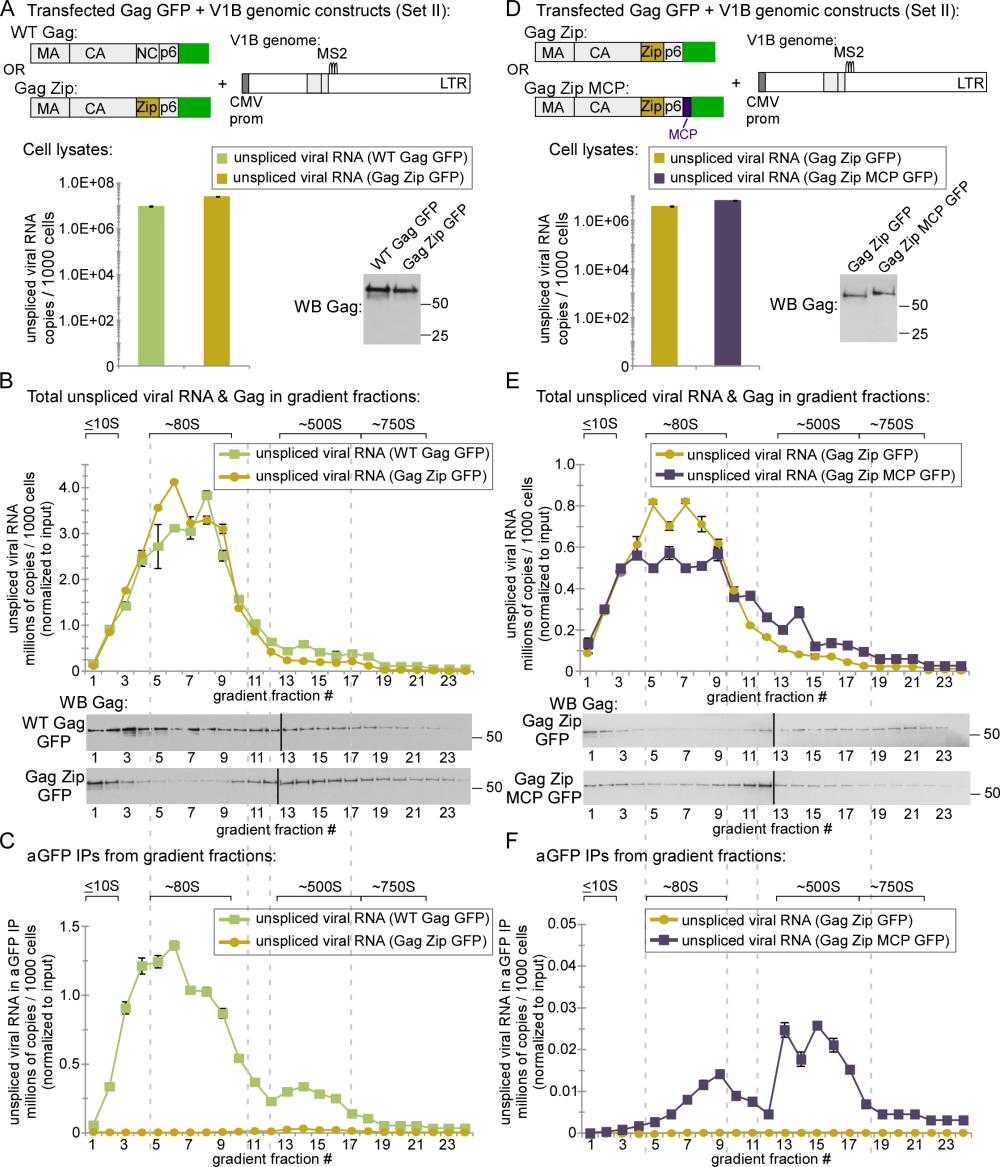
Gag Zip fails to associate with viral-RNA-containing granules, but is rescued by a heterologous viral-RNA-binding domain. **(A)** COS-1 cells transfected with indicated constructs (Set II constructs in Fig 1A) were harvested following PuroHS treatment, and the number of unspliced viral RNA copies per 1000 cells was determined for cell lysates. (**B)** Lysates from A were analyzed by velocity sedimentation, and the number of unspliced HIV-1 RNA copies per 1000 cells was determined for each gradient fraction and normalized to inputs in A. (**C)** Gradient fractions from B were subjected to IP with αGFP, and the number of unspliced viral RNA copies per 1000 cells was determined was determined for IP eluates from each gradient fraction and normalized to input in A. (**D)** COS-1 cells transfected with indicated constructs (Set II constructs in Fig 1A) were harvested following PuroHS treatment, and the number of unspliced viral RNA copies per 1000 cells was determined for cell lysates. (**E)** Lysates from D were analyzed by velocity sedimentation, and the number of unspliced viral RNA copies per 1000 cells was determined for each gradient fraction and normalized to inputs in A. (**F)** Gradient fractions from E were subjected to IP with αGFP, and the number of unspliced viral RNA copies per 1000 cells was determined for IP eluates from each gradient fraction and normalized to inputs in A. Positions of kD markers are shown to the right of blots. Brackets at top show S value markers, and dotted lines demarcate assembly intermediates based on their migrations in the corresponding Gag WB. Error bars show SEM from duplicate samples. Data in each column are from a single experiment that is representative of two independent replicate experiments.

Before testing this hypothesis, we first confirmed that Gag Zip GFP, like Gag Zip, produces VLPs that lack unspliced viral RNA when co-transfected with the V1B plasmid *in trans* (S5B Fig). We next analyzed PuroHS-treated lysates of cells transfected with WT Gag or Gag Zip GFP, and the V1B genomic construct *in trans* (Set II constructs in Fig 1A). Both Gag proteins were expressed at similar steady state levels, as was the V1B RNA (Fig 7A), and unspliced viral RNA was primarily in an ~80S complex in both cases (Fig 7B graph). WB confirmed that Gag Zip GFP forms a ~500S complex (Fig 7B WB), consistent with the previously described ~500S Gag Zip assembly intermediate [18]. In addition, previously we confirmed that Gag Zip also forms the ~80S assembly intermediate, albeit at lower levels than for WT Gag as observed previously (see dark exposures of Fig 4 and Fig 5 in [18]). Notably, αGFP coimmunoprecipitated unspliced viral RNA from ~80S and ~500S fractions of cells expressing WT Gag GFP, but failed to coIP unspliced viral RNA from any fraction of the Gag Zip GFP gradient (Fig 7C, compare to WT in Fig 4C). Controls using IP followed by WB showed that αGFP immunoprecipitated Gag Zip GFP protein as effectively as WT Gag GFP protein from ~80S and ~500S fractions (S5C Fig), so the failure to coimmunoprecipitate unspliced viral RNA with Gag Zip GFP cannot be attributed to reduced αGFP IP efficiency. These data argue that both WT Gag and Gag Zip localize to ~80S RNA granules to form ~80S assembly intermediates, but WT Gag stably associates with a subset of these RNA granules that contains unspliced viral RNA, while Gag Zip does not stably associate with this unspliced-viral-RNA-containing subset even though it associates with a related but broader class of RNA granules. From these data, we conclude that while GagZip is capable of forming an RNA-granule-derived ~80S assembly intermediate, in the absence of NC this ~80S assembly intermediate will contain Gag but not unspliced HIV-1 RNA. Thus, these data suggest that NC directs Gag to the subset of the granules that contains unspliced viral RNA. Importantly, our data also suggest that a region of Gag outside of NC (present in Gag Zip but not in MACA) is responsible for bringing Gag to a broader class of RNA granules, most of which lack viral RNA.

Finally, we asked whether a heterologous domain that confers binding to unspliced viral RNA (here termed a heterologous viral-RNA-binding domain) is sufficient to restore targeting of Gag Zip to the subset of the granules that contains unspliced viral RNA. Because the V1B genomic RNA contains binding sites for the bacteriophage MS2 (referred to as MS2 stem loops), we reasoned that the MS2 coat protein (MCP), which binds with high affinity to MS2 stem loops (reviewed in [57]), could serve as a heterologous viral-RNA-binding domain if inserted into Gag Zip. Therefore, we generated a construct, here called Gag Zip MCP, in which MCP is fused to the C-terminus of Gag Zip. Additionally, we showed that Gag Zip MCP forms VLPs that contain the V1B genome, like WT Gag and unlike Gag Zip (S5B Fig). When the VIB genomic construct was co-transfected either with Gag Zip or Gag Zip MCP to similar steady state levels (Fig 7D) and analyzed on gradients, unspliced viral RNA was found largely in the ~80S fractions in both cases (Fig 7E). However, αGFP coimmunoprecipitated unspliced viral RNA from the ~80S and ~500S assembly intermediates formed by Gag Zip MCP GFP, but not from the corresponding complexes formed by Gag Zip, analyzed in parallel (Fig 7F). Thus, fusion to MCP, a heterologous viral-RNA-binding domain, redirected Gag Zip to the specific subset of RNA granules that contains unspliced viral RNA, resulting in formation of ~80S and ~500S assembly intermediates containing unspliced viral RNA associated with Gag Zip MCP. In keeping with this, the MCP fusion also restored unspliced viral RNA in released VLPs (Fig 7F and S5B Fig). These data confirmed that either a native or heterologous viral-RNA-binding domain is required to target Gag to the subset of granules containing unspliced viral RNA.

### *In situ* studies confirm that assembling Gag colocalizes with RNA granule proteins DDX6 and ABCE1

**Fig 8:**
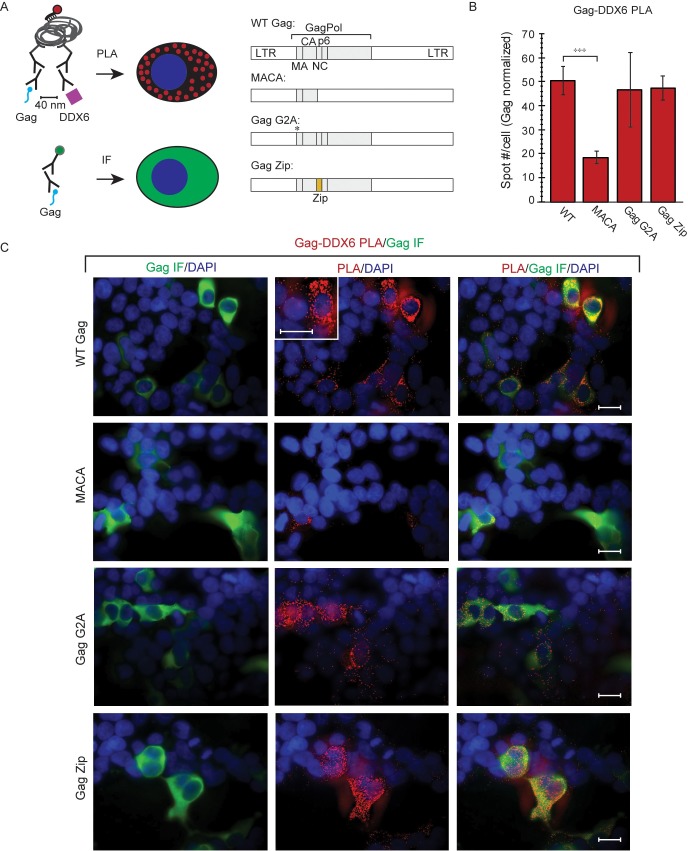
Gag-DDX6 colocalization *in situ* upon provirus expression. (**A)** PLA was used to detect regions in which Gag is within 40 nm from DDX6 *in situ*, with concurrent αGag IF for quantification of intracellular Gag levels. An experimental schematic for PLA methods is shown. For PLA experiments, 293T cells were transfected with the proviruses shown in diagram (Set 1 constructs in Fig 1A). **(B)** The average number of PLA spots per cell was determined for all Gag-positive cells in five randomly chosen fields, and normalized to Gag levels. **C)** Representative images. From left to right for each construct: Gag IF (green) with DAPI-stained nuclei (blue), Gag-DDX6 PLA signal (red) with DAPI-stained nuclei (blue), and a merge of all three. Merge demonstrates that PLA spots are mainly in Gag-expressing cells. Inset in PLA panel shows a high magnification view of the cell to the right of the inset. Scale bars, 5 μm. Data are representative of three independent replicate experiments. Error bars show SEM (n = 5 fields). +++ indicates a significant difference relative to WT (p≤0.001).

**Fig 9:**
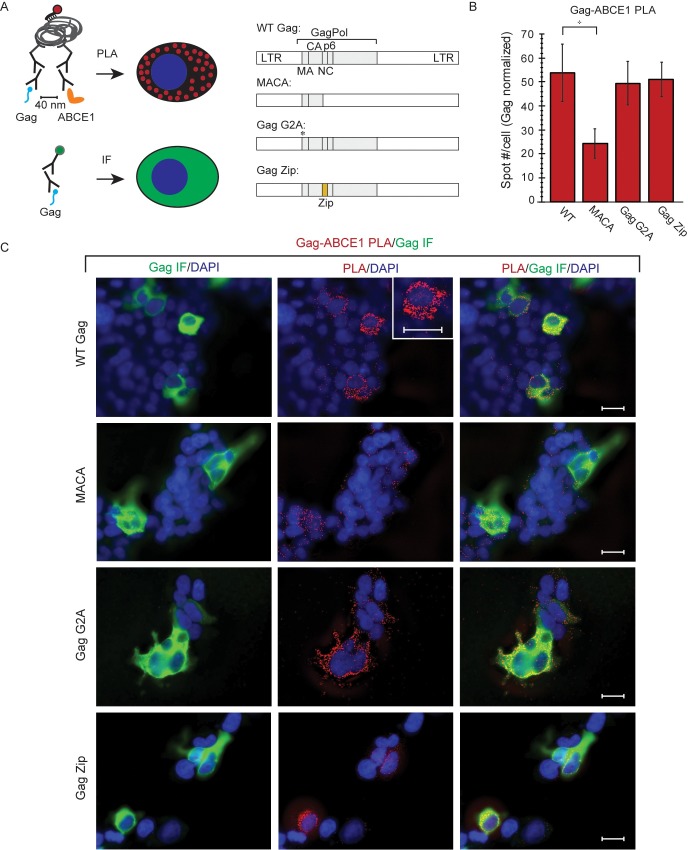
Gag-ABCE1 colocalization *in situ* upon provirus expression. (**A)** PLA was used to detect regions in which Gag is within 40 nm from ABCE1 *in situ*, with concurrent αGag IF for quantification of intracellular Gag levels. An experimental schematic for PLA methods is shown. For PLA experiments, 293T cells were transfected with the proviruses shown in diagram (Set 1 constructs in Fig 1A). **(B)** The average number of PLA spots per cell was determined for all Gag-positive cells in five randomly chosen fields, and normalized to Gag levels. **C)** Representative images. From left to right for each construct: Gag IF (green) with DAPI-stained nuclei (blue), Gag-ABCE1 PLA signal (red) with DAPI-stained nuclei (blue), and a merge of all three. Merge demonstrates that PLA spots are mainly in Gag-expressing cells. Inset in PLA panel shows a high magnification view of the cell to the left of the inset. Scale bars, 5 μm. Data are representative of three independent replicate experiments. Error bars show SEM (n = 5 fields). + indicates a significant difference relative to WT (p≤0.01).

Previously, to confirm coIP studies suggesting that assembling Gag associates with a host RNA granule, we had used *in situ* double labeling immunoelectron microscopy (IEM) to show colocalization of WT Gag (as well as some assembly-competent Gag mutants) with ABCE1, DDX6, and AGO2 (another RNA granule protein) at PM sites of assembly and budding in intact cells [15,19,20,22]. Here we asked whether we could use a second *in situ* technique to confirm that Gag associates with RNA granule proteins in intact cells. To do this, we sought to identify sites where Gag colocalizes with DDX6 or ABCE1 using the proximity ligation assay (PLA). PLA produces fluorescent spots at sites where two proteins are within 40 nm of each other *in situ*. Briefly, this method uses species-specific secondary antibodies conjugated to complementary oligonucleotide probes to detect primary antibodies bound to two proteins of interest; when a linker and other reagents are added, the probes on the secondary antibodies anneal if the two proteins of interest are ≤ 40 nm apart, thereby forming the template for a rolling circle amplification product that is recognized by a fluorophore-conjugated oligonucleotide [58](Fig 8A). We hypothesized that if assembling Gag associates with RNA granules containing ABCE1 and DDX6 as indicated by our biochemical studies, then the assembling Gag constructs (WT Gag, Gag G2A, and Gag Zip) should produce abundant Gag-DDX6 and Gag-ABCE1 PLA spots relative to assembly-incompetent MACA Gag, which fails to associate with granules (Fig 3A–3C) and does not coIP with DDX6 or ABCE1 [19,22]. To test this hypothesis, 293T cells were transfected with WT vs. mutant provirus (Set I constructs in Fig 1A) and analyzed for Gag-DDX6 or Gag-ABCE1 colocalization by PLA. Concurrent Gag IF allowed us to confirm that the vast majority of PLA spots were observed in Gag-expressing cells, and to choose fields for quantitation with comparable Gag levels. For cells expressing WT Gag, Gag G2A, or Gag Zip, these quantified fields contained ~50 Gag-DDX6 PLA spots per cell, three-fold more than for cells expressing MACA (Fig 8). Similar results were observed for Gag-ABCE1 PLA spots (Fig 9). Some PLA background was expected in cells transfected with MACA provirus, given that much of the non-nuclear DDX6 and ABCE1 is found in the soluble fraction of lysates (S5D Fig) and could therefore be in proximity outside of granules along with MACA, which is entirely localized to the soluble fraction (Figs 2C and 3B). As specificity controls for our PLA experiments, we also demonstrated that almost no DDX6 PLA signal was observed in Gag-expressing cells when either αGag or αDDX6 used for PLA was replaced with isotype-specific, non-immune control antibodies (S6A–S6C Fig, Negative controls 1 and 2 respectively). Additionally, mock-transfected cells displayed no PLA signal when assayed with Gag and DDX6 antibodies (S6C Fig). Similar results were obtained for ABCE1 negative controls but are not presented here. Thus, PLA appears to identify DDX6- and ABCE1-containing RNA granules that contain assembling Gag. Moreover, PLA confirms biochemical studies above, in which we showed that WT Gag and Gag G2A target to ABCE1- and DDX6-containing RNA granules (Fig 3F, Fig 6C, and S2C Fig), as well as previous quantitative IEM studies showing the colocalization of ABCE1 and DDX6 with assembling Gag proteins [15,19,20,22].

### Sites of Gag-DDX6 interaction *in situ* are far more numerous than P bodies and likely correspond to small, low intensity foci observed by DDX6 IF

**Fig 10:**
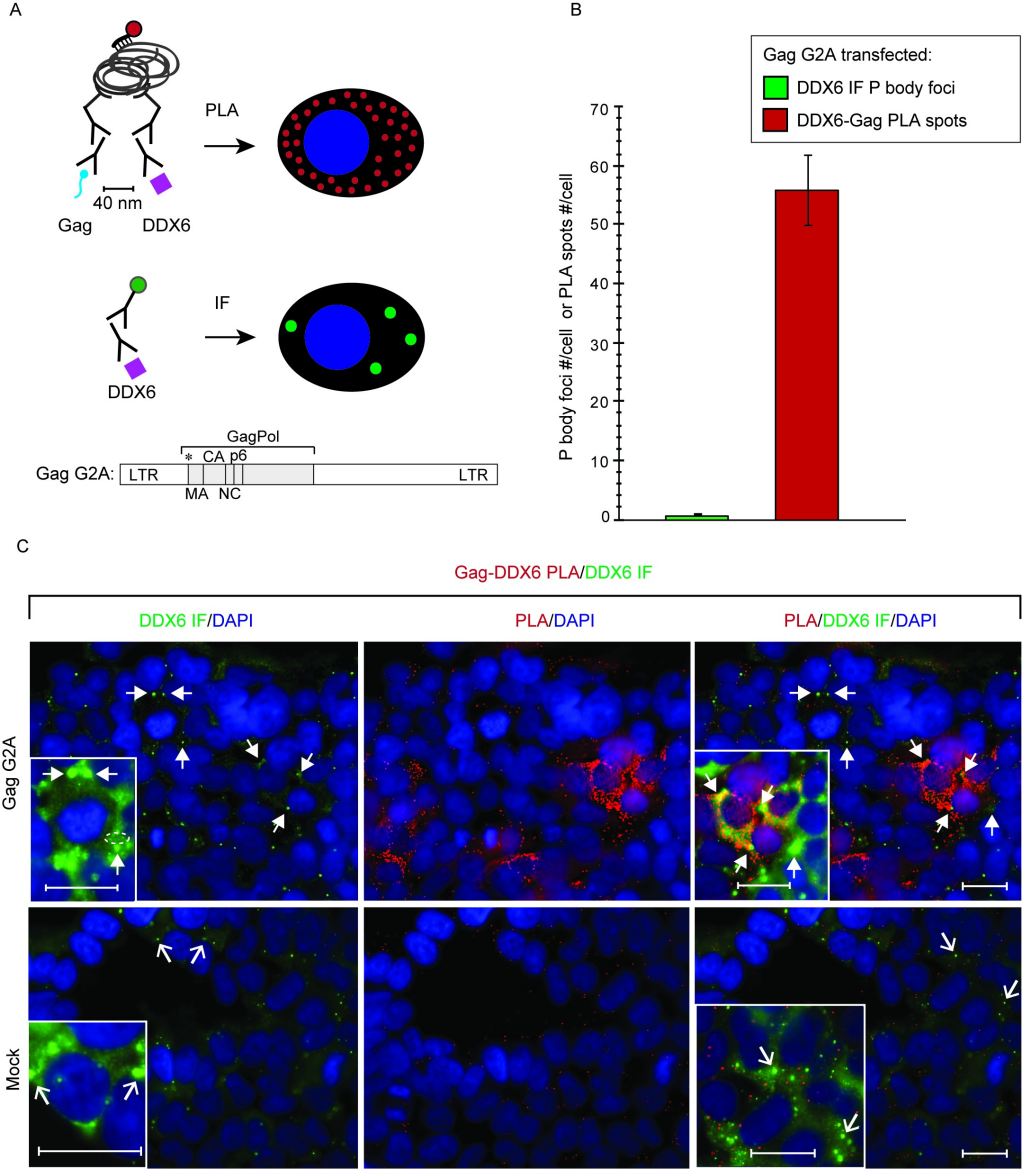
Complexes containing DDX6 colocalized with Gag G2A are far more numerous than DDX6-containing P bodies. (**A)** Gag-DDX6 PLA was used to identify regions in which Gag is within 40 nm from DDX6 *in situ*, with concurrent αDDX6 IF for quantification of P body foci. An experimental schematic for PLA methods is shown (see text for details). 293T cells were transfected with the indicated G2A provirus (Set 1 constructs in Fig 1A) or mock transfected. **(B)** The average number of P body foci per cell (green bar) or PLA spots per cell (red bar) was determined for all Gag-positive cells in five randomly chosen fields. (**C)** Representative images. From left to right for Gag G2A and Mock: DDX6 IF to detect P body foci (green) overlaid with nuclei (blue), PLA signal (red) overlaid with nuclei (blue), and a merge of all three. Insets in DDX6 IF panels in the first column show higher gain/higher magnification versions of cells above the insets, with specific P bodies indicated by arrows at different angles to allow P bodies in insets to be matched to P bodies in low power fields. The dotted oval shows an example of three small granular foci that are too small to be P bodies (i.e. not visible in low gain/low power image), but are visible as discrete small foci when gain and magnification are increased. Insets in merged panels show higher gain/higher magnification versions of cells to the right, with specific P bodies indicated by arrows at different angles to allow P bodies in inset to be matched to P bodies in low power fields. Scale bar, 5 μm. Data are representative of three independent replicate experiments. Error bars show SEM (n = 5 fields).

DDX6 is known to be a marker of P bodies, as shown by high intensity labeling of P bodies by DDX6 IF [24]; however, our studies suggested that DDX6 is also found in smaller RNA granules (Fig 8 and [22]). Additionally, because cells typically contain fewer than ten P bodies [59], our finding that each Z stack image contains ~50 Gag-DDX6 PLA spots (Fig 10) suggested that granules containing Gag and DDX6 are far more numerous than P bodies. While PLA spots are likely not an exact measure of the number of DDX6-containing granules, the relative ratio of DDX6-Gag PLA spots vs. P body spots, and the extent of overlap between the two, should provide insight into whether the putative ~80S assembly intermediates containing Gag and DDX6 correspond to P bodies. To determine this ratio, we analyzed 293T cells for Gag G2A-DDX6 PLA spots with concurrent DDX6 IF, to allow detection of PLA spots and P bodies in the same fields (Fig 10A). G2A provirus was used here because Gag G2A is arrested as an ~80S assembly intermediate that likely contains Gag G2A and DDX6 in association with unspliced HIV-1 RNA (Fig 3B, 3C, 3E and 3F); thus, most Gag G2A-DDX6 PLA spots are likely to represent ~80S assembly intermediates containing Gag G2A, DDX6, and unspliced HIV-1 RNA. We found that each Z stack image from G2A-expressing cells displayed an average of 56 Gag-DDX6 PLA spots, but only one P body by DDX6 IF (Fig 10B and 10C). Interestingly, DDX6 IF images with high gain revealed an abundant diffuse, low-intensity, granular DDX6 signal (e.g. in dotted circle in inset in Fig 10C top row, far left panel), in addition to the bright, DDX6-positive P body spots found in both G2A-expressing and mock cells (Fig 10C, arrows in multiple panels). While Gag-DDX6 PLA spots occasionally overlapped with P body spots (Fig 10C top row, far right panel), most of the Gag-DDX6 PLA spots overlapped with the diffuse, low-intensity, granular DDX6 signal, which includes small foci (Fig 10C top row, merge) and also likely includes soluble DDX6 observed in gradients (S5D Fig). Since we have previously shown by coIP that Gag and DDX6 in the soluble fraction are not associated [22], most likely soluble pools of DDX6 contribute to the DDX6 and Gag PLA colocalization signal in Fig 8 at only a low background level. Instead, most of the PLA signal likely represents colocalization of Gag with DDX6 in small low-intensity foci observed by DDX6 IF (Fig 10C). Thus, we hypothesized that these small low-intensity foci likely correspond to DDX6-containing ~80S RNA granules that are co-opted to form ~80S assembly intermediates. To determine whether the low intensity DDX6 IF signal represents more than just background fluorescence, we compared total DDX6 IF signal in Gag-expressing 293T cells obtained by labeling with αDDX6 followed by fluorophore-conjugated secondary antibody (Total signal) to background signal obtained with secondary antibody alone (S7A–S7D Fig). P body signal (high intensity DDX6 IF signal) was also quantified. These comparisons revealed that the low intensity DDX6 IF signal accounts for 66% of total signal and is distinct from and significantly greater than background (S7B–S7D Fig; p value < 0.01, with background signal accounting for 32% of total). Interestingly, the low intensity DDX6 IF signal accounts for the majority of overall signal, with high intensity P body signal accounting for only ~2% of overall DDX6 IF signal (S7B–S7D Fig). Overall, the PLA and IF data indicate that the ~80S assembly intermediates that likely contain Gag and DDX6 in association with unspliced HIV-1 RNA are far more numerous than P bodies; moreover, most of the Gag-DDX6 PLA signal overlaps with low intensity DDX6 IF signal, which includes a population of small DDX6-positive foci that are visible by light microscopy and therefore likely represent small RNA granules.

### *In situ* association of unspliced viral RNA with DDX6 at the PM upon expression of WT Gag but not Gag Zip

**Fig 11:**
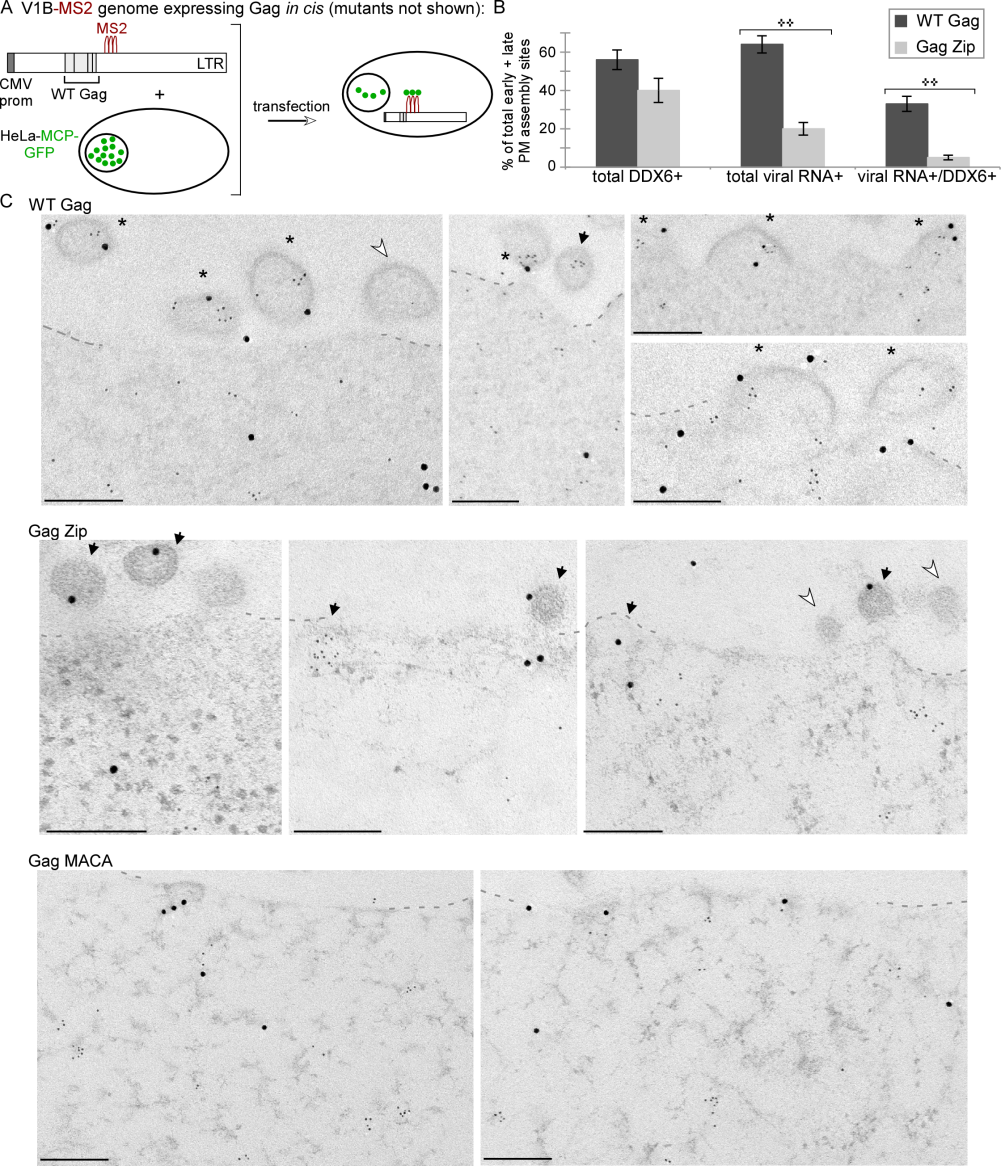
The association of DDX6 with unspliced viral RNA is observed *in situ* at PM assembly sites for WT Gag but not for Gag Zip. **(A)** HeLa cells expressing MCP-GFP were transfected with V1B genomes that contain MS2 binding sites (Set IV constructs in Fig 1A) and express WT Gag (shown), Gag Zip, or MACA. Cells were analyzed by double-label IEM, using αDDX6 and αGFP, which allowed detection of DDX6 (large gold) and MCP-GFP-labeled unspliced viral RNA (small gold), respectively. All early and late assembly sites at the PM were identified in ten cells (~250 μm of PM total per group), and scored for labeling of unspliced viral RNA, DDX6, and double labeling. (**B)** Graph shows the percentage of all early and late PM assembly events that display DDX6 labeling (total DDX6+), labeling of unspliced viral RNA (total viral RNA +), or double labeling (viral RNA+/DDX6+). (**C)** Images show representative assembly sites at the PM for each group, with symbols indicating early or late PM assembly sites that are single-labeled for either unspliced viral RNA or DDX6 (dark arrows), double-labeled (asterisks), or unlabeled (open arrows). Dotted lines outline the PM. Scale bars, 200 nm. Error bars show SEM (n = 10 cells). ++ indicates a significant difference between WT Gag and Gag Zip (p<0.005). For additional data, see S1 Table.

Our biochemical studies showed that WT Gag remains associated with viral-RNA-containing RNA granules during late stages of assembly (Fig 4A–4C), suggesting that assembling Gag takes the co-opted viral-RNA-containing granule to PM sites of budding and assembly. In contrast, we found that Gag Zip associates with RNA granule proteins at late stages of assembly, but not with the subset of RNA granules that contains unspliced viral RNA (Fig 7). Thus, we would expect *in situ* approaches to reveal DDX6-containing assembly intermediates to be present at WT Gag or Gag Zip PM sites of assembly; however, the DDX6-containing granules formed by WT Gag should be colocalized with unspliced viral RNA, while the DDX6-containing granules formed by Gag Zip should not be colocalized with unspliced viral RNA. Previously, we used quantitative IEM to demonstrate that RNA granule proteins (DDX6, AGO2, and ABCE1) are recruited to sites of WT Gag and Gag Zip assembly at the PM [15,18–20,22]. However, these earlier studies did not assess whether unspliced viral RNA is also associated with these RNA granules. Here we used quantitative IEM with double labeling for viral RNA and DDX6 to ask whether viral RNA is associated with DDX6-containing assembly intermediates at PM sites of assembly for WT Gag vs. Gag Zip *in situ* (Fig 11). Notably, to preserve epitopes for IEM, reduced levels of fixatives are required compared to standard EM; this in turn preserves fewer ultrastructural details (as shown previously; compare Fig 2 to Fig 6 in Klein, 2011 #961). While electron dense capsid structures at PM sites of budding and assembly can be seen in many fields, definitive identification of true budding sites is more difficult in such images if Gag is not immunolabeled. However, our previous quantitative IEM studies showed that immunolabeled DDX6 specifically colocalizes with immunolabeled Gag at PM sites of assembly, relative to low background levels of DDX6 at the PM in cells expressing targeting-defective or assembly-incompetent Gag mutants [19,22]. Thus, our previous studies established that PM sites displaying distinctive budding structures in association with DDX6 typically represent sites of Gag assembly.

To test whether V1B RNA colocalizes with PM sites of WT Gag and Gag Zip assembly, we transfected HeLa cells stably expressing MCP fused to GFP (HeLa-MCP-GFP cells) with V1B genomic constructs encoding MS2 binding sites and Gag *in cis* (Fig 11A; Set IV constructs in Fig 1A; phenotypes confirmed in S8 Fig). These cells were utilized because they had been validated previously in live imaging studies of HIV-1 packaging [12]. Sections were labeled with αDDX6 (large gold) to mark RNA granules, and with αGFP to mark MCP-GFP-tagged unspliced viral RNA (small gold). PM assembly sites, defined by the presence of membrane deformation consistent with budding, were quantified and scored for viral RNA labeling, DDX6 labeling, and double labeling (Fig 11B and 11C; S1 Table). Many of these sites displayed distinctive electron dense capsids, as expected at sites of Gag assembly (Fig 11C). When all DDX6 labeling events at PM assembly sites were quantified, similar high levels of DDX6 labeling were observed at both WT and Gag Zip PM assembly sites (56% vs. 40% of all WT vs. Gag Zip PM assembly sites displayed DDX6 labeling, respectively, p>0.01; shown as total DDX6+ in Fig 11B; shown as D+ tot in S1 Table). Notably, quantitation of unspliced viral RNA labeling at all PM sites revealed that unspliced viral RNA was significantly more common at WT assembly sites relative to Gag Zip PM assembly sites (64% vs. 20% of all WT vs. Gag Zip PM assembly sites displayed viral RNA labeling, respectively, p<0.005; shown as total viral RNA+ in Fig 11B; shown as g+ Tot in S1 Table). Our most striking results were obtained upon quantitation of PM assembly sites that were double labeled for DDX6 and viral RNA. Abundant double labeling of unspliced viral RNA and DDX6 was observed at WT PM assembly sites, but not at Gag Zip PM assembly sites (33% vs. 5% of all WT vs. Gag Zip PM assembly sites, respectively, displayed double labeling, p<0.005; shown as viral RNA+/DDX6+ in Fig 11B; shown as g+D+ in S1 Table). As expected, the assembly-incompetent MACA formed very few early or late PM assembly sites, unlike WT and Gag Zip. The same patterns were observed when early and late assembly sites were analyzed separately (S1 Table). Thus, IEM analysis of PM assembly sites supports our conclusion that both WT Gag and Gag Zip co-opt DDX6-containing RNA granules during packaging and assembly, but only the RNA granules co-opted by WT Gag also contain unspliced viral RNA. Moreover, these quantitative IEM studies (Fig 11) along with our PLA studies (Figs 8–10), which were all performed without PuroHS treatment, demonstrate that HIV-1 Gag and unspliced HIV-1 RNA are associated with RNA granules *in situ*.

## Discussion

**Fig 12:**
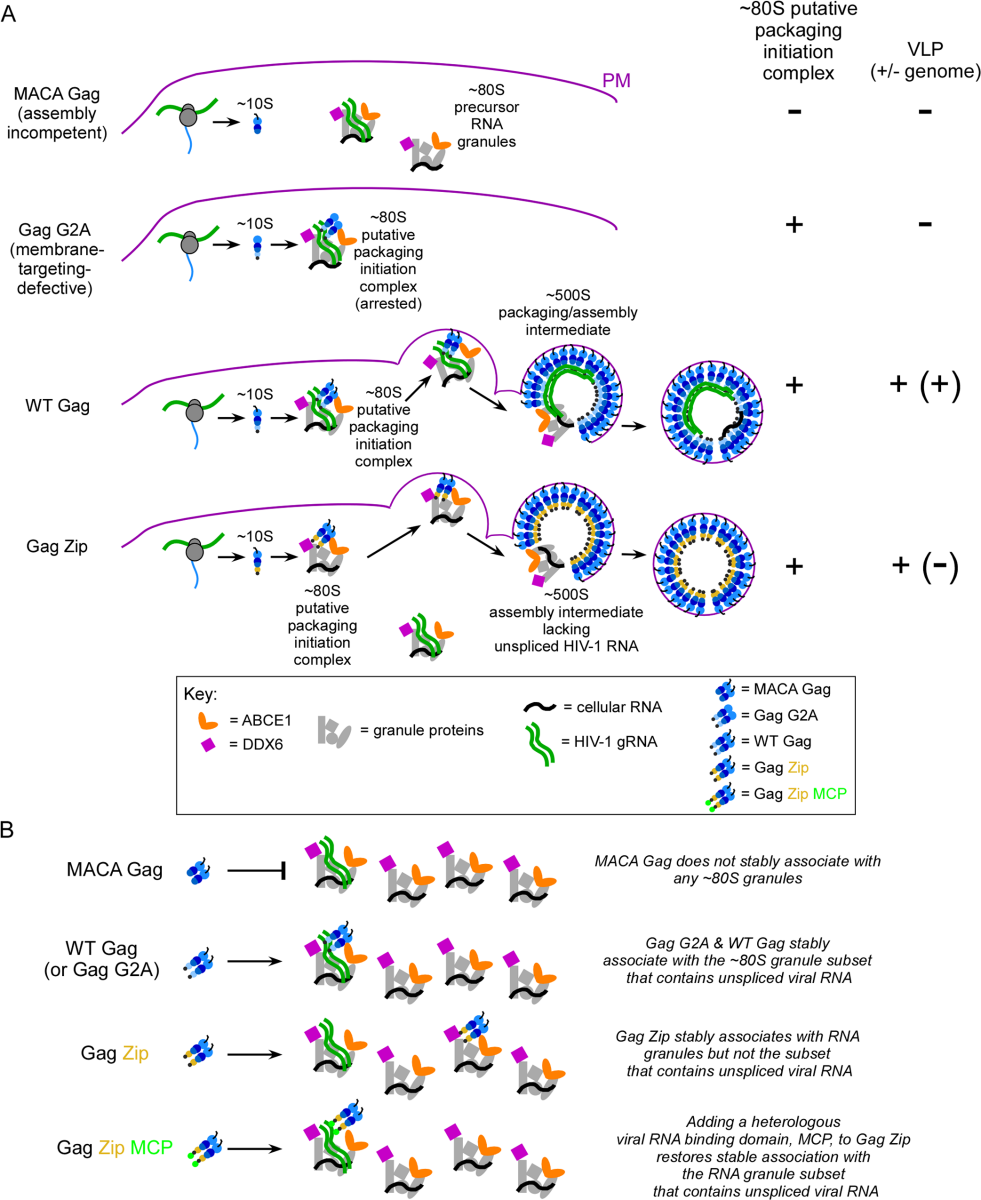
Hypothetical model for how packaging of unpsliced HIV-1 RNA could be intiated within a subclass of host RNA granules. **(A)** MACA Gag, which lacks NC, fails to associate with RNA granules. In contrast, the targeting-defective Gag G2A mutant associates with ~80S RNA granules that contain unspliced viral RNA, leading to formation of the ~80S assembly intermediate in the cytoplasm, without further progression in the assembly pathway. In our studies, this ~80S assembly intermediate was the first assembly intermediate in which Gag associates with unspliced HIV-1 RNA, raising the possibility that the ~80S assembly intermediate is the packaging intiation complex. WT Gag also associates with RNA granules and forms the ~80S putative packaging initiation complex in the cytoplasm, which is then targeted to the PM where Gag multimerizes to complete assembly. RNA granule proteins dissociate from the fully assembled capsid prior to virus budding and release. Like WT Gag, Gag Zip targets to RNA granules, but because Gag Zip contains a protein-protein dimerization domain in place of the viral-RNA-binding NC domain, it is unable to target to the subset of ~80S granules that contains unspliced viral RNA. Ultimately, Gag Zip also undergoes PM targeting, assembly, dissociation of RNA granule proteins, and VLP release; however, Gag Zip VLPs lack viral RNA because the viral-RNA-binding-deficient Gag Zip may have targeted to the “wrong” subset of ~80S granules. Note that we have not defined the components of the RNA granule that contains unspliced viral RNA in each setting; for simplicity, we show the components we have defined for unspliced viral RNA in the context of WT Gag. (**B)** The two-step model for initial targeting of Gag to viral-RNA-containing RNA granules is shown in more detail. Only a subset of ~80S RNA granules contain viral RNA. While MACA Gag does not associate with any RNA granule subsets, G2A and WT Gag target to the subset of RNA granules that contains unspliced viral RNA. Gag Zip targets to RNA granules regardless of whether they contain unspliced viral RNA; however, a heterologous viral-RNA-binding domain (MCP) is able to rescue Gag Zip localization, allowing it to target to ~80S granules containing unspliced viral RNA.

Previously, we identified a pathway of sequential (early, intermediate, and late) HIV-1 capsid assembly intermediates and used this temporal pathway to understand intracellular events in the assembly of the immature HIV-1 capsid. Since the process of HIV-1 genome packaging occurs at the same time as capsid assembly, we reasoned that assembly intermediates are likely to be packaging intermediates; thus, identification of the assembly intermediate in which HIV-1 Gag first associates with unspliced HIV-1 RNA could shed light on the nature of the earliest HIV-1 packaging intermediate. If the first assembly intermediate (~10S Gag) is associated with unspliced HIV-1 RNA, that would support a model in which a Gag monomer or dimer alone initates this association in cells. In contrast, if the initial association of Gag with unspliced HIV-1 RNA occurs in any other assembly intermediate besides ~10S Gag, that would support a model in which Gag first associates with HIV-1 RNA in host ribonucleoprotein complexes, since all the intermediates except the first assembly intermediate contain host RNA granule proteins. Here, using a variety of approaches, we were unable to identify a population of soluble ~10S Gag associated with unspliced HIV-1 RNA; indeed, to the limit of our detection, almost no unspliced HIV-1 RNA was detected in the soluble fraction when gradients that fully resolve the soluble region were analyzed. Instead, the first assembly intermediate in which Gag was associated with unspliced HIV-1 RNA was the second assembly intermediate, an RNA-granule-derived ~80S complex. Consistent with these findings, ABCE1 and DDX6, which are associated with Gag in the ~80S intermediate, were also associated with unspliced HIV-1 RNA in ~80S fractions. Based on our findings, we hypothesize that the first association of HIV-1 Gag with unspliced HIV-1 RNA occurs only after Gag localizes to an RNA-granule-derived complex containing unspliced HIV-1 RNA (the RNA granule model, Fig 12). If this is the case, then the ~80S assembly intermediate could be the first complex in which Gag associates with the HIV-1 RNA that ultimately gets packaged, here termed the packaging initiation complex. While more studies will be needed to determine whether the ~80S RNA-granule-derived assembly intermediate actually is the packaging initiation complex, our findings advance the field by identifying, for the first time, a candidate packaging initiation complex that can be analyzed further.

While we did not identify a complex in which soluble Gag associates with unspliced HIV-1 RNA, our search for such a complex was extensive. We examined lysates generated from two different transfected cell types (COS-1 and 293T cells); lysates expressing WT Gag or two well-studied assembly-defective Gag mutants that are arrested in association with unspliced HIV-1 RNA (one arrested in the cytosol and one arrested at the PM); conditions that largely dissociated ribosomes (through use of PuroHS) versus conditions that preserve ribosomes intact; and cells expressing the HIV-1 provirus or a Gag construct with the viral genome provided *in trans*. Under all these conditions, the smallest complex containing WT or mutant Gag associated with unspliced viral RNA that we could detect at steady state by αGag IP formed a peak in the ~80S region (Figs 3C, 4C, 4F, 5C, 5F, 7C and 7F). When lysates were harvested under gentle conditions, this complex formed a sharp peak in the ~80S region of our gradients (Fig 5E and 5F) and closely corresponded to the previously described ~80S assembly intermediate, the second assembly intermediate in the HIV-1 capsid assembly pathway. However, when the same cell type expressing the same plasmids was harvested using PuroHS treatment, the peak appeared considerably broader, and encompassed the ~40S region in addition to the ~80S region (e.g. Fig 4B and 4C, green lines). There are two possible explanations for the smallest complex containing Gag and unspliced HIV-1 RNA sometimes migrating as a narrow ~80S peak and sometimes migrating as a broader ~40S - ~80S peak. One possibility is that the smallest complex containing Gag associated with unspliced HIV-1 RNA is a partially hidden ~40S complex that is not well resolved; an alternate possibility is that the smallest complex containing Gag associated with unspliced HIV-1 RNA is an ~80S intermediate whose integrity has been partially disrupted by PuroHS, resulting in its migration in an ~40S – 80S region rather than in the sharper ~80S peak we have previously observed. We favor the latter explanation because we have found previously that intracellular assembly intermediates formed by HIV-1 and other retroviral Gag proteins are quite labile, and can be partially disrupted by solutions of high or even modest ionic strength [19,60]. Indeed, when isolated ~80S assembly intermediates formed by some HIV-1 Gag mutants are subjected to an ionic stress considerably less harsh than what was used here, the ~80S Gag is found in a trail that extends into the ~10S-~40S region (e.g. Fig 7D in [19]). Nevertheless, definitively distinguishing between these two possibilities is difficult here because most of the biochemical experiments shown here were performed with PuroHS treatment to ensure that we were not studying ribosome-associated complexes (e.g. Figs 3A–3F, 4A–4F, 5A–5C, 6A–6C and 7A–7F and S2A–S2C Fig). Thus, while the smallest complex containing Gag associated with unspliced HIV-1 RNA that we detected forms a peak in the ~80S region of gradients, more detailed studies using gentle harvest conditions or cross-linkers will be needed in the future to fully resolve the question of whether this represents a single ~80S complex or multiple complexes of ~40S - ~80S.

A second issue worthy of discussion involves our identification of a complex in chronically HIV-infected human T cells that was immunoprecipitated using αABCE1, contains unspliced HIV-1 RNA, and appears to correspond to the ~80S assembly intermediate (with formation of a sharp ~80S peak) (Fig 6B and 6C). In these T cells, our gradient analysis did reveal small pools of unspliced HIV-1 RNA in the 10S - 40S region (Fig 6B). Those pools of unspliced HIV-1 RNA were not apparent in other experiments (e.g. (compare Figs 3B and 5E to Fig 6B). For three reasons, we do not think these pools of unspliced HIV-1 RNA in Fig 6B correspond to Gag-associated complexes of ~10-40S. First, these minor pools of RNA in the 10S - 40S region were observed when cells were harvested in harsh salts as part of PuroHS treatments; thus, the minor pools of RNA likely came from ~80S complexes that were disrupted. Second, unspliced HIV-1 RNA in the 10S - 40S region was only observed in gradients that do not resolve this region well; whenever we have utilized gradients that resolve the 10S - 40S region well (e.g. Fig 3, S4 Fig), we failed to detect unspliced HIV-1 RNA in the ≤40S region of the gradient. Third, αGag IPs failed to immunoprecipitate unspliced HIV-1 RNA from 10S - 40S fractions in chronically infected human T cells (S4 Fig). Thus, to date we have been unable to demonstrate that minor pools of unspliced HIV-1 RNA in the 10S - 40S region are Gag-associated. Nevertheless, we plan to study small Gag and RNA complexes, especially in chronically infected human T cells, in more detail. Ultimately, we cannot exclude the possibility that our experiments failed to identify or selectively lost a soluble complex containing Gag associated with unspliced HIV-1 RNA, or that such a complex is extremely transient and not detectable at steady state by the approaches we used, or that a buried peak is present in the ~40S region of our gradients. However, given that all our lysates contained a large pool of soluble Gag, and that soluble Gag was not associated with unspliced viral RNA to an extent detectable by our RT-qPCR analyses in any of our experiments, our data support a model in which the first association between Gag and unspliced HIV-1 RNA occurs not in the soluble fraction but in a host ribonucleoprotein complex, with more work being needed to determine if this is this host ribonucleoprotein complex is ~40S or ~80S. Moreover, to our knowledge, a soluble intracellular complex that contains Gag in association with unspliced viral RNA has not been identified and reported to date.

Importantly, a model in which HIV-1 RNA packaging is initiated in a host ribonucleoprotein complex (Fig 12), supported by our studies, is also in keeping with important concepts in cell biology. Specifically, cell biologists argue that, within cells, the fate of a particular cellular mRNA is determined in large part by its associated cellular proteins (reviewed in [27]), which first interact with cellular mRNA during transcription and subsequently undergo successive rounds of remodeling (reviewed in [26]). Consistent with these cell biological studies of host mRNA, our model proposes that unspliced HIV-1 RNA first associates with host ribonucleoproteins in the nucleus, and upon entering the cytoplasm is found either in translating or non-translating host ribonucleoprotein complexes. In the absence of assembling Gag, complexes that contain unspliced HIV-1 RNA are of diverse sizes >~30S. The components and identities of these complexes remain to be determined. Based on studies presented here along with our previous studies, we hypothesize that WT Gag first interacts with unspliced HIV-1 RNA to initiate packaging when it co-opts a subpopulation of the non-translating complexes containing unspliced HIV-1 RNA to form the ~80S assembly intermediate, which would then become the packaging initiation complex (Fig 12A). Targeting, packaging, and late stages of WT Gag multimerization continue at the PM in association with this RNA granule, leading to formation of the ~500S packaging/assembly intermediate and the ~750S completely assembled capsid, both of which contain unspliced HIV-1 RNA. The ~80S assembly intermediate/putative packaging initiation complex and the ~500S late packaging/assembly intermediate contain ABCE1 and DDX6, two host enzymes that facilitate virus assembly [21,22] and may distinguish this subclass of granules from other host ribonucleoprotein complexes. Upon completion of immature capsid assembly, the RNA granule proteins dissociate from the ~750S capsid [15,17,21,22], which then undergoes budding and release. Like WT Gag, Gag G2A forms the ~80S putative packaging initiation complex, but is arrested at that step. In contrast, MACA, which lacks NC and is oligomerization-incompetent, fails to associate with RNA granules of any kind. Interestingly, Gag Zip, which also lacks NC but is assembly-competent because it contains an oligomerization domain in place of NC, targets to granules that lack unspliced viral RNA via a mechanism that is independent of NC (Fig 7C; [18,22]).

Our additional studies of Gag Zip led us to define two steps that direct Gag proteins to ~80S granules containing unspliced HIV-1 RNA (Fig 12B). While Gag Zip targets to RNA granules that lack unspliced viral RNA, addition of a heterologous viral-RNA-binding domain redirects Gag Zip to RNA granules that contain unspliced viral RNA (Fig 7F). Therefore, we hypothesize that most ~80S RNA granules likely contain cellular RNA, with only a small subset containing unspliced viral RNA. Together, our data argue that two targeting events are required for stable association of Gag with granules containing unspliced HIV-1 RNA. One step is a poorly understood, oligomerization-dependent step that requires NC or a heterologous oligomerization domain and allows Gag to target to a large class of ~80S ABCE1- and DDX6-containing RNA granules, most of which lack unspliced HIV-1 RNA. The other step involves HIV-1-RNA-binding, which requires NC or a heterologous viral-RNA-binding domain and enables stable association of Gag with a subset of these granules that contains unspliced viral RNA. While MACA lacks the ability to complete either step in this two-step process, Gag Zip is capable of completing the oligomerization-dependent step, but not the step dependent on viral RNA binding (Fig 12B). Thus, a poorly understood feature of Gag that is present in Gag Zip but lacking in MACA is responsible for targeting Gag to a broader subclass of ABCE1- and DDX6-containing host RNA granules that lack unspliced HIV-1 RNA and likely contain cellular mRNA.

Importantly, we do not know the mechanism by which unspliced HIV-1 RNA associates with host RNA granules. Most likely, ribonucleoprotein complexes containing unspliced HIV-1 RNA are generated during transcription, as is the case for cellular mRNA, and undergo successive rounds of remodeling, both during nuclear export and in the cytoplasm, to form cytoplasmic >30S non-translating RNA granules. Additionally, while our coIP studies demonstrate that Gag is associated with unspliced HIV-1 RNA in the packaging/assembly intermediates, we do not know whether Gag and unspliced HIV-1 RNA make direct contact with each other in these complexes. Based on studies by others [61], we speculate that Gag proteins make direct contact with only a few regions of unspliced HIV-1 RNA in the ~80S putative packaging initiation complex, but contact many more regions of the unspliced HIV-1 RNA in the ~500S late packaging intermediate. Also, because we did not analyze nuclear complexes, we cannot exclude the possibility that Gag first forms the ~80S putative packaging initiation complex in the nucleus; however, like others [13], we have not observed enough Gag in the nucleus of provirus-expressing cells to test this possibility.

Some other aspects of our study deserve further comment. First, PuroHS treatment was used in many of our experiments because it allows us to exclude translating complexes, which migrate at similar sizes as our assembly/packaging intermediates and therefore prevent us from studying non-translating RNA that undergoes packaging. Excluding translating complexes is particularly important when analyzing provirus-expressing cells, in which Gag would be expected to associate with unspliced HIV-1 RNA during its translation and also during packaging. However, our use of Gag GFP constructs that package a modified viral genome *in trans* allowed us to show that the same results were obtained when we repeated key experiments without PuroHS treatment (compare Fig 5D–5F performed without PuroHS treatment to Fig 4A–4C performed with PuroHS treatment). In the experiment performed without PuroHS treatment, the *in trans* system allowed us to still exclude translating Gag from our analyses of complexes containing Gag and unspliced viral RNA because Gag in that system is translated from an mRNA with a different nucleotide sequence that is not detected by our RT-qPCR oligo. Our demonstration that the ~80S complex containing Gag associated with unspliced viral RNA is present even in cells harvested with ribosomes intact but with translating Gag mRNA excluded from the analysis through oligo design (Fig 5F) indicates that the ~80S candidate packaging initiation complex is not an artifact of PuroHS treatment.

We should also note that differences in accessibility of epitopes during IP could account for differences in migration of immunoprecipitated complexes relative to complexes shown in Gag blots and profiles of total unspliced viral RNA. For example, the difference in the exact migration of the ~80S peak in Fig 4C (in which αGag was used to IP the ~80S complex) and S2C Fig (in which αABCE1 was used to IP the ~80S complex) could be explained by ABCE1 in the ~80S complex being less accessible than ABCE1 in the ~500S complex (while conversely Gag in the ~80S complex is known to be more accessible than Gag in the ~500S complex). Performing more detailed studies to address whether the ~80S peak is heterogeneous and define the basis of differences in epitope accessibility are of high priority in the future. Additionally, we should also emphasize that the pool of all complexes that contain unspliced HIV-1 RNA in the absence of Gag is very diverse, with complexes ranging in size from 30S to >150S (Fig 2C). In the presence of assembling Gag, it is likely that only a small percentage of these complexes become complexes containing Gag associated with unspliced HIV-1 RNA. Finally, with respect to the putative ~500S late packaging intermediate, as mentioned earlier, the ratio of this complex relative to the ~80S complex also varied considerably in our experiments. This is not surprising since the amount of the late packaging intermediate present at steady state could be highly dependent on the kinetics of assembly and budding, both of which could vary with cell type, stage of assembly, cell viability, etc. Interestingly, peaks corresponding to both the ~80S and ~500S putative packaging intermediates were particularly prominent in human 293T cells and infected human T cells (Figs 5C and 6C).

Notably, the ~80S assembly intermediate in which we first detected Gag associated with unspliced HIV-1 RNA (the candidate packaging initiation complex) has an S value similar to the eukaryotic ribosome, which is a large ribonucleoprotein complex containing four species of RNA and 79 cellular proteins. Interestingly, studies by others are consistent with this finding. Specifically, the reported diffusion coefficient of ~70S bacterial ribosomes (0.04 μm^2^ sec^-1^; [62]) is similar to the ~0.07 and 0.014 μm^2^ sec^-1^ diffusion coefficients reported for cellular subpopulations of HIV-1 RNA [63] and Gag [64], respectively. Our finding that the packaging initiation complex is ~80S suggests that this complex contains numerous host components, since Gag is likely a dimer or small oligomer at this stage [13], and a Gag dimer on its own would be ~5S. Although we know that two other viral proteins, HIV-1 GagPol [15] and Vif [21], are present in assembly intermediates, it is likely that host components account for most of the molecular mass of the ~80S packaging initiation complex. Here we showed that the candidate packaging initiation complex likely contains at least two host enzymes that are known to facilitate HIV-1 capsid assembly, ABCE1 and DDX6 [21,22]. In addition, our earlier studies demonstrated that the RNA granule proteins AGO2 and DCP2 are also present in the ~80S and ~500S RNA-granule-derived assembly intermediates [22]. Interestingly, we recently showed that the small ribosomal S6 protein was not detected in IP analyses of RNA-granule-derived ~80S and ~500S capsid assembly intermediates formed by a non-human lentiviral Gag (feline immunodeficiency virus; [60]). The lack of ribosomal proteins, along with the presence of RNA granule proteins, in capsid assembly intermediates further supports a model in which assembling retroviral Gag proteins co-opt a host RNA granule during assembly/packaging.

Importantly, our *in situ* studies demonstrate that the RNA-granule-derived putative HIV-1 packaging intermediates that we identified are not simply artifacts formed during cell lysis. Previously, we had used quantitative double-label IEM to show that DDX6 is recruited to PM sites of WT Gag assembly, but not to PM sites containing assembly-incompetent MACA Gag [19,22]. Here we showed that these PM sites of assembly, detected because they contain DDX6 at sites of PM deformation typical of HIV-1 budding, also contain unspliced viral RNA, thus demonstrating colocalization of unspliced viral RNA with an RNA granule protein at likely PM assembly sites *in situ* (Fig 11). Moreover, because some investigators define RNA granules as complexes containing non-translating RNA that are detectable as discrete foci by light microscopy, we also developed approaches for asking whether complexes that contain Gag colocalized with DDX6 correspond to fluorescent foci visible by light microscopy. First we showed that we can detect Gag colocalized with DDX6 (or ABCE1*) in situ* by light microscopy using PLA (Figs 8 and 9). Notably, our biochemical studies suggested that the ~80S putative packaging initiation complex should be similar in size to the ~80S ribosome, which is ~25 nm in diameter [65], i.e. roughly one-quarter or one-twelfth the size of a DDX6-containing P body (which ranges from 100–300 nm in size [66]). For this reason, we hypothesized that the ~80S assembly intermediate/putative packaging initiation complexes might be detectable as small fluorescent foci that are distinct from large P bodies but nevertheless still visible by light microscopy. Consistent with this hypothesis, we found that indeed the vast majority of ~80S Gag G2A-DDX6 PLA spots overlapped not with P bodies, but with a lower intensity granular DDX6 signal (Fig 10) that accounts for the majority of DDX6 IF signal. Moreover, this low intensity DDX6 signal includes discrete small cytoplasmic foci that are distinguishable from background even though they are considerably smaller than P bodies (S7 Fig). Together, our PLA and IF data suggest that the ~80S assembly intermediate/putative packaging initiation complexes likely correspond to small cytoplasmic foci; the finding that these foci are visible by light microscopy and contain the RNA granule protein DDX6 supports our description of them as small RNA granules that are distinct from (but possibly related to) P bodies.

Our demonstration that PLA spots containing Gag colocalized with DDX6 are far more numerous than P bodies is consistent with an earlier study showing that Gag and viral RNA are not found in P bodies [67]. However, the small RNA granules co-opted by assembling Gag could represent subunits that exchange with P bodies or other RNA granules, perhaps explaining an earlier report of HIV-1 RNA colocalizing with P bodies [68]. In keeping with this possibility, we observed that Gag-DDX6 PLA spots occasionally overlap with P bodies (Fig 10). A model in which P bodies consist of smaller functional subunits, such as the small ~80S RNA granules co-opted by HIV-1, is appealing given numerous studies showing that when large P body foci are dissociated and no longer visible, their functions are not lost [69,70], consistent with P bodies being composed of smaller, independent functional units. Thus, for three reasons, it seems appropriate to call these DDX6-containing complexes, including those co-opted by HIV-1, small RNA granules: 1) in IF images they appear to be discrete low intensity foci (Fig 10 and S7 Fig), albeit with sizes that are close to the limit of light microscopic detection; 2) they contain canonical RNA granule proteins such as DDX6 (Fig 8, Fig 10 and S6 Fig) and AGO2 [22]; and 3) they could correspond to the smaller DDX6-containing subunits that others have found to be functional when P bodies are disrupted. Further studies will be required to define the exact proteome and RNAome of these small RNA granules and the ~80S packaging initiation complex. Additionally, crosslinking followed by multiple purification steps will be required to test definitively whether Gag, DDX6, ABCE1, and unspliced viral RNA are all together in the same complex (rather than only two or three of these components being together in two or more separate complexes of similar size). More studies will also be required to determine whether these complexes contain other RNA binding proteins such as Staufen1, which plays a role in HIV-1 packaging and assembly [71–73], or MOV10, which is packaged by HIV-1 and modulates virus infectivity [74,75].

It is also worth noting the similarities between the RNA granule model of HIV-1 packaging proposed here, and what has been observed for the Ty3 yeast retrotransposon, which is distantly related to HIV-1. Assembling Ty3 Gag and its packaged Ty3 RNA are found in large clusters that contain the yeast DDX6 homologue dhh1 [76,77]; moreover knockdown and mutational analyses indicate a role for RNA granule proteins in formation of functional Ty3 retrotransposons in yeast [78,79]. Similarly, we had previously shown that DDX6 acts enzymatically to promote immature HIV-1 capsid assembly, since siRNA knockdown of DDX6 decreased virus production without affecting steady-state Gag levels and virus production was rescued by a siRNA resistant WT DDX6 but not a ATPase-defective DDX6 mutant [22]. Additionally, DDX6 knockdown in primary human T cells reduced production of infectious HIV-1 in that study [22]. While another study did not find DDX6 to be required for HIV-1 capsid assembly [67], this could be because of differences in the extent of DDX6 depletion or because other helicases in the co-opted RNA granule can substitute for DDX6 in some cell types or when given enough time to be upregulated in shRNA-expressing cell lines. Interestingly, human foamy virus was also reported to require DDX6, with DDX6 being necessary for packaging rather than virus particle assembly [80]. Thus, the overall similarity between our data and data obtained for Ty3 and human foamy virus raises the possibility that co-opting of host RNA granules for RNA packaging and assembly could be an ancient mechanism that has been conserved across retroelement evolution.

Together our data argue for a new view of packaging. To date, packaging studies have not specified how or in what complex Gag associates with viral RNA; here we fill this gap by identifying a candidate packaging initiation complex in provirus-expressing cells, that corresponds to a poorly understood host RNA granule. Sequestration of unspliced HIV-1 RNA within a subset of host RNA granules suggests that HIV-1 RNA mimics host RNAs, which also localize to RNA granules when they are not translating. Interestingly, this localization of HIV-1 RNA to host RNA granules could be both beneficial and problematic for the virus. On the one hand, sequestration would allow viral RNA to evade detection by the host immune system, create a site where assembling Gag can be concentrated, and provide Gag access to host RNA helicases that could facilitate displacement of host RNA binding proteins from unspliced HIV-1 RNA, allowing them to be replaced with Gag. On the other hand sequestration of unspliced HIV-1 RNA creates a dilemma for the virus in that it puts the unspliced HIV-1 RNA that must be encapsidated into a different subcellular compartment (the RNA granule) than newly translated Gag, which is initially found either with translating ribosomes or in the soluble compartment. To solve this problem, Gag may have evolved an oligomerization-dependent mechanism for localizing to a subclass of mRNA-containing host RNA granules; association with RNA granules could allow efficient binding of Gag to unspliced HIV-1 RNA, which would in turn localize Gag to the small subset of these granules that contains unspliced HIV-1 RNA. Future studies will be needed to further test this model and better understand how the virus co-opts this subclass of host RNA granules.

## Materials and methods

### Plasmids and cells

Four types of expression systems were utilized in this study. Proviruses (Set I constructs in Fig 1A) are from the LAI strain and were described previously [15,18,81]. These have native HIV-1 sequences and the HIV-1 LTRs. Proviruses were transfected into COS-1 or 293T cells (American Type Culture Collection (ATCC), Manassas, VA) as indicated for some biochemical studies and all PLA studies. For other biochemical studies, we used an *in trans* expression system in which the genome is provided by V1B, a modified proviral plasmid that expresses an RNA encoding an assembly-incompetent truncated *gag* gene, all cis-acting packaging signals, full-length *tat*, *rev*, and *vpu* genes, and 24 MS2 stem loops that bind to MCP [12]. V1B was transfected into COS-1 or 293T cells with WT and mutant SynGag GFP constructs (here referred to as Gag GFP constructs) provided *in trans*, where indicated. V1B and the codon optimized SynGag GFP WT and G2A constructs (Set II constructs in Fig 1A) were provided by P. Bieniasz (Rockefeller University, New York, N.Y.) and were utilized in previous live imaging studies [12] and coIP studies [13]. Additional Gag GFP constructs (MACA and Gag Zip) were generated from the WT SynGag GFP construct. For G2A, the glycine in position 2 of Gag was converted to an alanine by via site-directed mutagenesis, as described previously [15]; for Gag Zip, LZ was inserted in place of NC using Gibson assembly, as described previously [18]. For IEM experiments, HeLa cells that express MCP-NLS-GFP as described previously [12] were obtained from P. Bieniasz (Rockefeller University, New York, N.Y.). To ensure that all HeLa-MCP-GFP cells expressed both Gag and genome following transfection, V1B constructs expressing WT and mutant Gag *in cis* were generated by inserting relevant Gag coding regions from HIV-1 proviruses (Set I constructs in Fig 1A) into V1B constructs containing MS2 binding sites via Gibson assembly to generate Set IV constructs in Fig 1A. Oligos used for site-directed mutagenesis and Gibson assemblies are available upon request.

H9 cells (ATCC) were used to generate chronically HIV-infected H9 T cells expressing a *pro-*genome in cells (Set III constructs in Fig 1A) by infection with virus. Plasmid used for virus production was generated by inserting three protease inactivation mutations into a previously described HIV-1 provirus, LAI strain, that encodes deletions in *env* and *vif*, a frameshift in *vpr*, and substitution of *nef* with a puromycin resistance gene, as described previously [22]. 293T cells were transfected with this plasmid to produce virus, and H9 cells were infected with this virus and maintained under puromycin selection as described previously [17].

COS-1 and 293T cells were maintained in DMEM (Life Technologies) with 10% FBS. H9 cells were maintained in RPMI (Life Technologies) with 10% FBS under puromycin selection. HeLa-MCP-GFP cells were obtained from P. Bieniasz and were maintained in DMEM with 10% FBS, and periodically subjected to blastocidin selection.

### Transfection, IP, and WB

COS-1, 293T, or HeLa-MCP-GFP cells were transfected with 1–5μg DNA using polyethylenimine (Polysciences, Warrington, PA). Cell lysates were harvested in 1X Mg^+2^-containing NP40 buffer (10 mM Tris-HCl, pH 7.9, 100 mM NaCl, 50 mM KCl, 1 mM MgCl, 0.625% NP40) in the presence of freshly prepared protease inhibitor cocktail (Sigma, St Louis, MO) and RNaseOUT (Invitrogen). Where indicated, lysates were treated with 1 mM puromycin HCl (Invitrogen) for 10 min at 26°C followed by 0.5M KCl for 10 min at 26°C before further analysis; otherwise cells were harvested in 1X NP40 buffer (10 mM Tris-HCl, pH 7.9, 100 mM NaCl, 50 mM KCl, 0.625% NP40) in the presence of freshly prepared protease inhibitor cocktail (Sigma, St Louis, MO) and RNaseOUT (Invitrogen) and analyzed immediately. Lysates were analyzed by WB or RT-qPCR, or subjected to IP as described below. Alternatively, lysates were analyzed by velocity sedimentation, as described below, and gradient fractions were then analyzed by WB, RT-qPCR, or IP.

Except where indicated, lysates or gradient fractions were subjected to IP with affinity purified αABCE1 [21], HIV immunoglobulin NIH AIDS Reagents Catalog #3957, from NABI and NHLBI), a monoclonal to GFP (Roche), or αDDX6 (#461, Bethyl Laboratories, Montgomery, TX), using protein G-coupled Dynabeads (Life Technologies). IP eluates were analyzed by SDS-PAGE, followed by western blot (WB) using the primary antibodies described above or a monoclonal antibody directed against HIV-1 Gag p24 (HIV-1 hybridoma 183-H12-5C obtained from Bruce Chesebro through the AIDS Reagent Program Division of AIDS, NIAID, NIH), followed by an HRP-conjugated anti-human IgG secondary antibody (Bethyl Laboratories, Montgomery, TX), or an HRP-conjugated anti-mouse-IgG_1_ (Bethyl Laboratories) or anti-mouse-IgG or anti-rabbit secondary antibody (Santa Cruz Biotechnology, Dallas, TX). In S5C Fig, IP eluates were subjected to WB with HIV immune globulin (provided by NABI and NHLBI, catalog no. 3957 in the AIDS Reagent Program Division of AIDS, NIAID, NIH) for detection of Gag. WB signals from IP eluates were detected using Pierce ECL substrate (Thermo Fisher Scientific) with Carestream Kodak Biomax Light film. For detection of Gag in total cell lysates, velocity sedimentation fractions, and membrane flotation fractions, WBs were performed as described above, or using antibodies conjugated to infrared dyes (LI-COR Biosciences, Lincoln, NE). Quantification of Gag bands on film was performed using Image J software or LI-COR Odyssey software.

### RNA quantification

Transfected cells or VLPs were harvested as described above. Where indicated lysates were analyzed by velocity sedimentation and/or IP, as described above except that IP samples that were prepared for viral RNA quantification were washed four times in detergent buffer and once in non-detergent buffer. For total cell lysate analysis, aliquots corresponding to ~5 x10^3^ COS-1 cells, 2 x10^4^ 293T cells and 8.5 x10^3^ HeLa-MCP-GFP cells were used, and results of quantification were normalized to 1 x10^3^ cells. For VLP analysis, aliquots corresponding to VLPs from 1 x10^5^ 293T cells and 1.5 x10^4^ HeLa-MCP-GFP cells were used, and results of quantification were normalized to 1 x10^3^ cells. For gradient analysis, ~4 x10^5^ COS-1 cells, 2 x 10^6^ 203T cells, or 6 x 10^5^ H9 cells were analyzed on a single 5 ml gradient. Aliquots of gradient fractions or gradient IP fractions were treated with proteinase K (Sigma) at a final concentration of 150 μg/ml in 0.1% SDS, followed by total RNA extraction by using Trizol (Ambion). RNA was precipitated with isopropanol, extracted with BCP (Molecular Research Center), pelleted at 12,000xg for 15 min at 4°C, and the RNA pellet was subjected to DNase I (Invitrogen) treatment (2 u per 50 μl reaction). The iScript Advanced cDNA synthesis kit (Bio-Rad) was used to generate cDNA from 10% of the RNA using random hexamer primers at 42°C for 30 min, followed by heat inactivation. An aliquot of cDNA (2.9%) was used for qPCR using SYBR Green (Bio-Rad) to determine RNA copy number. For HIV-1 viral RNA qPCR, we used the following oligos that target bp 162 to 269 within the Gag open reading frame in HIV-1 LAI (at the end of MA and start of CA) and result in a 108 bp amplicon: 5’-AGAAGGCTGTAGACAAATACTGGG-3’ (forward); 5’-TGATGCACACAATAGAGGGTTG-3’ (reverse). These oligos detect the full length HIV-1 provirus as well as the V1B genomic construct, but do not recognize the Gag GFP constructs that were transfected *in trans*, as expected since the Gag GFP constructs were codon-optimized. To determine the copy number of other RNA species, we used the following qPCR oligos: Tat mRNA, 5’-TCT ATC AAA GCA ACC CAC CTC-3’ (forward) and 5’-CGT CCC AGA TAA GTG CTA AGG-3’ (reverse); 28S rRNA, 5’-CCC AGT GCT CTG AAT GTC AA-3’ (forward) and 5’-AGT GGG AAT CTC GTT CAT CC-3’ (reverse); 18S rRNA, 5’-GCA ATT ATT CCC CAT GAA CG-3’ (forward) and 5’-GGC CTC ACT AAA CCA TCC AA-3’ (reverse); GAPDH mRNA, 5’-AGG TCA TCC CTG AGC TGA AC-3’ (forward) and 5’-GCA ATG CCA GCC CCA GCG TC-3’ (reverse); and 7SL RNA 5’-GCT ATG CCG ATC GGG TGT CCG-3’ (forward) and 5’-TGC AGT GGC TAT TCA CAG GCG-3’(reverse). All qPCR samples were analyzed in duplicate using the MyiQ RT-PCR detection system and iQ5 software (Bio-Rad). Amplicons corresponding to regions amplified by qPCR were used to generate standard curves. Duplicate nine-point standard curves were included on every qPCR plate, ranging from 10^1^ copies to 10^8^ copies, with a typical efficiency of ~90% or greater and an R^2^ of 0.99. Standard curves were able to detect 10 copies per reaction but not 1 copy, thereby setting the detection threshold at 10 copies per reaction, which was equivalent to ~1000 copies per 1000 cells for inputs and total viral RNA from gradient fractions, ~100 copies per 1000 cells for IP from total cell lysates or gradient fractions, and ~50 copies per 1000 cells for VLPs. Minus RNA controls were included in each experiment and were always zero. RT minus controls were also included in each experiment and ranged from 0–100 copies per reaction. Mock transfected VLP controls were used to set the baseline in graphs and ranged from 1–1000 copies per 1000 cells. For IP, nonimmune viral RNA copy number was analyzed in parallel and was typically 1–2 logs lower than in immune IP samples.

Quantification of the total cell number used in each experiment allowed us to represent all qPCR data as number of viral RNA copies per 1000 cells, except for Fig 2 data which is presented as % of total viral RNA to allow comparison across different RNA species. Log scales were used to display all VLP, which exhibit large differences. Linear scales were used for IP data, which exhibit smaller differences. Note that differences in transfection efficiency resulted in a range of total viral RNA copies per 1000 cells between experiments; likewise, IP efficiency also varied between experiments. Thus the exact number of viral RNA copies immunoprecipitated in fractions from different experiments varied considerably, but the pattern did not.

Protocols for VLP and gradient RT-qPCR are available at:

dx.doi.org/10.17504/protocols.io.k73czqn

dx.doi.org/10.17504/protocols.io.k74czqw

### Analysis of VLP production

Supernatants of COS-1 or HeLa-MCP-GFP cells, transfected as described above, were centrifuged at 2000 rpm (910 x *g*) for 10 min at 4°C, filtered (0.45 μm) to remove remaining cells, and purified through a 30% sucrose cushion in an SW60Ti rotor at 60,000 rpm (370,000 x *g*) for 30 min at 4°C, as described previously [18].

### Velocity sedimentation

Transfected COS-1 cells or 293T cells were harvested at 36 h or 15 h posttransfection, respectively, as described above and diluted into 1X NP40 buffer (10 mM Tris acetate pH 7.4, 50 mM KCl, 100 mM NaCl, 0.625% NP-40). For each sample, 120 μl lysate was layered on a step gradient. To resolve complexes of ~10S to ~150S, step gradients were prepared from 5%, 10% 15%, 20%, 25%, and 30% sucrose in NP40 buffer without MgCl (10 mM Tris-HCl, pH 7.9, 100 mM NaCl, 50 mM KCl, 0.625% NP40) and subjected to velocity sedimentation in a 5 ml Beckman MLS50 rotor at 45,000 rpm (162,500 x *g*) for 90 min at 4°C. To resolve from ~10S to ~750S, step gradients were prepared from 10%, 15%, 40%, 50%, 60%, 70%, and 80% sucrose in NP40 buffer without MgCl, and subjected to velocity sedimentation in a 5 ml Beckman MLS50 rotor at 45,000 rpm (162,500 x *g*), for 45 min at 4°C. Gradients were fractioned from top to bottom, and aliquots were analyzed by WB, IP, and/or RT-qPCR as described above. Expected S value migrations were determined using a published equation [40] and confirmed using S value markers, as described previously [16]. These expected S value migrations are shown as black bars above gradient fractions, but were also confirmed by RT-qPCR for 7SL RNA (~11S), 18S rRNA (40S small ribosomal subunit), and 28S rRNA (60S large ribosomal subunit).

### Proximity ligation assay and immunofluorescence

293T cells were plated into 6-well dishes containing coverslips with Grace Biolabs CultureWell silicone chambers (Sigma-Aldrich) attached to create four chambers on each coverslip. Cells were transfected with 3 μg of plasmid per well and 16.5 h later were fixed for 15 min in 4% paraformaldehyde in PBS pH 7.4, permeabilized in 0.5% saponin in PBS, pH 7.4 for 10 min, and blocked in Duolink blocking solution (Sigma-Aldrich) at 37°C for 30 min. Cells were incubated in primary antibody (described under IP methods above), followed by Duolink reagents (Sigma-Aldrich): oligo-linked secondary antibody, ligation mix, and red or green amplification/detection mix, with washes in between, as per the Duolink protocol. For concurrent IF, cells were incubated following the final PLA washes for 15 min at RT with 1:1000 secondary antibody, either Alexafluor 594 conjugated to anti-mouse IgG or Alexafluor 488 conjugated to anti-rabbit IgG. Cover slips were mounted using Duolink In Situ Mounting Media with DAPI, sealed to the glass slides with clear nail polish, allowed to dry for 24 h at RT, and stored at -20°C. Imaging was performed with a Zeiss Axiovert 200M deconvolution microscope using Zeiss Plan-Apochromat 63X/ aperture 1.4 objective with oil immersion, using AxioVision Rel. 4.8 software. For quantification, exposure times were set so that all measured PLA spots and Gag IF signal in test fields fell below saturation. Once a non-saturating exposure time was identified, five fields containing at least three IF-positive cells (for Figs 8 and 9) or PLA-positive cells (for Fig 10) were chosen at random and imaged using identical exposure times for the red channel, and identical exposure times for the green channel (red/green exposures were 1 sec/1.5 sec for Fig 8; 2 sec/1 sec for Fig 9; and 40 msec/250 msec for Fig 10). Images were captured as ten 1-μm Z-stacks centered on the focal point for the PLA. Images were deconvolved using the AxioVision software, then exported as .tif files, and Image J was used to outline Gag-positive cells in each field. Within those positive cells, the central Z-stack image was used to count PLA “spots”, and quantify IF intensity where indicated, using Image J. PLA spot number for each field was then normalized to the average IF intensity within that field, and the results were plotted with error bars representing the SEM for five fields. For Fig 10, Gag-DDX6 PLA was performed either with concurrent Gag IF or concurrent DDX6 IF, and the PLA fields with Gag IF were used for PLA spot quantitation to allow exclusion of background spots in Gag-negative cells, while the PLA fields with DDX6 IF were used for P body quantitation.

Because spots in fields used for quantitation were difficult to see in figures, the gain was increased proportionally in quantified images solely for the purpose of display. Specifically, after imaging and quantification, the red channel gain in representative images was increased proportionally (from 1 to 3 in Fig 8, to 11 in Fig 9, and to 7 in Fig 10) using the AxioVision Rel. 4.8 software for all conditions, to allow better display of red spots in final figures. The same was done for the green channel gain (increased from 1 to 7) to display smaller DDX6 granules in the Fig 8D insets. Images were imported in 8-bit color into Adobe Illustrator to create the final figure layout, without further adjustments to color balance or gamma correction. Data shown are from one experiment that is representative of two independent replicate experiments.

PLA experiments in S6 Fig were carried out as above using the red PLA reagents, with the following modifications. First, where indicated, NI antibodies were used in place of αGag or αDDX6 primary antibodies in PLA negative controls. Secondly, for Gag-Non-immune (NI) PLA, as well as DDX6-Gag PLA in both mock- and Gag-transfected cells, concurrent IF was performed as described above using 1:1000 Alexafluor 488 anti-mouse secondary antibody; however, for NI-DDX6 PLA, a sequential IF was performed, in which, following the final PLA washes, samples were incubated with anti-Gag primary antibody for 30 min at RT. Samples were then washed and incubated for 15 min at RT with 1:1000 Alexafluor 488 anti-mouse secondary antibody. This allowed PLA spots to be counted in Gag-positive cells for both NI conditions. For NI quantification, five fields were chosen at random as above and imaged using identical exposure times for the red channel. The green channel was imaged using identical exposure times for Gag-DDX6 and Gag-NI fields, but exposure time was increased for the NI-DDX6 Gag IF condition to match Gag-DDX6 and Gag-NI signal saturation, since the sequential IF method gave lower overall signal than the concurrent IF method (red/green exposures were 1 sec/1 sec for Gag-DDX6 and Gag-NI; 1 sec/2 sec for NI-DDX6). Also, as above, after imaging and quantitation, the red channel gain was increased proportionally to 3 in the AxioVision Rel. 4.8 software for all conditions for display purposes only, without further adjustments in the final layout.

For IF alone in S7 Fig, 293T cells were plated, transfected as indicated, and harvested as for PLA. However, after blocking, cells were incubated with primary antibody (described under IP methods above) in PLA antibody diluent at 37°C for 1 h (or with buffer for the secondary antibody alone group), followed by four Buffer A and two Buffer B washes. After these washes, cells were incubated with 1:1000 anti-mouse IgG conjugated to Alexafluor 594 and anti-rabbit IgG conjugated to Alexafluor 488 (secondary antibodies) for 30 min at RT. Finally, cells were washed three times with Buffer B and once with 0.01X Buffer B, and then mounted as for PLA. Imaging was also performed as for PLA, with red/green exposures of 1.25 sec/0.5 sec–exposure time for the green channel was lowered from the automatic threshold determined by the imaging software to avoid saturating P body signal. Five fields were imaged for each condition, with each field containing at least two Gag-positive cells. For quantification, two saturation thresholds were chosen in the ImageJ software, one to capture all the DDX6 IF signal in the entirety of all Gag-positive cells, with Gag-positive cells defined by the red channel, and one to capture only the signal from the P bodies within these cells. The total area and mean signal over this area were then determined for the green channel (DDX6 IF or secondary antibody only) using both thresholds in the DDX6 IF condition, and using only the threshold encompassing the entire Gag-positive cell in the secondary-antibody-only condition. These calculations were performed for all five fields for each condition. The mean signal within all Gag positive cells in the DDX6 IF green channel was termed the “P bodies + low intensity signal + background” value (Total signal), and in the secondary antibody only condition was termed the “Background” value (Background signal). To subtract P body signal, for each field the mean total signal and mean P body signal were multiplied by their total area area to get a value for each representing the total DDX6 IF signal intensity in the saturated area, and the P body signal value was subtracted from the total signal value. The area of the P body regions was then subtracted from the area of the Gag-positive regions, and the new P-body-subtracted signal value was divided by the new P-body-subtracted area to calculate a “Low intensity signal + Background” fluorescence intensity value. The means for each of these three values (“P bodies + Low intensity signal + Background” (Total signal), “Low intensity signal + Background”, and “Background”) were then plotted for the five imaged fields as percent of Total signal, with error bars representing the SEM, and p values calculated with n = 5 fields using the Student's t-test (two-tailed).

Protocol for PLA with concurrent IF is available at: *dx.doi.org/10.17504/protocols.io.k7yczpw*

### Quantitative IEM

HeLa-MCP-GFP cells were transfected with the indicated constructs. Cells were harvested at 24 h posttransfection in fixative (3% paraformaldehyde, 0.025% glutaraldehyde in 0.1 M phosphate buffer, pH 7.4), pelleted, and subjected to high pressure freezing using the Leica EMPACT2, followed by freeze substitution. Samples were infiltrated overnight with LR White embedding resin (London Resin Company Ltd, Reading, Berkshire, England) in ethanol, changed to straight LR White, embedded in gelatin capsules (Electron Microscopy Sciences (EMS), Hatfield, PA, USA), and cured overnight in a UV light cryo-chamber at 4^o^ C. Sections (~50 nm) were placed on grids, treated with 0.05 M glycine for 20 min at RT, rinsed in PBS, blocked for 45 min with 1% bovine serum albumin (EMS), and washed in PBS with 0.1% bovine serum albumin-C (BSA-C) (EMS). For immunogold double labeling, a previously described peptide-specific antiserum directed against DDX6 was affinity purified, desalted, and concentrated [22]. Grids were blocked in 0.5% BSA-C, then incubated with rabbit αDDX6 (0.1 mg/ml in 0.5% BSA-C), followed by goat anti-rabbit F(ab’)2 fragment secondary antibody conjugated to 15nm gold particles (EMS), with washes after each step. Grids were then labeled with the second primary, mouse αGFP (Roche) at 0.2 mg/ml in 0.1% BSAC with 0.002% Tween, followed by goat anti-mouse F(ab’)2 fragment conjugated to 6 nm gold particles (EMS). Fixation, negative staining, imaging with the JEOL-1400 transmission electron microscope, and image acquisition have been described previously [22]. DDX6 antibody was validated previously for EM, including using DDX6 knockdown cells [22].

GFP antibody was validated by showing absence of labeling in non-transfected control cells.

For quantification, images were acquired for ten cells from each of the three groups, with the goal being to analyze similar total PM lengths in each group. Cells were chosen randomly, but excluded for the WT and Gag Zip groups if they had fewer than ten particles at the PM visible at low power. Images encompassed the area of each cell that contained PM assembly sites, with images obtained for ~250 μm of PM total per group. The number of assembly sites analyzed within this ~250 μm of PM are not equivalent since the number of assembly sites depends on VLP phenotype and kinetics. A total of 760 WT events and 409 Gag Zip events were analyzed, but are shown as number of sites per 25 μm PM per cell in S1 Table. Each PM assembly site was scored as genome positive (g+), DDX6+ (D+), or double labeled (g+D+). The following definitions were used for image analysis: early PM assembly sites were defined as displaying curvature at the membrane but with < 50% of a complete bud; late assembly sites at the PM were defined as displaying curvature but with ≥ 50% of a complete bud. If early or late sites contained two or more small gold particles within the full circle defined by the bud, they were scored as g+. If these sites contained one or more large gold particles within a 150 nm perimeter outside the full circle defined by the bud (roughly the size of an RNA granule plus space to account for the antibodies and gold particle bound to an antigen at the periphery of such a granule), then they were scored as D+. S1 Table shows the average number of early, late, and early+late PM assembly events per 25 μm of PM per cell (n = 10 cells +/- SEM), along with the breakdown of how many of these events were g+ (total vs. single-labeled), D+ (total vs. single-labeled), or g+D+ (double-labeled). In italics are g+, D+, and g+D+ per 25 μm of PM per cell as a percentage of the total for each group. Significance was determined on percentage data using a two tailed t-test; not significant was defined as p > 0.01. Labeling of early+late events as a percent of total early+late events is also shown in graphical form in Fig 11B. As described previously [18], the sensitivity of IEM for capturing colocalization is limited by a number of factors including the fact that the 50 nm sections only capture ≤ 50% of a single capsid, which has a diameter of ~100–150 nm.

## Supporting information

S1 Fig VLP production and viral RNA association phenotypes for Gag constructs expressed with VIB *in trans*. **(A)** COS-1 cells were co-transfected to express a WT or mutant codon-optimized Gag tagged with GFP (Gag GFP) and the V1B genome (Set II constructs in Fig 1A), as shown in the diagram. Cell lysates and VLPs were harvested for analysis. **(B)** Equivalent aliquots of cell lysates were analyzed by WB for Gag, as were VLPs harvested from the corresponding cell supernatants. Graph shows the number of unspliced viral RNA copies in VLP pellets from the equivalent of 1000 cells, as determined by RT-qPCR. **(C)** Lysates of transfected cells were also subjected to IP with αGFP or non-immune (N) antibody followed by Gag WB (left), with IP inputs shown (center). IP eluates were also analyzed for unspliced viral RNA copies by RT-qPCR, with NI values subtracted, as shown in graph. Error bars show SEM from duplicate samples. Data are representative of two independent replicate experiments.
(TIF)

S2 Fig Association of ABCE1 with unspliced viral RNA in assembly intermediates. **(A)** COS-1 cells transfected with the indicated plasmids (Set II constructs in Fig 1A) were harvested following PuroHS treatment, and the number of unspliced viral RNA copies per 1000 cells in total cell lysates was determined. **(B)** Lysates from A were analyzed by velocity sedimentation, and the number of unspliced viral RNA copies per 1000 cells in each fraction was determined and normalized to inputs in A. **(C)** Gradient fractions from B were subjected to IP with αGFP, and the number of unspliced viral RNA copies per 1000 cells in IP eluates from each fraction was determined and normalized to inputs in A. Error bars show SEM from duplicate samples. Data in each column are from a single experiment that is representative of three independent replicate experiments.
(TIF)

S3 Fig VLP production and viral RNA association phenotypes for Gag W184A/M185A. **(A)** COS-1 cells were transfected to express WT Gag GFP or Gag W184A/M185A GFP, and the V1B genome (Set II constructs in Fig 1A). **(B)** Cells were harvested following PuroHS treatment, and equivalent aliquots of cell lysates were analyzed by WB for Gag, as were VLPs harvested from the corresponding cell supernatants. Graph shows the copy number of unspliced viral RNA in cell lysate or VLP aliquots corresponding to the equivalent of 1000 cells, as determined by RT-qPCR. **(C)** Lysates of cells transfected as in A were subjected to IP with αGFP or non-immune (N) antibody followed by Gag WB (left). IP eluates were also analyzed by RT-qPCR for copies of unspliced HIV-1 RNA, with NI values subtracted (graph). Error bars show SEM from duplicate samples. Data are representative of two independent replicate experiments. **(D)** Previously we have shown that Gag W184A/M185A is arrested at a membrane-targeted ~80S assembly intermediate when expressed from a proviral construct [19]. To confirm this ~80S arrest for the Gag W184A/M185A GFP plasmid transfected with the V1B genome *in trans*, cells expressing these constructs were analyzed by velocity sedimentation followed by WB for Gag. WB of equivalent aliquots of cell lysates (upper right) shows that Gag W184A/M185A GFP was expressed at higher levels than WT Gag.
(TIF)

S4 Fig Anti-Gag IP fails to detect unspliced HIV-1 RNA from the soluble fraction of H9 T cells chronically infected with HIV-1. **(A)** Human H9 T cells that are chronically infected with HIV-1 were harvested without PuroHS treatment and analyzed by velocity sedimentation to separate complexes in the ~10S to ~750S range. Cell lysates and pooled fractions containing complexes of specific sizes (~10S, ~80S and ~500S) were analyzed by IP with antibody to Gag (αGag, HIV immune globulin) or with a nonimmune control antibody, with unspliced HIV-1 RNA in IP eluates quantified by RT-qPCR. The ~10S pool contains fractions 1–4; the ~80S pool contains fractions 5–11; and the ~500S pool contains fractions 12–18 from a gradient similar to that shown in Fig 6B. Graph shows number of copies of unspliced HIV-1 RNA per 1000 cells in αGag IP eluates (with nonimmune values subtracted) and in IP inputs. The lower limit in the graph (10^3^ copies per 1000 cells) corresponds to the limit of detection of unspliced HIV-1 RNA in standard curves. **(B)** In the same experiment, unfractionated cell lysate was also analyzed by IP with beads but no antibody (No Ab Control). Values for unspliced HIV-1 RNA from the input and IP from the ~10S fraction were below the limit of detection.
(TIF)

S5 Fig Analysis of ΔΨ and Gag Zip constructs. **(A)** COS-1 cells were co-transfected to express WT Gag GFP with either the WT or ΔΨ V1B genomic construct (Set II constructs in Fig 1A). Cell lysates and VLPs were harvested and equivalent aliquots of cell lysates were analyzed by WB for Gag, as were VLPs harvested from the corresponding cell supernatants. Cell lysates and VLPs were were also analyzed by RT-qPCR for unspliced V1B viral RNA. Graph shows the copy number of unspliced viral RNA in VLPs or cell lysate aliquots corresponding to the equivalent of 1000 cells, as determined by RT-qPCR. The experiment was repeated an additional time and data were averaged to generate the graph at far right (Efficiency of ΔΨ packaging) showing packaging of ΔΨ unspliced V1B viral RNA as a % of packaging of WT unspliced V1B viral RNA when data are normalized to unspliced viral RNA in cell lysates (normalized to RNA), or normalized to both unspliced viral RNA in cell lysates and to VLP Gag levels in WBs (normalized to RNA & Gag). Error bars show SEM for n = 2 independent replicate experiments. **(B)** Cell lysates and VLPs were harvested from COS-1 cells transfected with the indicated Gag and GagZip constructs along with the WT V1B genomic construct (Set II constructs in Fig 1A). Equivalent aliquots of cell lysates were analyzed by WB for Gag, as were VLPs harvested from the corresponding cell supernatants. Graph shows the copy number of unspliced viral RNA in VLP or cell lysate aliquots corresponding to the equivalent of 1000 cells, as determined by RT-qPCR. **(C)** COS-1 cells were transfected to express WT Gag GFP or Gag Zip GFP and the V1B genome (Set II constructs in Fig 1A). Cells were harvested following PuroHS treatment and analyzed by velocity sedimentation. Paired gradient fractions were subjected to αGFP IP, followed by WB with HIV immune globulin to allow detection of Gag. **(D)** Lysate from 293T cells (top) or H9 cells (bottom) was analyzed by velocity sedimentation followed by WB of gradient fractions to define the migration of endogenous ABCE1 and DDX6 in human cells.
(TIF)

S6 Fig Gag-DDX6 PLA signal depends on the presence of both primary antibodies. To quantify background signal that is generated by nonspecific binding of either the Gag or DDX6 antibodies used for Gag-DDX6 PLA, positive and negative controls were generated in 293T cells transfected with the HIV-1 provirus expressing WT Gag (Set I construct in Fig 1A). In the Gag-DDX6 positive control, PLA was performed using αGag and αDDX6 antibodies (as in Fig 7). For the Gag-DDX6 negative controls PLA was performed with either αGag or αDDX6 replaced by an isotype-specific non-immune antibody (Negative control 1 and 2, respectively). An additional negative control involved mock transfection of 293T cells followed by PLA performed using αGag and αDDX6 antibodies (Mock transfection). **(A)** Experimental schematic showing the positive and negative control conditions for cells expressing WT provirus. A schematic for the mock-transfected control is not shown. **(B)** The average number of PLA spots per cell was determined for all Gag-positive cells in five randomly chosen fields and normalized to Gag levels. Quantification was only performed for the Gag-transfected positive and negative controls, since there were no comparable Gag transfected cells to analyze in the mock-transfected control, but representative images show almost no signal in mock-transfected controls, as shown in C. **(C)** Shown are representative images for the Gag-transfected positive and negative controls (top three rows), and for the mock-transfected control (bottom row). From left to right for each construct: Gag IF (green) with DAPI-stained nuclei (blue), Gag-DDX6 PLA signal (red) with DAPI-stained nuclei (blue), and a merge of all three. Merge demonstrates that PLA spots are mainly in Gag-expressing cells for the positive control, and PLA spots are largely absent in NI controls. Inset in PLA panel shows a high magnification view of a cell to the left of the inset. Scale bars, 5 μm for main panels, 2.5 μm for inset. Data are representative of three independent replicate experiments. Error bars show SEM (n = 5 cells).
(TIF)

S7 Fig Low intensity DDX6 signal is distinct from P body signal and background. To quantify background signal that is generated by nonspecific binding of the fluorescent-linked secondary antibodies used for DDX6 IF, positive and negative controls were generated in 293T cells transfected with the HIV-1 provirus expressing WT Gag (Set I construct in Fig 1A). In the DDX6 positive control, IF was performed using αDDX6 antibodies as in Fig 10, with a few modifications (see IF methods). Negative control IF was performed following the same protocol, but using only secondary antibody. **(A)** Experimental schematic showing the DDX6 IF and secondary only conditions, both with Gag IF, for cells expressing WT provirus. **(B)** The green signal intensity in red Gag-positive cells was quantified for secondary-only (“Background”); this signal was also quantified in cells that were labeled with primary and secondary as total DDX6 IF signal intensity (“P bodies + Low intensity Signal + Background”) or total DDX6 IF with high intensity P body signal subtracted (“Low intensity Signal + Background”). These signal intensities are shown as percent of mean total DDX6 IF intensity in Gag-positive cells from fields labeled with primary and secondary antibody. **(C)** Shown are representative images for the Gag-transfected cells with DDX6 IF (top three rows), and secondary-only (bottom row). From left to right for each construct: Gag IF (red) with DAPI-stained nuclei (blue), DDX6 IF signal (green) with DAPI-stained nuclei (blue), and a merge of all three. Inset in DDX6 IF central panel shows a high magnification view of a cell above the upper left corner of the inset. **(D)** Histogram shows the distribution of DDX6 signal intensity by pixel number, from the fields used to obtain values shown in B (mean from five fields in the primary plus secondary condition vs. five fields for secondary alone, which was used to obtain background signal). Low intensity signal is seen on the left and high intensity signal is seen on the right. The threshold for high intensity P body signal is shown, as is the cut-off for background signal. Scale bars, 5 μm for main panel and inset. Data are representative of two independent replicate experiments. Error bars show SEM (n = 5 fields). ++ indicates a significant difference between WT Gag and Gag Zip (p<0.005).
(TIF)

S8 Fig Validation of immunoelectron microscopy constructs. **(A)** HeLa cells expressing MCP-GFP were transfected with V1B genomes that contain MS2 binding sites and express WT Gag, G2A, Gag Zip, or MACA (Set IV constructs in Fig 1A). **(B)** Equivalent aliquots of cell lysates were analyzed by WB for Gag, as were VLPs harvested from the corresponding cell supernatants. Graph shows the number of unspliced viral RNA copies in cell lysate or VLP aliquots corresponding to the equivalent of 1000 cells, as determined by RT-qPCR. Error bars show SEM from duplicate samples. Data are representative of two independent replicate experiments.
(TIF)

S1 Table Quantitative immunoelectron microscopy with labeling of genome and DDX6 at the plasma membrane. Early PM Assembly Sites are defined as PM assembly sites displaying less than half a bud, while Late PM Assembly Sites are defined as PM assembly sites displaying half a bud or greater. Shown as number of sites per 25 μm PM per cell (n = 10 cells) are the following: Early + Late PM Sites, Early PM Assembly Sites alone, or Late PM Assembly Sites alone. Labeling sites (g+, D+, and g+D+, as defined below) are shown as number of assembly sites per 25 μm PM per cell (top number), and as % of total PM assembly sites for that group (bottom number, in italics). *ND* indicates % was not calculated because the number of assembly sites was < 1.0 per 25 μm PM. SEM was used to calculate significances for labeling as % of total using a two-tailed Student’s t-test. Shaded columns are shown in the graph in Fig 9B.
(TIF)
