# Supplementary figures and images for: Identifying the assembly intermediate in which Gag first associates with unspliced HIV-1 RNA suggests a novel model for HIV-1 RNA packaging
Source: PLoS Pathog. 2018 Apr 17;14(4):e1006977. doi: 10.1371/journal.ppat.1006977 (PMC5940231; doi:10.1371/journal.ppat.1006977)

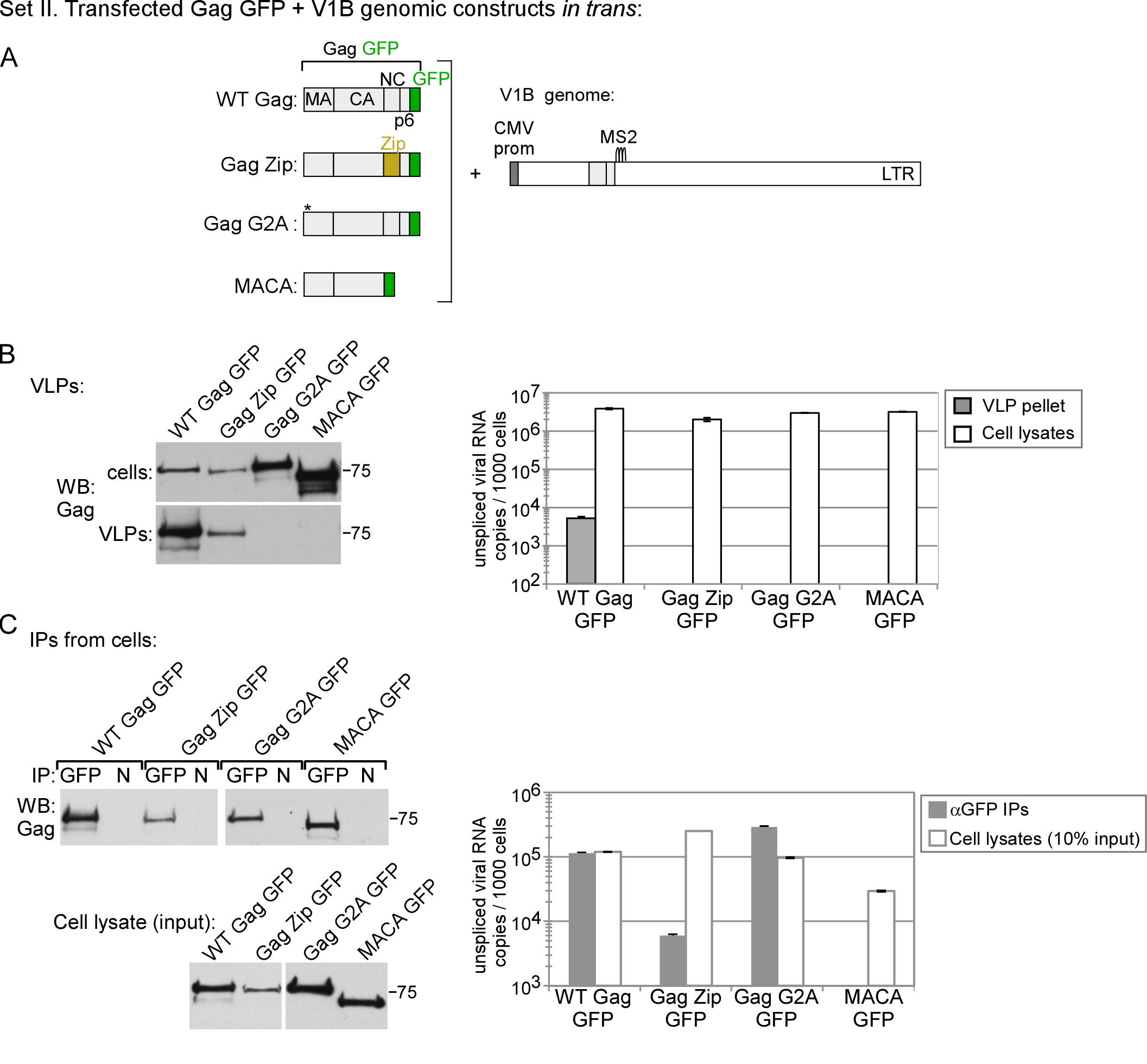

Supplement: S1 Fig — (A) COS-1 cells were co-transfected to express a WT or mutant codon-optimized Gag tagged with GFP (Gag GFP) and the V1B genome (Set II constructs in Fig 1A), as shown in the diagram. Cell lysates and VLPs were harvested for analysis. (B) Equivalent aliquots of cell lysates were analyzed by WB for Gag, as were VLPs harvested from the corresponding cell supernatants. Graph shows the number of unspliced viral RNA copies in VLP pellets from the equivalent of 1000 cells, as determined by RT-qPCR. (C) Lysates of transfected cells were also subjected to IP with αGFP or non-immune (N) antibody followed by Gag WB (left), with IP inputs shown (center). IP eluates were also analyzed for unspliced viral RNA copies by RT-qPCR, with NI values subtracted, as shown in graph. Error bars show SEM from duplicate samples. Data are representative of two independent replicate experiments. (TIF) [file ppat.1006977.s001.tif]

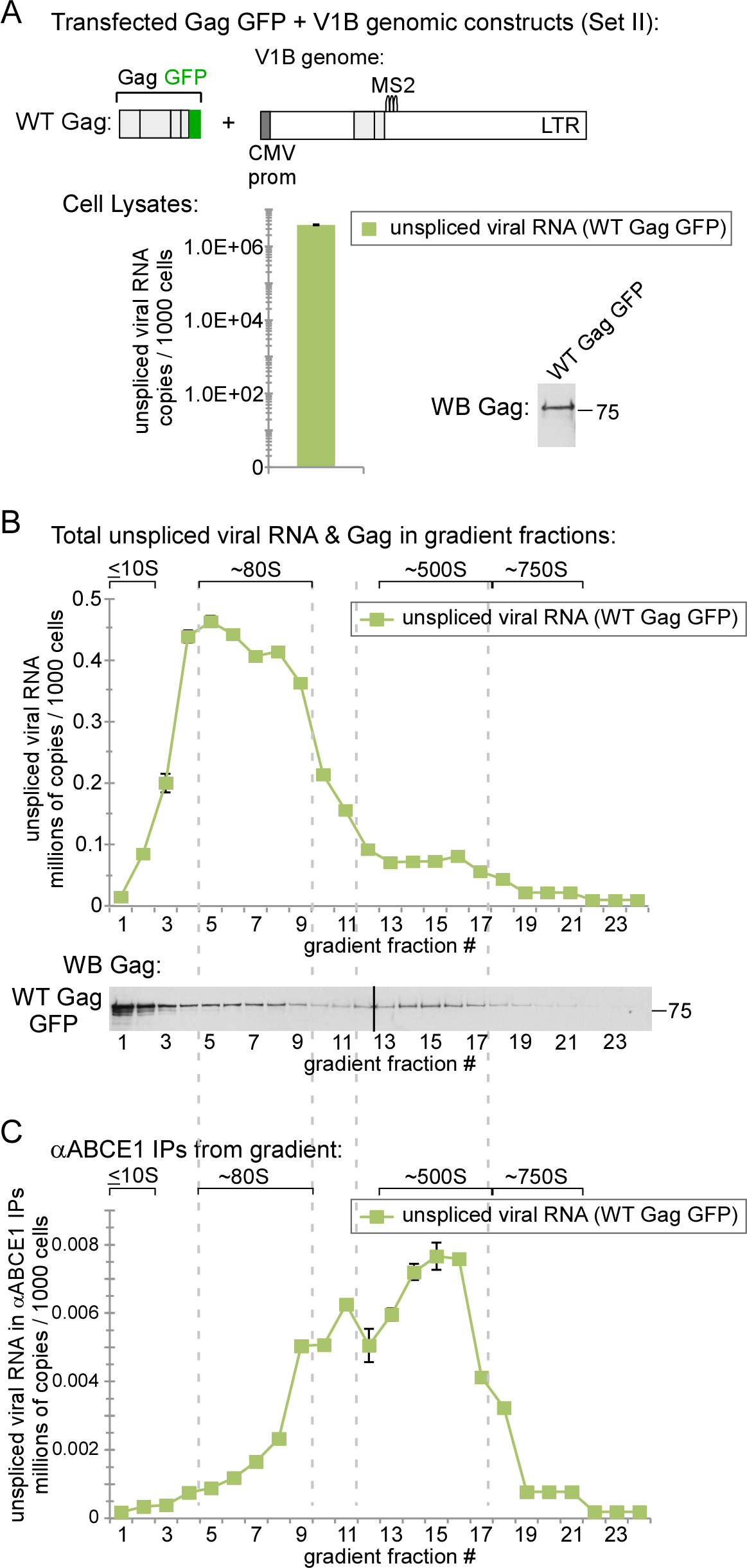

Supplement: S2 Fig — (A) COS-1 cells transfected with the indicated plasmids (Set II constructs in Fig 1A) were harvested following PuroHS treatment, and the number of unspliced viral RNA copies per 1000 cells in total cell lysates was determined. (B) Lysates from A were analyzed by velocity sedimentation, and the number of unspliced viral RNA copies per 1000 cells in each fraction was determined and normalized to inputs in A. (C) Gradient fractions from B were subjected to IP with αGFP, and the number of unspliced viral RNA copies per 1000 cells in IP eluates from each fraction was determined and normalized to inputs in A. Error bars show SEM from duplicate samples. Data in each column are from a single experiment that is representative of three independent replicate experiments. (TIF) [file ppat.1006977.s002.tif]

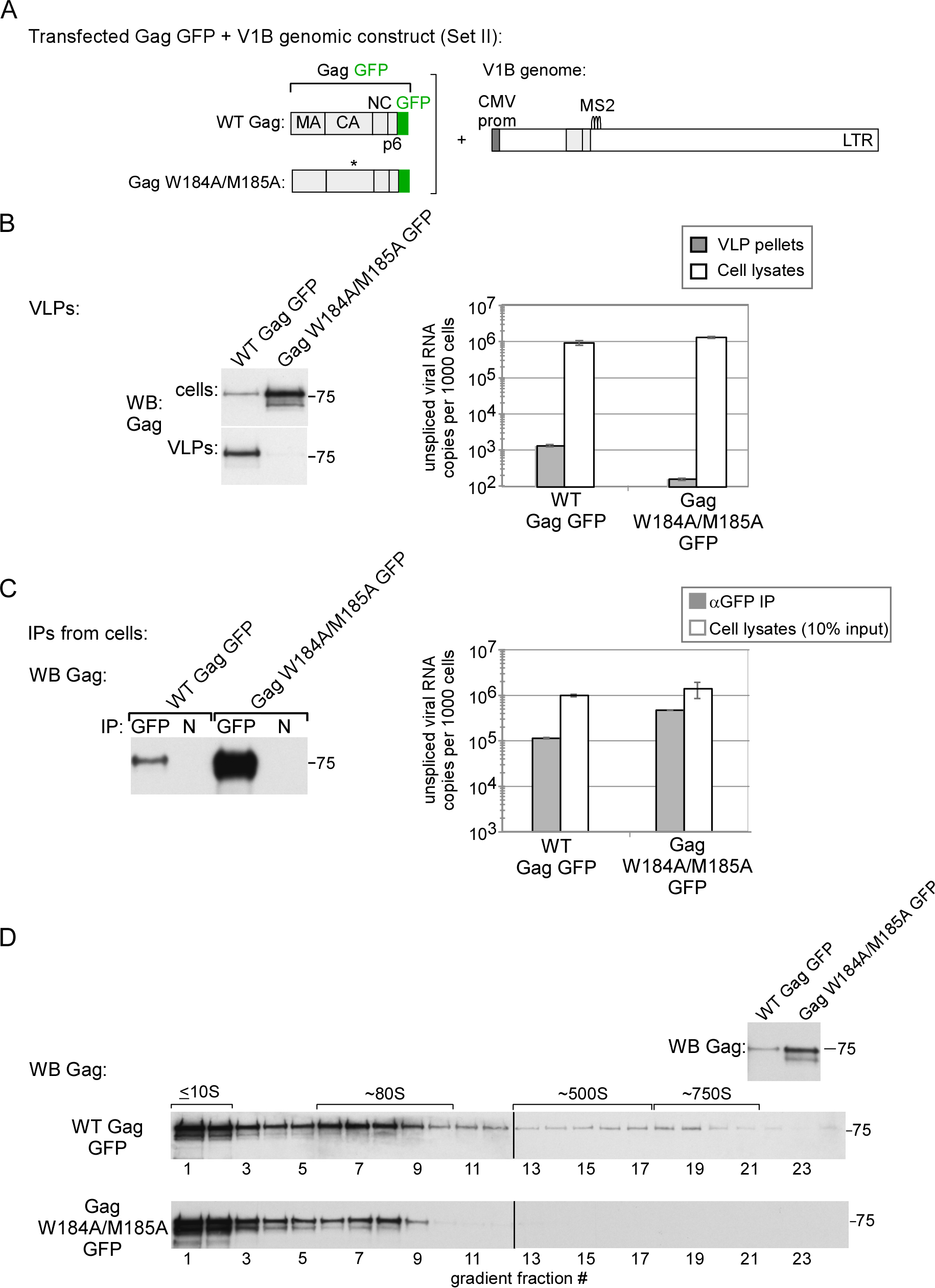

Supplement: S3 Fig — (A) COS-1 cells were transfected to express WT Gag GFP or Gag W184A/M185A GFP, and the V1B genome (Set II constructs in Fig 1A). (B) Cells were harvested following PuroHS treatment, and equivalent aliquots of cell lysates were analyzed by WB for Gag, as were VLPs harvested from the corresponding cell supernatants. Graph shows the copy number of unspliced viral RNA in cell lysate or VLP aliquots corresponding to the equivalent of 1000 cells, as determined by RT-qPCR. (C) Lysates of cells transfected as in A were subjected to IP with αGFP or non-immune (N) antibody followed by Gag WB (left). IP eluates were also analyzed by RT-qPCR for copies of unspliced HIV-1 RNA, with NI values subtracted (graph). Error bars show SEM from duplicate samples. Data are representative of two independent replicate experiments. (D) Previously we have shown that Gag W184A/M185A is arrested at a membrane-targeted ~80S assembly intermediate when expressed from a proviral construct [19]. To confirm this ~80S arrest for the Gag W184A/M185A GFP plasmid transfected with the V1B genome in trans, cells expressing these constructs were analyzed by velocity sedimentation followed by WB for Gag. WB of equivalent aliquots of cell lysates (upper right) shows that Gag W184A/M185A GFP was expressed at higher levels than WT Gag. (TIF) [file ppat.1006977.s003.tif]

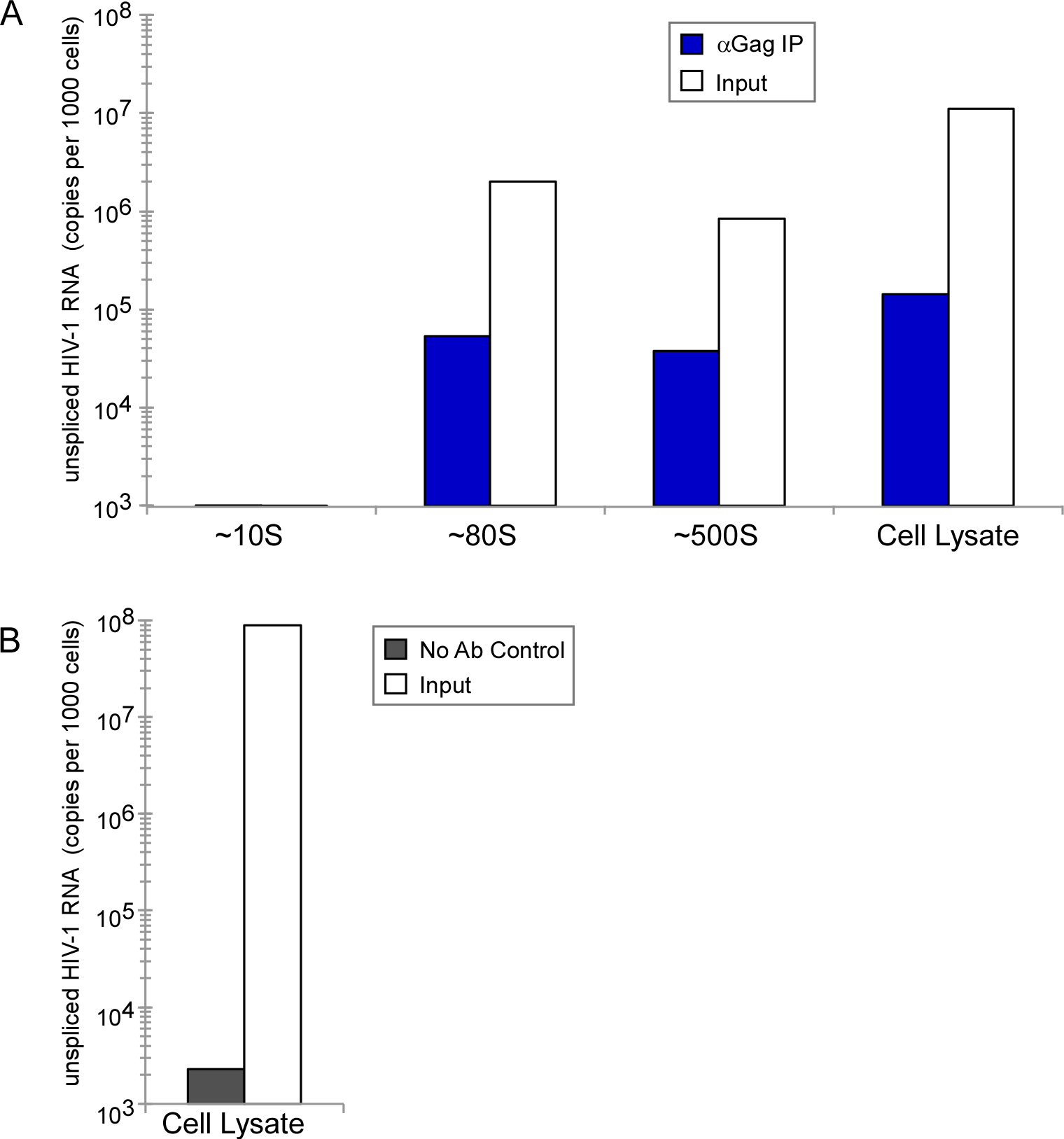

Supplement: S4 Fig — (A) Human H9 T cells that are chronically infected with HIV-1 were harvested without PuroHS treatment and analyzed by velocity sedimentation to separate complexes in the ~10S to ~750S range. Cell lysates and pooled fractions containing complexes of specific sizes (~10S, ~80S and ~500S) were analyzed by IP with antibody to Gag (αGag, HIV immune globulin) or with a nonimmune control antibody, with unspliced HIV-1 RNA in IP eluates quantified by RT-qPCR. The ~10S pool contains fractions 1–4; the ~80S pool contains fractions 5–11; and the ~500S pool contains fractions 12–18 from a gradient similar to that shown in Fig 6B. Graph shows number of copies of unspliced HIV-1 RNA per 1000 cells in αGag IP eluates (with nonimmune values subtracted) and in IP inputs. The lower limit in the graph (103 copies per 1000 cells) corresponds to the limit of detection of unspliced HIV-1 RNA in standard curves. (B) In the same experiment, unfractionated cell lysate was also analyzed by IP with beads but no antibody (No Ab Control). Values for unspliced HIV-1 RNA from the input and IP from the ~10S fraction were below the limit of detection. (TIF) [file ppat.1006977.s004.tif]

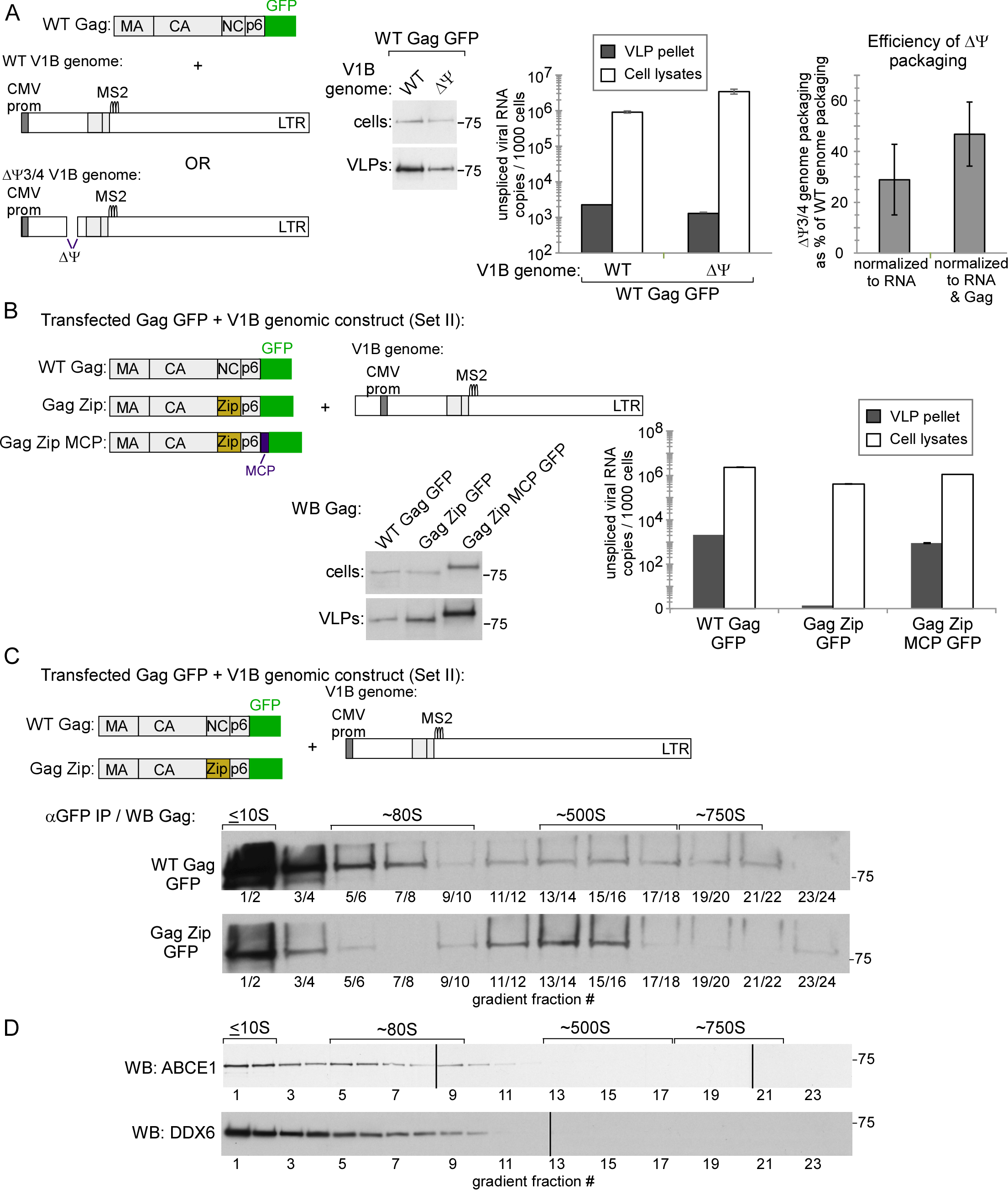

Supplement: S5 Fig — (A) COS-1 cells were co-transfected to express WT Gag GFP with either the WT or ΔΨ V1B genomic construct (Set II constructs in Fig 1A). Cell lysates and VLPs were harvested and equivalent aliquots of cell lysates were analyzed by WB for Gag, as were VLPs harvested from the corresponding cell supernatants. Cell lysates and VLPs were were also analyzed by RT-qPCR for unspliced V1B viral RNA. Graph shows the copy number of unspliced viral RNA in VLPs or cell lysate aliquots corresponding to the equivalent of 1000 cells, as determined by RT-qPCR. The experiment was repeated an additional time and data were averaged to generate the graph at far right (Efficiency of ΔΨ packaging) showing packaging of ΔΨ unspliced V1B viral RNA as a % of packaging of WT unspliced V1B viral RNA when data are normalized to unspliced viral RNA in cell lysates (normalized to RNA), or normalized to both unspliced viral RNA in cell lysates and to VLP Gag levels in WBs (normalized to RNA & Gag). Error bars show SEM for n = 2 independent replicate experiments. (B) Cell lysates and VLPs were harvested from COS-1 cells transfected with the indicated Gag and GagZip constructs along with the WT V1B genomic construct (Set II constructs in Fig 1A). Equivalent aliquots of cell lysates were analyzed by WB for Gag, as were VLPs harvested from the corresponding cell supernatants. Graph shows the copy number of unspliced viral RNA in VLP or cell lysate aliquots corresponding to the equivalent of 1000 cells, as determined by RT-qPCR. (C) COS-1 cells were transfected to express WT Gag GFP or Gag Zip GFP and the V1B genome (Set II constructs in Fig 1A). Cells were harvested following PuroHS treatment and analyzed by velocity sedimentation. Paired gradient fractions were subjected to αGFP IP, followed by WB with HIV immune globulin to allow detection of Gag. (D) Lysate from 293T cells (top) or H9 cells (bottom) was analyzed by velocity sedimentation followed by WB of gradient fractions to define the migration of [file ppat.1006977.s005.tif]

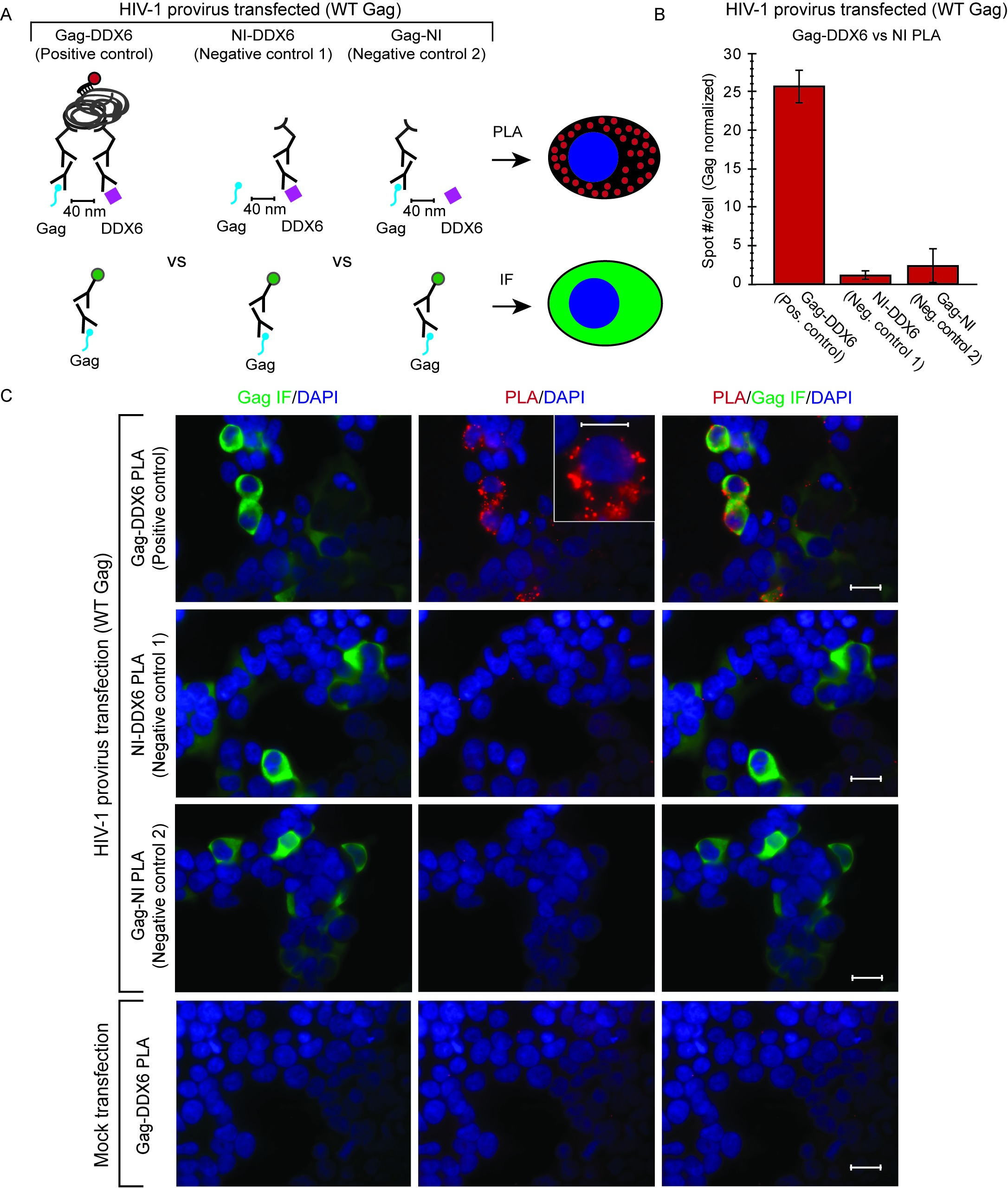

Supplement: S6 Fig — To quantify background signal that is generated by nonspecific binding of either the Gag or DDX6 antibodies used for Gag-DDX6 PLA, positive and negative controls were generated in 293T cells transfected with the HIV-1 provirus expressing WT Gag (Set I construct in Fig 1A). In the Gag-DDX6 positive control, PLA was performed using αGag and αDDX6 antibodies (as in Fig 7). For the Gag-DDX6 negative controls PLA was performed with either αGag or αDDX6 replaced by an isotype-specific non-immune antibody (Negative control 1 and 2, respectively). An additional negative control involved mock transfection of 293T cells followed by PLA performed using αGag and αDDX6 antibodies (Mock transfection). (A) Experimental schematic showing the positive and negative control conditions for cells expressing WT provirus. A schematic for the mock-transfected control is not shown. (B) The average number of PLA spots per cell was determined for all Gag-positive cells in five randomly chosen fields and normalized to Gag levels. Quantification was only performed for the Gag-transfected positive and negative controls, since there were no comparable Gag transfected cells to analyze in the mock-transfected control, but representative images show almost no signal in mock-transfected controls, as shown in C. (C) Shown are representative images for the Gag-transfected positive and negative controls (top three rows), and for the mock-transfected control (bottom row). From left to right for each construct: Gag IF (green) with DAPI-stained nuclei (blue), Gag-DDX6 PLA signal (red) with DAPI-stained nuclei (blue), and a merge of all three. Merge demonstrates that PLA spots are mainly in Gag-expressing cells for the positive control, and PLA spots are largely absent in NI controls. Inset in PLA panel shows a high magnification view of a cell to the left of the inset. Scale bars, 5 μm for main panels, 2.5 μm for inset. Data are representative of three independent replicate experiments. Error bars show SEM [file ppat.1006977.s006.tif]

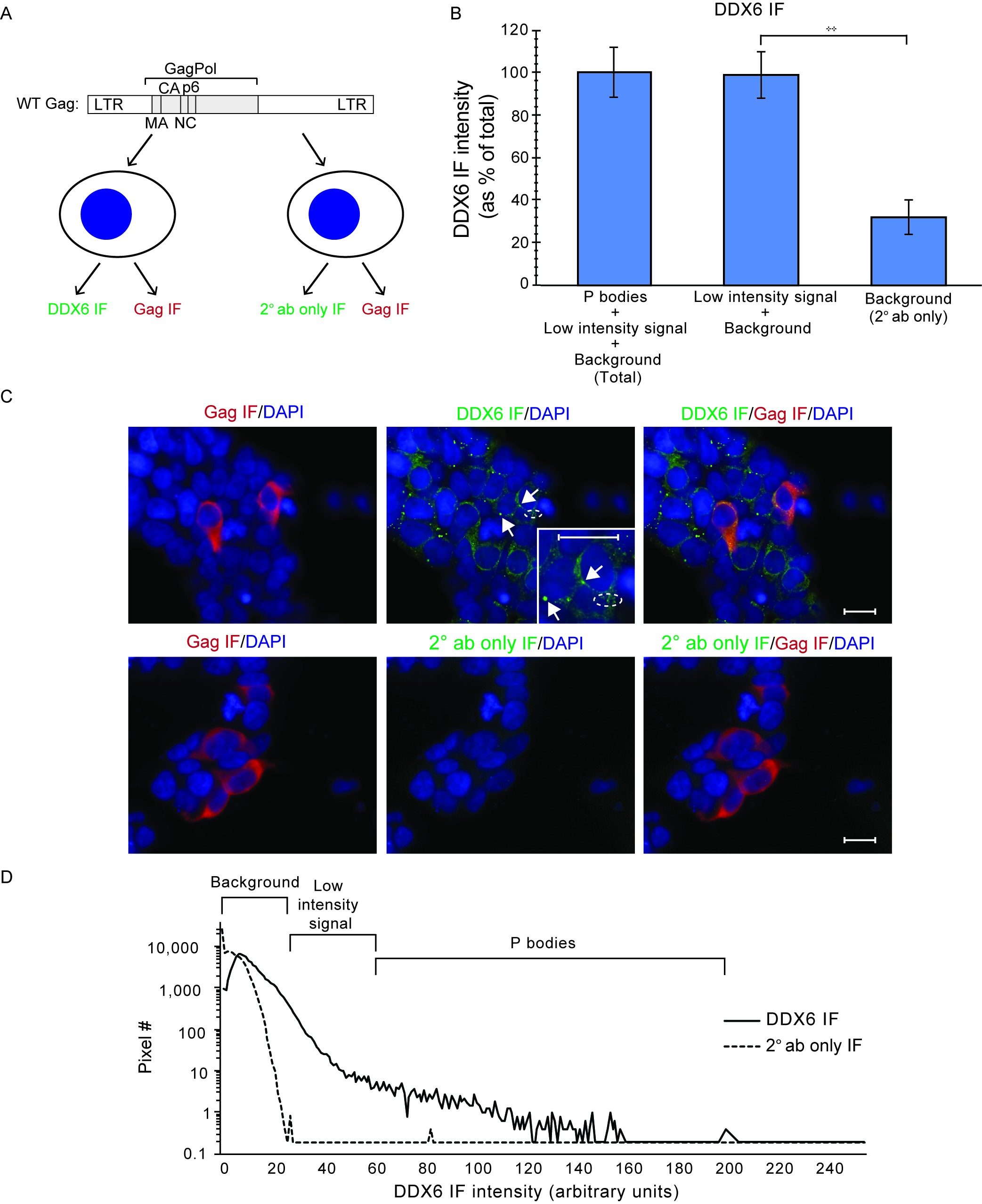

Supplement: S7 Fig — To quantify background signal that is generated by nonspecific binding of the fluorescent-linked secondary antibodies used for DDX6 IF, positive and negative controls were generated in 293T cells transfected with the HIV-1 provirus expressing WT Gag (Set I construct in Fig 1A). In the DDX6 positive control, IF was performed using αDDX6 antibodies as in Fig 10, with a few modifications (see IF methods). Negative control IF was performed following the same protocol, but using only secondary antibody. (A) Experimental schematic showing the DDX6 IF and secondary only conditions, both with Gag IF, for cells expressing WT provirus. (B) The green signal intensity in red Gag-positive cells was quantified for secondary-only (“Background”); this signal was also quantified in cells that were labeled with primary and secondary as total DDX6 IF signal intensity (“P bodies + Low intensity Signal + Background”) or total DDX6 IF with high intensity P body signal subtracted (“Low intensity Signal + Background”). These signal intensities are shown as percent of mean total DDX6 IF intensity in Gag-positive cells from fields labeled with primary and secondary antibody. (C) Shown are representative images for the Gag-transfected cells with DDX6 IF (top three rows), and secondary-only (bottom row). From left to right for each construct: Gag IF (red) with DAPI-stained nuclei (blue), DDX6 IF signal (green) with DAPI-stained nuclei (blue), and a merge of all three. Inset in DDX6 IF central panel shows a high magnification view of a cell above the upper left corner of the inset. (D) Histogram shows the distribution of DDX6 signal intensity by pixel number, from the fields used to obtain values shown in B (mean from five fields in the primary plus secondary condition vs. five fields for secondary alone, which was used to obtain background signal). Low intensity signal is seen on the left and high intensity signal is seen on the right. The threshold for high intensity P body signal is shown, a [file ppat.1006977.s007.tif]

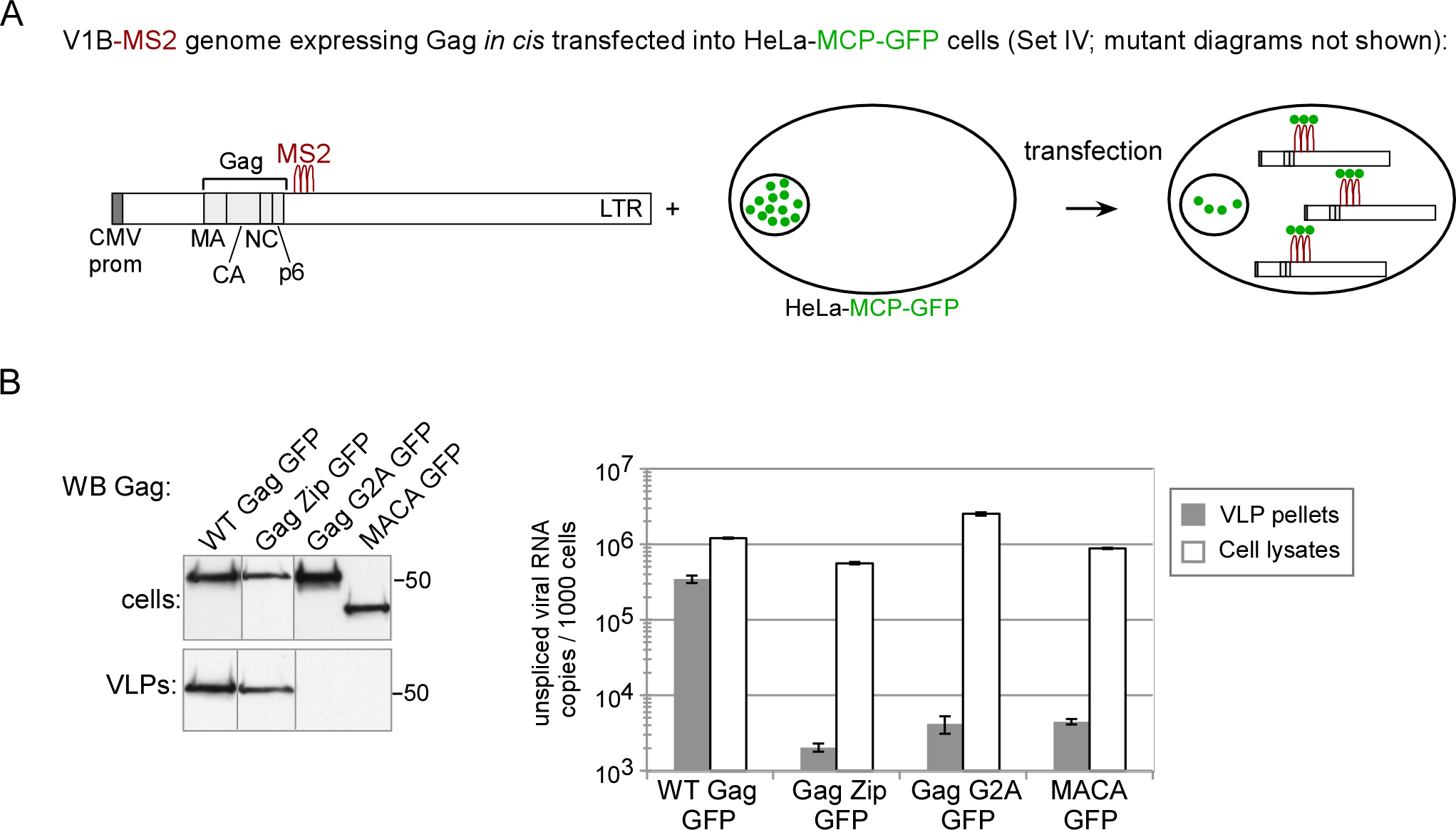

Supplement: S8 Fig — (A) HeLa cells expressing MCP-GFP were transfected with V1B genomes that contain MS2 binding sites and express WT Gag, G2A, Gag Zip, or MACA (Set IV constructs in Fig 1A). (B) Equivalent aliquots of cell lysates were analyzed by WB for Gag, as were VLPs harvested from the corresponding cell supernatants. Graph shows the number of unspliced viral RNA copies in cell lysate or VLP aliquots corresponding to the equivalent of 1000 cells, as determined by RT-qPCR. Error bars show SEM from duplicate samples. Data are representative of two independent replicate experiments. (TIF) [file ppat.1006977.s008.tif]

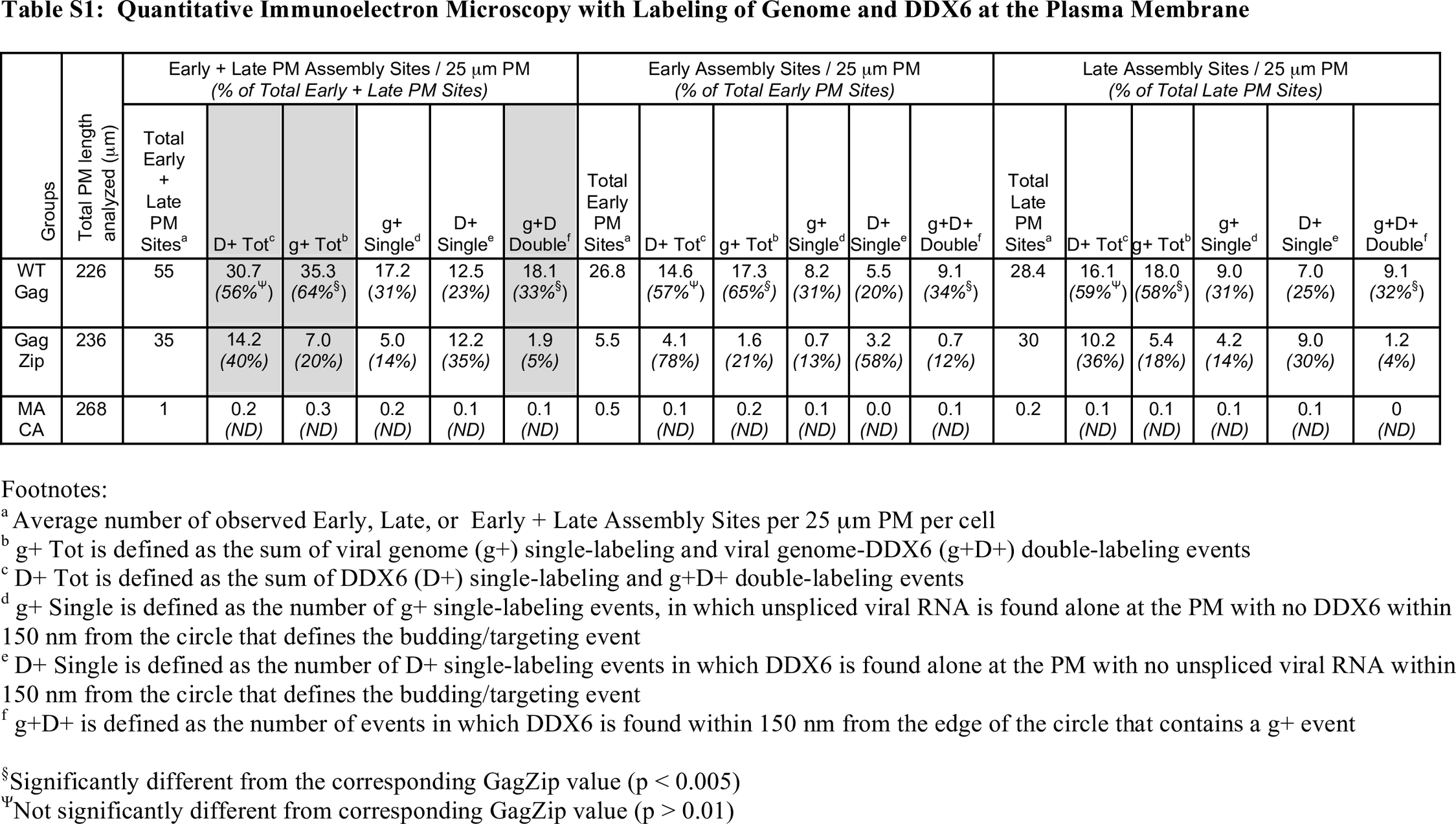

Supplement: S1 Table — Early PM Assembly Sites are defined as PM assembly sites displaying less than half a bud, while Late PM Assembly Sites are defined as PM assembly sites displaying half a bud or greater. Shown as number of sites per 25 μm PM per cell (n = 10 cells) are the following: Early + Late PM Sites, Early PM Assembly Sites alone, or Late PM Assembly Sites alone. Labeling sites (g+, D+, and g+D+, as defined below) are shown as number of assembly sites per 25 μm PM per cell (top number), and as % of total PM assembly sites for that group (bottom number, in italics). ND indicates % was not calculated because the number of assembly sites was < 1.0 per 25 μm PM. SEM was used to calculate significances for labeling as % of total using a two-tailed Student’s t-test. Shaded columns are shown in the graph in Fig 9B. (TIF) [file ppat.1006977.s009.tif]
